# Supplementary material for: A double-pointed wooden throwing stick from Schöningen, Germany: Results and new insights from a multianalytical study
Source: PLoS One. 2023 Jul 19;18(7):e0287719. doi: 10.1371/journal.pone.0287719 (PMC10355447; doi:10.1371/journal.pone.0287719)
Supplement: S1 File — (DOCX) [file pone.0287719.s001.docx]

**S1 Supporting Information**

**A double-pointed wooden throwing stick from** **Schöningen, Germany: results and new insights from a multianalytical study**

**Authors** Annemieke Milks^1^, Jens Lehmann^2^, Dirk Leder^2,^ Michael Sietz^3^, Tim Koddenberg^4^, Utz Böhner^5^**,** Volker Wachtendorf^6^, Thomas Terberger^2,7^

^1^ Department of Archaeology, University of Reading.^2^ Department of Hunter-Gatherer Archaeology, Niedersächsisches Landesamt für Denkmalpflege (Lower Saxony State Office for Cultural Heritage). ^3^ Archaeological Conservation Department, Niedersächsisches Landesamt für Denkmalpflege (Lower Saxony State Office for Cultural Heritage). ^4^ Wood Biology and Wood Products, Faculty of Forest Sciences and Forest Ecology, University of Göttingen. ^5^ Inventory and Heritage Atlas, Niedersächsisches Landesamt für Denkmalpflege (Lower Saxony State Office for Cultural Heritage). ^6^ Bundesanstalt für Materialforschung und -prüfung (BAM), Unter den Eichen 87, 12205 Berlin, German ^7^ Department of Prehistoric Archaeology, University of Göttingen.

**1. Supplementary Methods**

**1.1 3D microscopy**

3D microscopy has been developed for the analysis of Pleistocene bone modifications and technologies (e.g. 1–3), but to our knowledge has not yet been applied to wooden tools. The use of this analytical tool provides means of assessing external features of wood, including human and taphonomic modifications. The double-pointed stick was microscopically examined with the 3D reflected light microscope Keyence VHX-5000 (Keyence, Neu-Isenburg, Germany). The image collection was performed at the Research Museum Paläon in Schöningen, Germany and at the Department of Wood Biology and Wood Products, University of Göttingen. The microscope system is equipped with a VHX-ZST dual objective zoom lens. The Keyence VHX-5000 makes it possible to obtain true-colour images of the observation surface non-destructively and with a high depth of field enabling high-quality 3D images. To obtain 3D images, the focal planes were manually adjusted for the lowest and highest focus points. Subsequently, the microscope software automatically stitched together multiple in-focus images at different focal planes between the set focus points in the z-direction (i.e., height). By this means, either single images or panoramic composite images were captured at magnifications between 20× to 500×. Composite images are multiple single 2D and 3D images stitched together by the software. In the case of 3D images, the colour of 3D representations provides information about the height with blue representing the lowest focus point and red the highest focus point. All images were saved as TIFF files. Based on 3D images, profile cross-sections were obtained on selected surface micro-features perpendicular to the length allowing morphometric analyses (e.g. width, depth).

**1.2 micro-CT**

Similar to 3D microscopy, the application of high resolution micro-Computed Tomography (micro-CT) is in its infancy for analysing wooden tools, though it has been applied to bone tools (1,3). It provides a non-invasive means of evaluating the raw material features, alongside a clear way of analysing the manufacturing techniques and natural and taphonomic alterations. X-ray micro-CT scans of the entirety of the double-pointed stick were performed by Waygate Technologies GmbH with the micro-CT scanner phoenix V│tome│xm. The object was scanned in sections, with scan parameters for each segment found in Table S1. The acquired micro-CT data were processed and imaged using VGSTUDIO MAX 3.3.4 software at the NLD and with the Avizo software (FEI, Thermo Fisher Scientific, Hillsboro, Oregon, USA) at the Department of Wood Biology and Wood Products, University of Göttingen.

**Table S1.** Scan parameters for each segment.

|  | **Voltage** | **Current** | **Voxel size** |
| --- | --- | --- | --- |
| Point 1 | 80 kV | 110 uA | 8.23 um^3^ |
| Fragment 1 | 90 kV | 180 uA | 34.47 um^3^ |
| Fragment 2 | 110 kV | 110 uA | 29.65 um^3^ |
| Fragment 2_2 | 110 kV | 110 uA | 29.65 um^3^ |

**1.3 ATR-FTIR**

A Bruker IR-microscope (model “LUMOS II”) was used for the ATR-FTIR analysis. The ATR mode was applied, which means that the ATR tip is driven out of the centre of VIS lens at the measurement position which was chosen before with the VIS part of the microscope. Parameters applied were 512 sample scans, and 128 background scans. Before each sample measurement the tip was cleaned. Resolution was 4 cm-1. Corrections caried out were atmospheric correction (CO2, H2O), ATR correction and an automatic straight baseline correction as well as calculating absorbance out of ATR data using the Bruker OPUS software. The ATR tip was set to press at the lowest possible pressure to avoid damage to the sample; the scan rate consequently had to be set at a comparatively high level. Measurement spot size was 50 µm. The double-pointed stick was placed on a stainless-steel rack with pieces of polyurethane foam relating the movement of the rack to the stick.

To better evaluate the impact of the conservation treatment (Kauramin 800™) one modern wood sample treated with this material (see also 4). The resin was freshly prepared, was still sticky, and not fully cured. This sample was investigated with a glass slide and standard microscope. As the artefact is not a flat surface but rather cylindrical and conical, measurements were taken as close as possible to the crest. If charring was the reason for the darkening on the throwing stick, then the generation of graphene should produce aromaticity absorption peaks and the oxidation should produce oxidised species like carbonyls (5). The formation of mould should produce absorption band for lipids, protein, carbohydrates and phosphate (6).


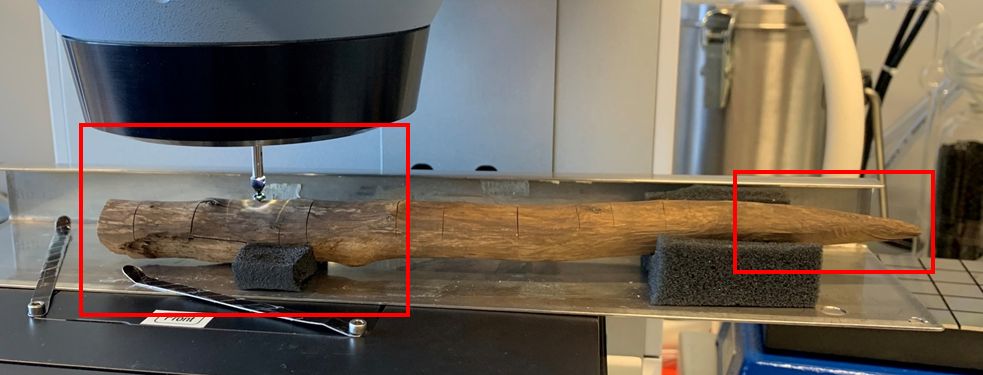


**Figure S1.** Photo of the setup for the IR measurements of the throwing stick with the left red rectangle showing the measurement are in the middle and the right rectangle that of the tip of the stick. ATR crystal extended to the stick’s surface.


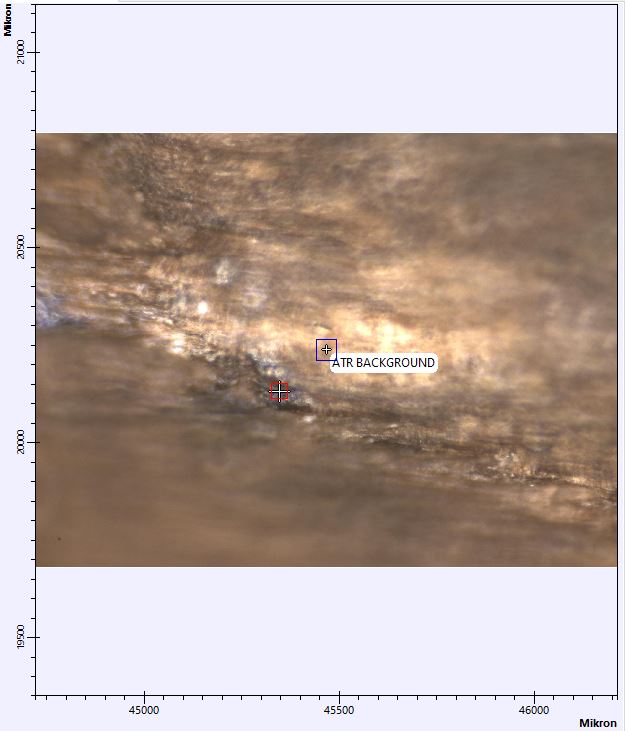


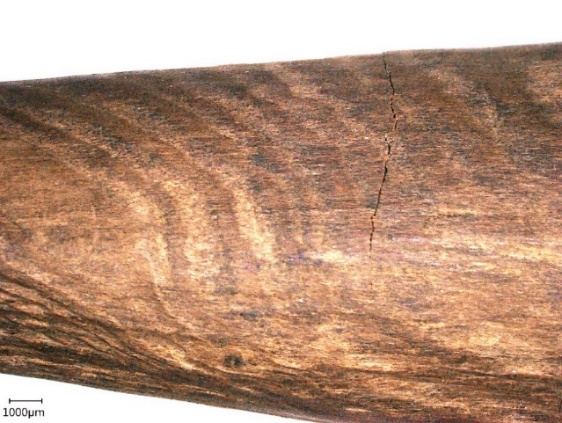


**Figure S2.** Example of a darkened area (Position 8) subjected to analysis. IR-microscopic picture left, light microscopic picture right. IR-measurement area size was about 50 µm.


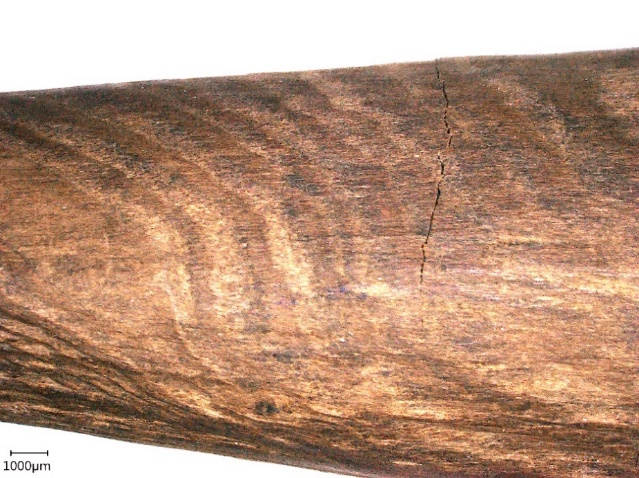

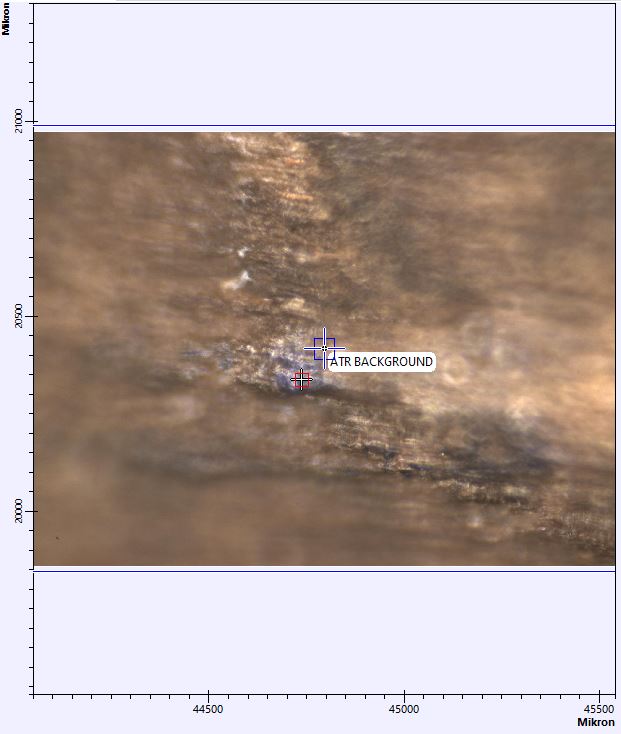


**Figure S3.** Example light spot (Position 6). IR-microscopic picture left, light microscopic picture right. IR-measurement spot size was about 50 µm.

**Table S2.** IR peak assignments wood and charcoal

| **Wavenumber / cm^-1^** | **Functional group** | **Source** |
| --- | --- | --- |
| 817 | two adjacent aryl CH wag | (7) |
| 855 | lone aryl CH wag | (7) |
| 895 | C-H stretching, in cellulose and hemicellulose, decreasing from wood to chars | (8) |
| 1030 | Si-O-Si vibration of clay minerals (charcoal) | (9) |
| 1030 | C-O stretching, in cellulose and hemicellulose, decreasing from wood to chars | (8) |
| 1153 | C-O-C stretching, in cellulose and hemicellulose, decreasing from wood to chars | (8) |
| 1232 | C-O of lignin, decreasing with temperature |  |
| 1260 | C-O vibration of carboxylic acids (charcoals) | (9) |
| 1319 | C-H lignin, decreasing with temperature | (8) |
| 1361 | C-H stretching, in cellulose and hemicellulose, decreasing from wood to chars | (8) |
| 1380 | symmetric COO-stretch (carboxylates) | (10) |
| 1380 | C-H stretch vibration of the methyl-group | (10) |
| 1419 | C-H lignin, decreasing with temperature | (8) |
| 1456 | C-H lignin, decreasing with temperature | (8) |
| 1506 | aromatic skeletal vibration of lignin, increasing to about 540 °C | (8) |
| 1513 | aromatic ting vibrations lignin | (7) |
| 1575 | asymmetric COO-stretch (carboxylates) | (10) |
| 1593 | aromatic skeletal vibration of lignin, increasing to about 540 °C | (8) |
| 1597 | aromatic ting vibrations lignin | (7) |
| 1610 (alongside 1900, 752, 820 and 880 cm^-1^) | new aromatic absorptions due to charring | (5) |
| 1635 | OH-groups cellulose II, increased absorbance with increasing humidity | (11) |
| 1708 (shoulder) | C-O vibration of carboxylic acids (charcoals, grows with age) | (9) |
| 1734 | C=O stretching, in cellulose and hemicellulose, decreasing from wood to chars | (8) |
| 2904 | aliph. CH_2_, aromat CH_3_ | (8) |
| 3338 (broad) | O-H stretching hydrogen bonding | (8) |
| 3400 | O-H vibrations | (10) |

**1.4 3D models**

The 3D models of the two fragments were constructed on the basis of two scans, made in 2021 by GOM Metrology (Brunswick) using the ATOS 5 optical High-Speed 3D Scanning System (single point accuracy = 0.0015 mm, maximum gap = 0.322 mm, unit edge length = 0.08 mm). The two fragments were merged using Blender. The model can be viewed and accessed using the following link:

[Schöningen 13 II double pointed stick - 3D model by Denkmalatlas Niedersachsen (@denkmalatlas) [b3f2f12] (sketchfab.com)](https://sketchfab.com/3d-models/schoningen-13-ii-double-pointed-stick-b3f2f1267d4a4dd7bd7fd3608f65fec4)

**1.5 *Chaîne opératoire* framework**

**Table S3. Definition of Phases as used throughout the manuscript and SI, with examples of evidence from wood analysis**

| **Phase** | **Definition** | **Examples of evidence*** |
| --- | --- | --- |
| **Phase 0** | Raw Material | - wood species - blank selection (e.g. branch, trunk) - analysis of annual rings and other growth features |
| **Phase 1** | Manufacture | - roughing out - seasoning - working traces (e.g. tool marks) - shaping |
| **Phase 2** | Use, Maintenance and Discard | - use traces - curation - use fractures - discard |
| **Phase 3** | Taphonomy | - trampling - weathering - post-depositional alterations to morphology (e.g. taphonomic compression) |
| **Phase 4** | Excavation and post-excavation | - mineralisation - excavation damage - conservation and post-conservation alterations |

**1.6 Ethnographic review**

A literature review was conducted using search terms ‘hunting stick’ OR ‘throwing stick’, without limitation on subsistence type, using the eHRAF World Cultures database. We excluded examples that only pertained to games or toys, where use was only associated with ritual purposes, or where their use was as part of lore or oral traditions, unless it was noted from other sources that these societies had used such tools for hunting and/or violence. Additional sources from our own library and bibliographies were also included. Suitable excerpts were coded for location, use, prey type, and morphometrics, the results of which are available in S4 Dataset.

Puccioni (12) reported that throwing sticks used by Somalian Pastoralists were used alongside javelins, with the only demonstrable difference being length. Data recorded of Daasanach pastoralists throwing sticks demonstrate distances of over 100 metres, and velocities of 36 m/s (13), which are well in excess of any estimates of javelin throwing based on experiments and ethnography (14,15). Manufacturing techniques included use of a coarse stone, likely to shape and finish using abrasion (16), and the addition of gum to make it heavier and/or repel water (17,18).

**1.7 Morphological description**

Morphological descriptions of the throwing stick follow methods and terms in Bordes (19). Specific to this object include morphological classification on the basis of profile (e.g. circular, oval, elliptical), type and symmetry of form (e.g. straight shape, curved shape with enlarged head, crescent), and end type (e.g. pointed, bevelled, rounded).

**1.8 Trace analysis**

In addition to referenced terms and images provided in the open access Wood Technology Glossary and Code (20), further reference images are provided in this document of traces and fractures that underpin the analysis of the archaeological specimen.

**Phase 0**


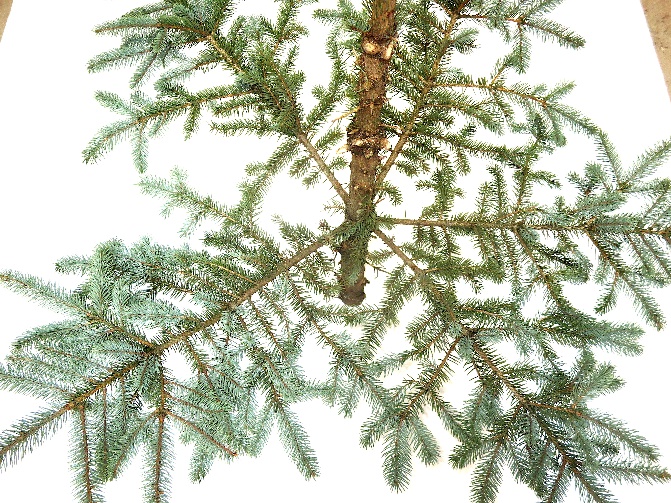


**Figure S4.** Photograph of a branch whorl on a spruce tree. Photo: J. Lehmann


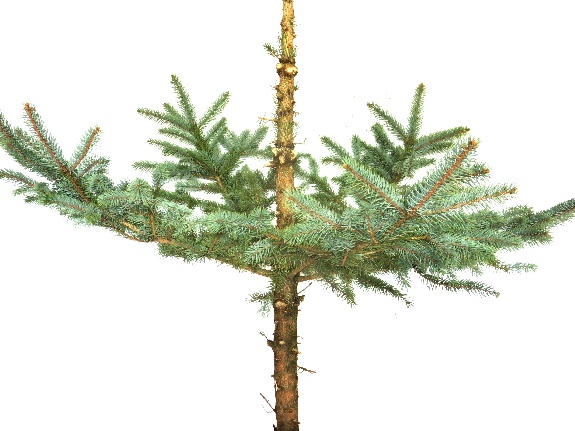


**Figure S5.** Photograph showing typical curvature of branch growth on a spruce tree. Photo: J. Lehmann


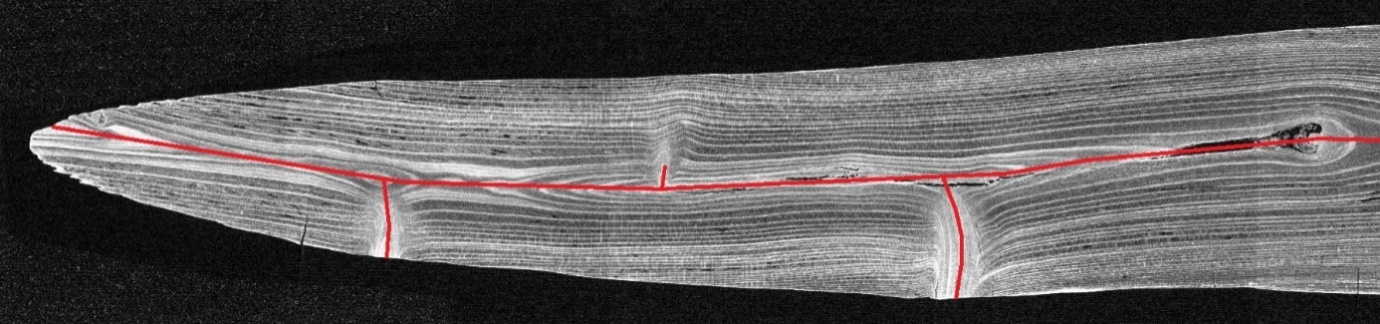


**Figure S6**. Longitudinal micro-CT slice of Point 1. The angle of auxiliary branches growth from the pith shows that the branch originally grew from Point 1 in direction to Point 2 (i.e. from right to left). Micro-CT: Waygate. Image: T. Koddenberg.

**Table S4**. Features on the double-pointed stick connected to Phase 0.

| **Feature** | **View** | **Location** |
| --- | --- | --- |
| dead limb/knot hole | A3  A2  A2­­­­­­–A3      A1­­­­­­–A2  A4  A1  A2  A4  A1  A3­­­­­­–A4 | 22 cm  28 cm  36 cm  38 cm  47 cm  49 cm  51 cm  57 cm  59 cm  60 cm  61 cm  62 cm |
| pith | A2­­­­­­–A3 | 0–0.5 cm  76–77 cm |

**Phase 1**


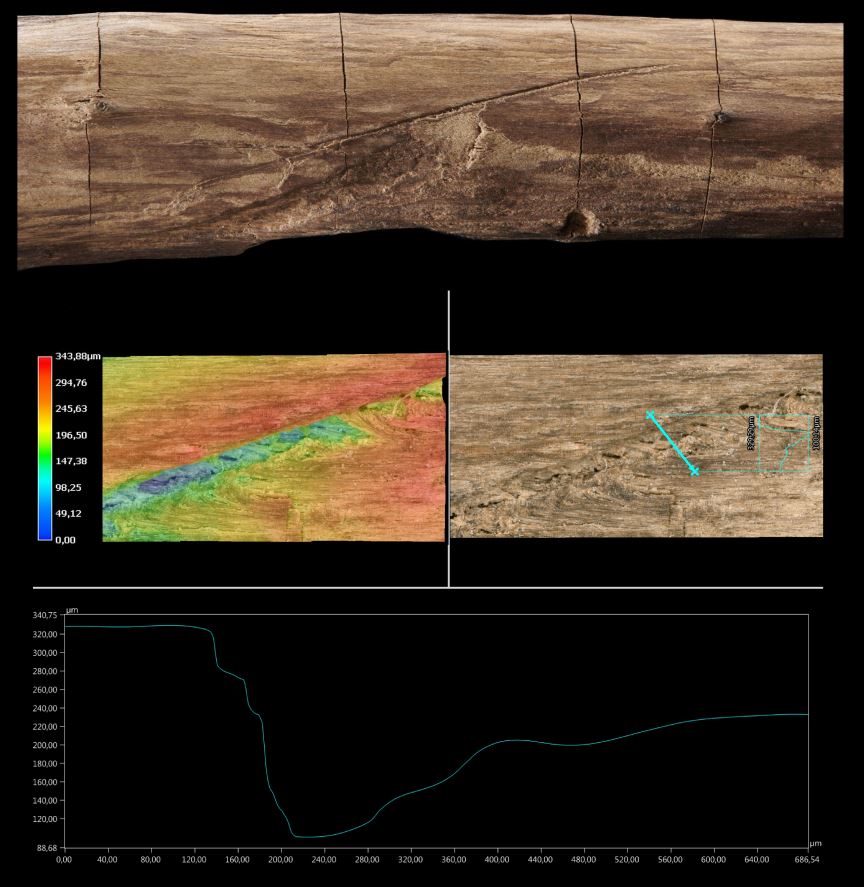


**Figure S7.** Detail of the long and curved cut mark. View A3. at 14–20.5 cm. b. The bent fibres on the right cut edge show the cutting direction against the grain course in the direction of tip 2, while the other edge has been cut with the grain. Photo: V. Minkus. 3D microscopy images: T. Koddenberg.


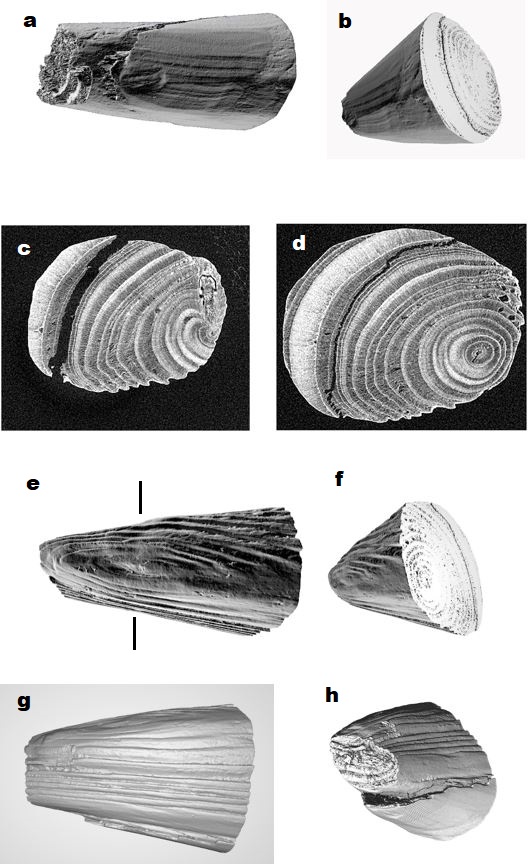


**Figure S8. Further Technological details of Point 1** 76.1 to 77.2 cm. Greyscale images were generated from the micro-CT scans. **a.** View A4. Detail of recently broken tip and stress crack along a single annual ring (Phase 4). Also visible are tool marks created by scraping (Phase 1). **b.** View A1­­­­­­–A4. Surface facets are visible by contrast in light and shadow on the point surface and in cross-section. **c.** Cross-section at approx. 76.7 cm. The recent crack is clearly visible, the pith emerges (A2­­­­­­–A3) and a dead knot is also identifiable. **d.** Cross-section at approx. 76.1 cm. The surface facets are visible along the left and top edges (Views A1-A4 and A2­­­­­­–A3). **e. View** A2­­­­­­–A3. The pith is clearly visible in the centre of the tangential annual ring surface. **f.** View A2­­­­­­–A3 illustrates the cross-section at 76.1 cm, showing the pith emerging near the worked surface. **G. View** A3 shows an example of radial annual rings, which unlike the arched tangential annual rings, are linear in nature. **h.** The broken tip at 77.2 cm. The upwardly curved annual rings in the cross-section of the broken tip show that at the point there is an absence of the pith. CT: Waygate Technologies. Image: J. Lehmann.


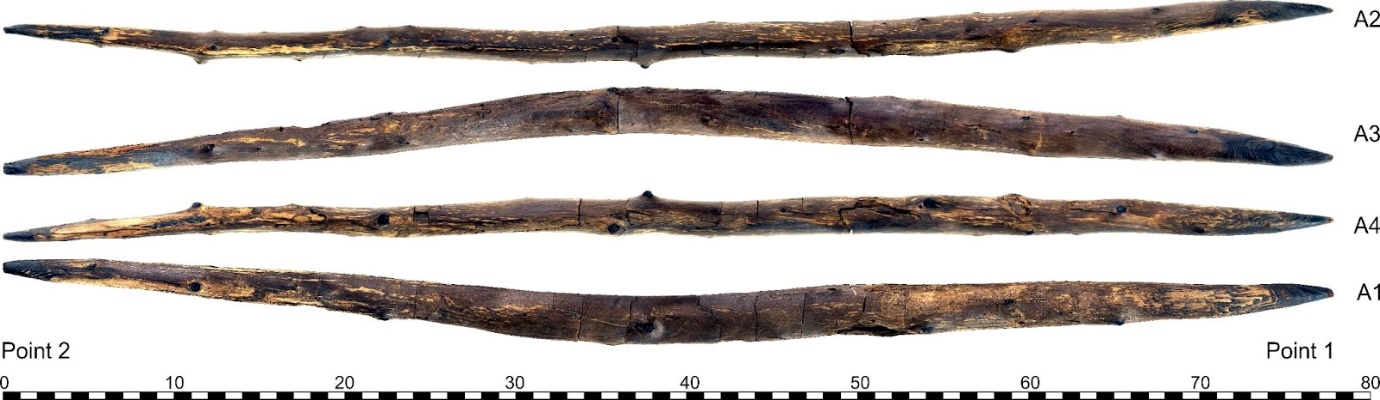


**Figure S9.** Overview of the double-pointed stick from 2012 showing the blackened points. Note: Contrast and colour saturation are enhanced. The views deviate from those in other figures. Photos K. Schmidt, E. Behrens.

**Figure S10.** ATR-IR results for averaged dark areas (Avg_dark) and light areas (Avg_light) of throwing stick and their respective error bars as standard deviation of absorbances of investigated measurement positions. Additional curve of the neat Kauramin resin that had been used to conserve the artefact. Normalisation to absorbance at 1030 cm^-1^ except for Kauramin.

**Figure S11**. ATR-IR results as in Figure S10, but enlarged wavenumber axis.

**Table S5**. Features on the double-pointed stick connected to Phase 1.

| **Feature** | **View** | **Location** |
| --- | --- | --- |
| annual ring surface | A3–A4  A1–A2  A1 | 18–23 cm  29–33 cm  64–78 cm |
| annual rings - tangential | A1–A2  A1–A4  A2–A3  A3–A4    A2–A3    A1–A2  A1–A4  A2–A3  A3–A4 | 0–8 cm  0–11 cm  0–12 cm  0–12 cm  22–32 cm  24–34 cm  58– 63 cm  64.5–77.2 cm  64.5–77.2 cm  66–77.2 cm  68–77.2 cm |
| knot - flattened | A1–A4  A2–A4  A1–A2  A2–A3  A2–A4  A1–A2  A2–A3  A3–A4 | 2–16 cm  20–23 cm  28 cm  34 cm  44–62 cm  72 cm  75 cm  75 cm |
| knot - smoothed and/or rounded | A1–A4  A3  A3–A4        A1–A4    A3–A4  A1–A4  A2–A3 | 11 cm  12 cm  12 cm  20 cm  22 cm  34 cm  38 cm  53 cm  60 cm  66 cm  75 cm |
| knot - with cut surface | A1 to A4  A1–A2  A2–A3    A1–A4  A1–A2  A2–A3  A1 to A4 | 3–12 cm  23 cm  23 cm  24 cm  46 cm  49 cm  52 cm  60–74 cm |
| knots - with torn surface | A3–A4 | 59.5–60.5cm |
| splinter negative | A2–A3 | 42.5­­­­­­–45 cm  48­­­­­­–49 cm |
| striation - longitudinal (possible cut mark) | A1–A2  A3–A4      A2–A3 | 36–44 cm  39–42 cm  45–47 cm  48–52 cm  48–52 cm  53–56 cm |
| striation - long parallel (scraping mark) | A2–A3  A1–A4  A1  A1–A2  A1–A4  A3–A4    A1–A4 | 22–27.5 cm  22–50 cm  23–50 cm  29–33 cm  34–35 cm  40–55 cm  50–77.2 cm  54–64 cm |
| striation - short oblique (cut mark) | A3–A4  A2–A3  A3–A4    A2–A3  A1–A4  A1  A2–A3  A1–A2  A3–A4 | 10–11.5 cm  12–13 cm  18–20 cm  36 cm  37 cm  39–45 cm  41–44 cm  43–44 cm  49 cm  58 cm  64–66.5 cm |
| striation - with stop mark | A1–A2 | 29–33 cm |
| stop mark | A2–A3  A1–A2  A2–A3  A3  A3–A4 | 3 cm  29.5–34cm  43 cm  45–46.5 cm  66–69 cm |
| surface facets | A2–A3  A1–A4 | 0–35 cm  69–77.2 cm |
| tool mark with side features/signatures | A2–A3    A3–A4 | 9–11 cm  22–27.5 cm  55–62 cm |

**Phase 2**

**Table S6.** Features on the double-pointed stick connected to Phase 2.

| **Feature** | **View** | **Location** |
| --- | --- | --- |
| Discolouration | A2–A3 | 40–56 cm |
| Polish on knot | A1–A4 | 37 cm |

**Phase 3**


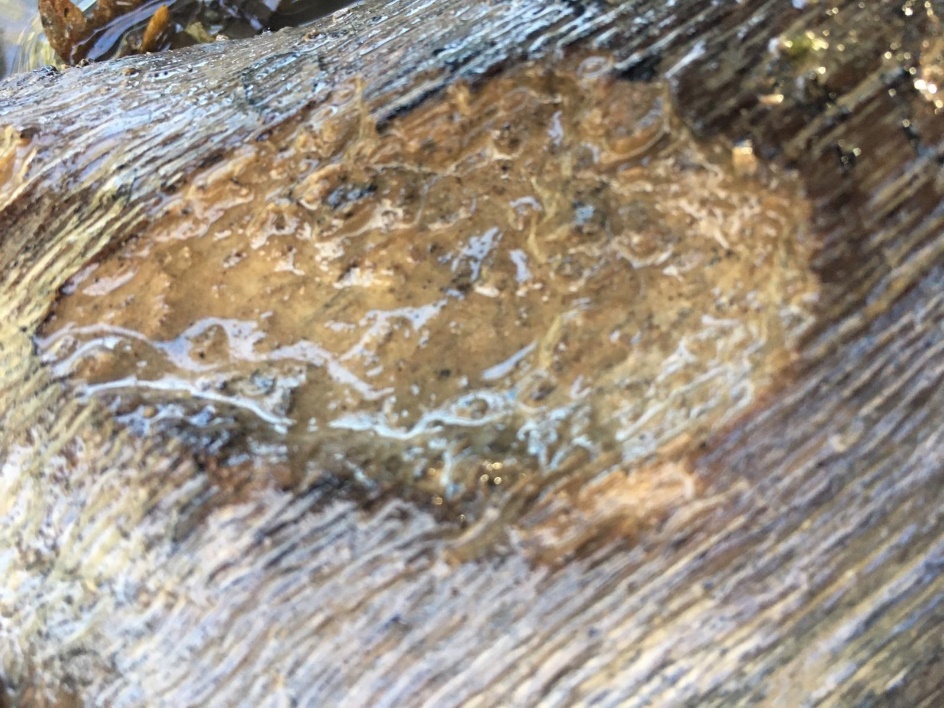


**Figure S12.** Damage observed from a modern wet wood context where there is significant trampling by humans. Photo: A. Milks.

**
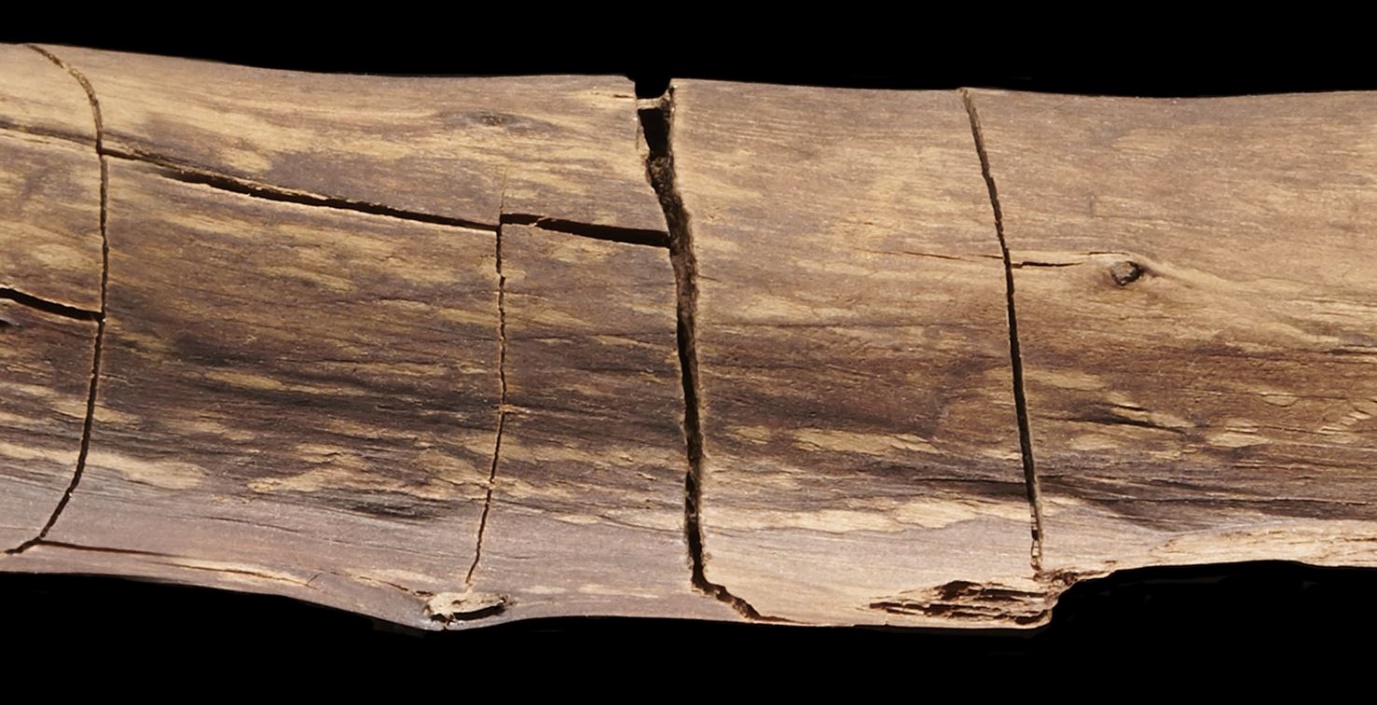
**

**Figure S13.** Detail of the transverse break (View A2­­­­­­–A3, 50 cm) on the double-pointed stick, showing further longitudinal cracking emanating from the break, and additional transverse cracks. Photo: V. Minkus.

.
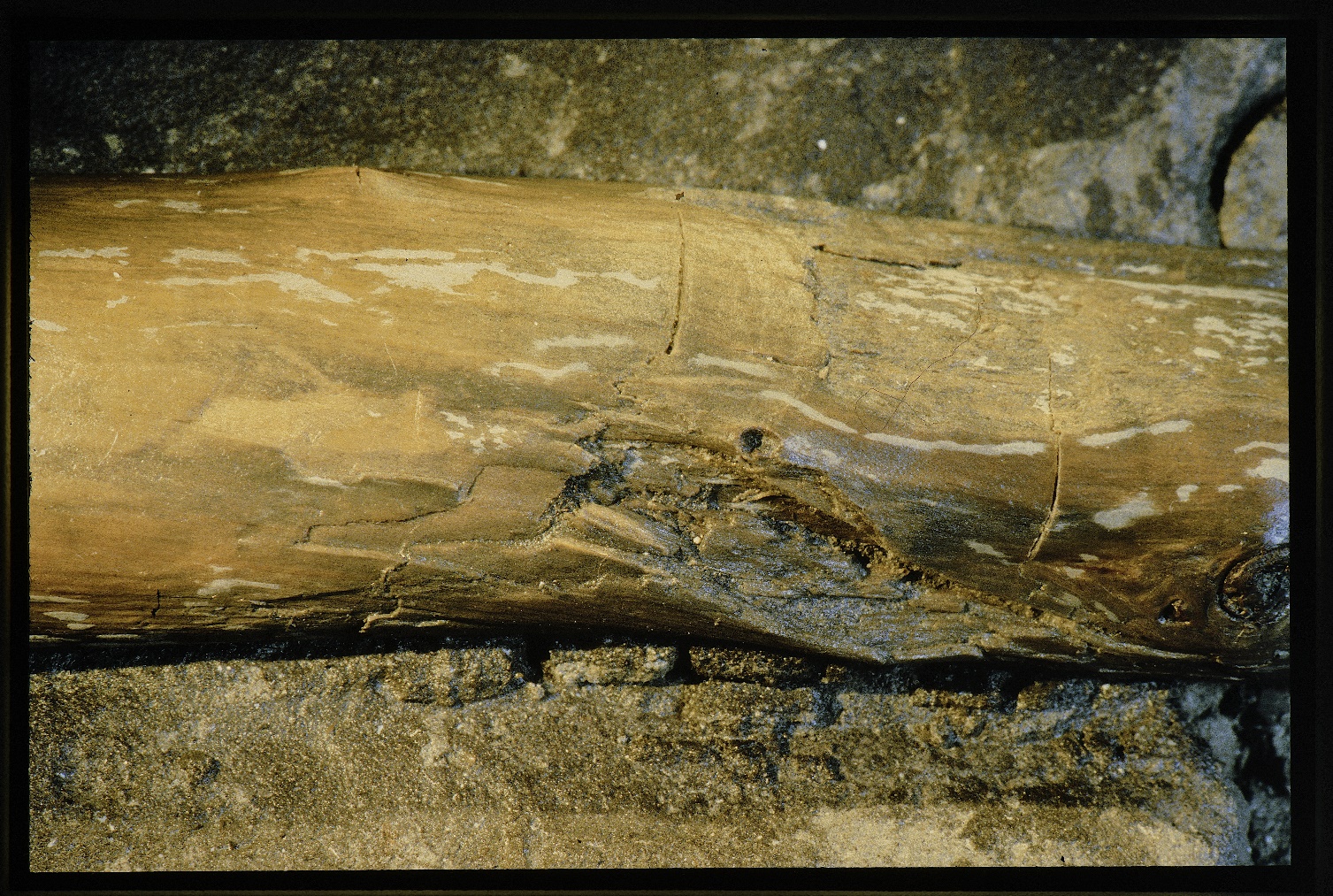


**Figure S14.** Close-up of the area where the present-day break is located (ca. 50 cm, View A1­­­­­­–A4), showing the current fracturing into two fragments occurred after excavation. Photo: Fuchs.


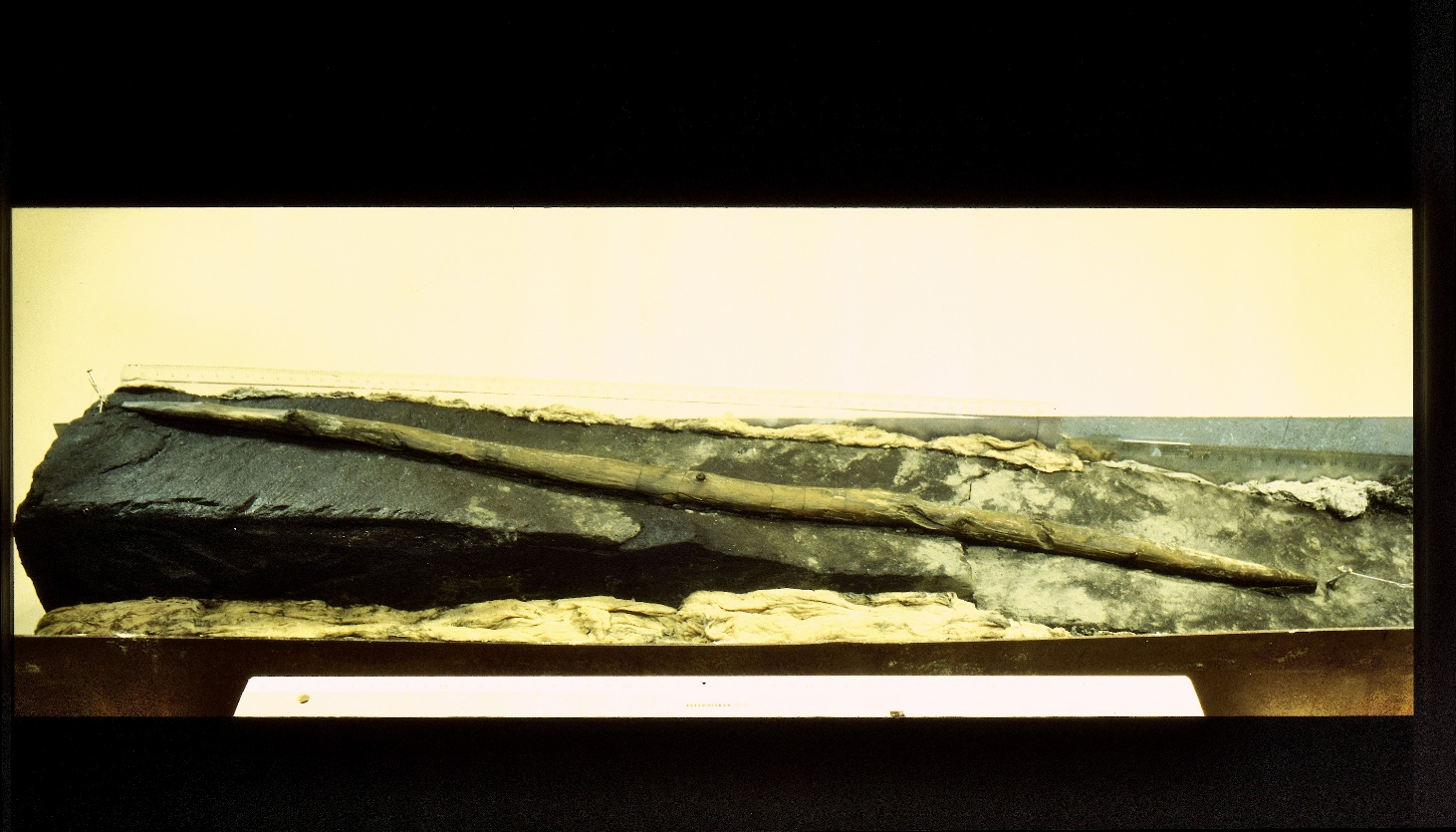


**Figure S15.** Photograph of the double-pointed stick in the excavation block, showing that the artefact was fragmented after excavation. Photo: Fuchs.


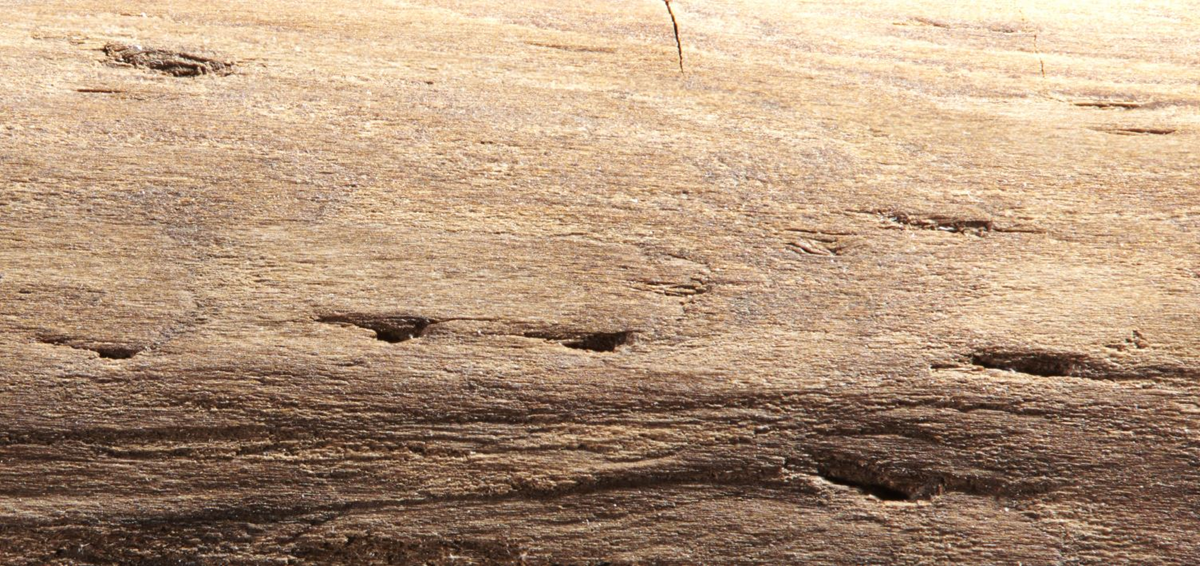


**Figure S16.** Detail of the taphonomic puncture marks on View A1­­­­­­–A4 at 68­­­­­­–70 cm. Photo: V. Minkus.


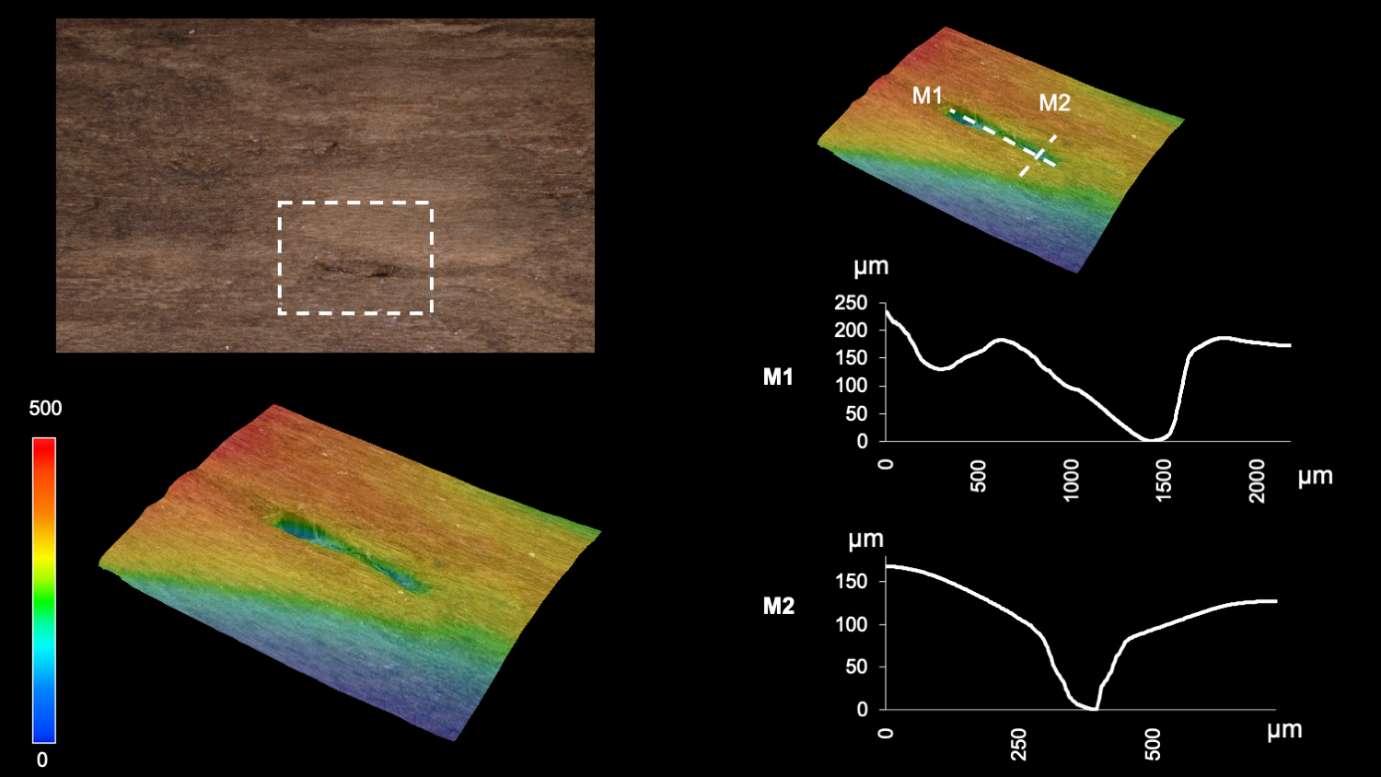


**Figure S17**. 3D microscopy of one of the taphonomic punctures caused by root damage on view A1 at 68 cm. Image: T. Koddenberg.

**Table S7**. Features on the double-pointed stick connected to Phase 3.

| **Feature** | **View** | **Location** |
| --- | --- | --- |
| compression - localised | A1–A2    A2–A3 | 29–33 cm  46–50 cm  58–69 cm |
| crack (longitudinal) | A1 to A4 | multiple |
| crack (transverse) | A1 to A4 | multiple |
| flecking | A2–A3  A2  A1–A4  A3–A4  A1–A4 | 12–53 cm  12–61 cm  13–31 cm  24–29 cm  42–44 cm |
| mineralisation | A1–A2 | 75.5–76 cm |
| puncture | A1–A4    A1–A2 | 53–56 cm  66–77 cm  68–77 cm |
| trampling damage | A1–A4 | 12.5–14.5 cm  15–18.5 cm  58–61 cm  62–65 cm |
| surface damage | A1–A2  A2–A3  A3–A4  A1–A4 | multiple  localised  multiple  multiple |

**Phase 4**


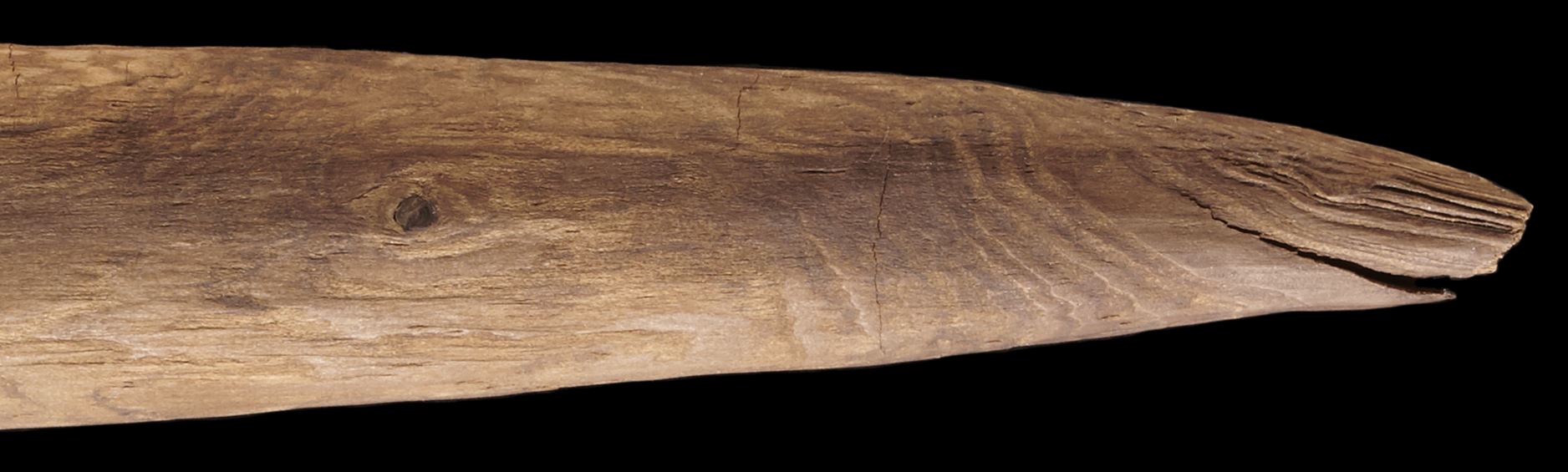


**Figure S18.** Detail image of Point 1 showing the recent split at the tip and annual ring areas. Photo: V. Minkus.

**Table S8**. Features on the double-pointed stick connected to Phase 4.

| **Feature** | **View** | **Location** |
| --- | --- | --- |
| longitudinal crack | A2 | 75.5–77.2 cm |
| transverse cracks (?) | A1 to A4 | multiple |
| post-excavation damage | A1 to A4 | 77.2­­­­­­–77.7 cm |


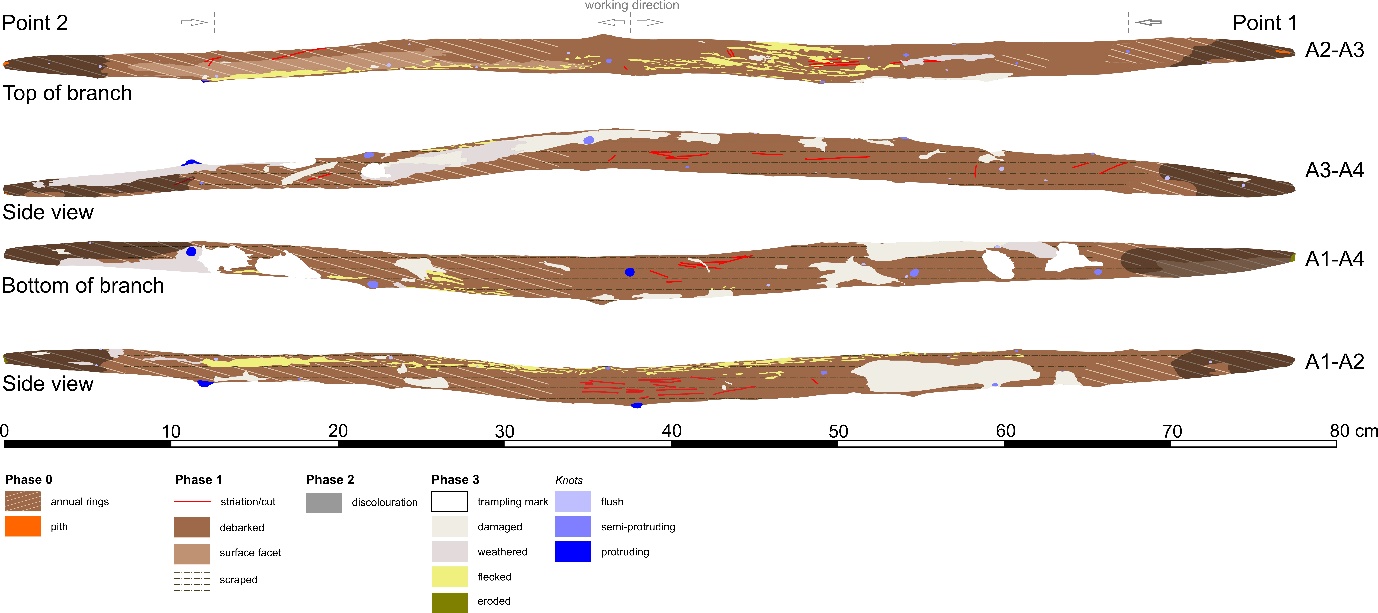


**Figure S19**. Detailed mapping of the double-pointed stick. Drawing: D. Leder.

**Table S9.** Raw morphometric values for each cross-sectional profile. M = magnification; WIS = width at the surface; WIM = width in the middle; WIB = width at the bottom; D = depth; OA = opening angle. WIS, WIB, and D are given in µm, while OA is given in degree. Cm = cut mark; Pm = puncture mark; Tm = Trampling mark.


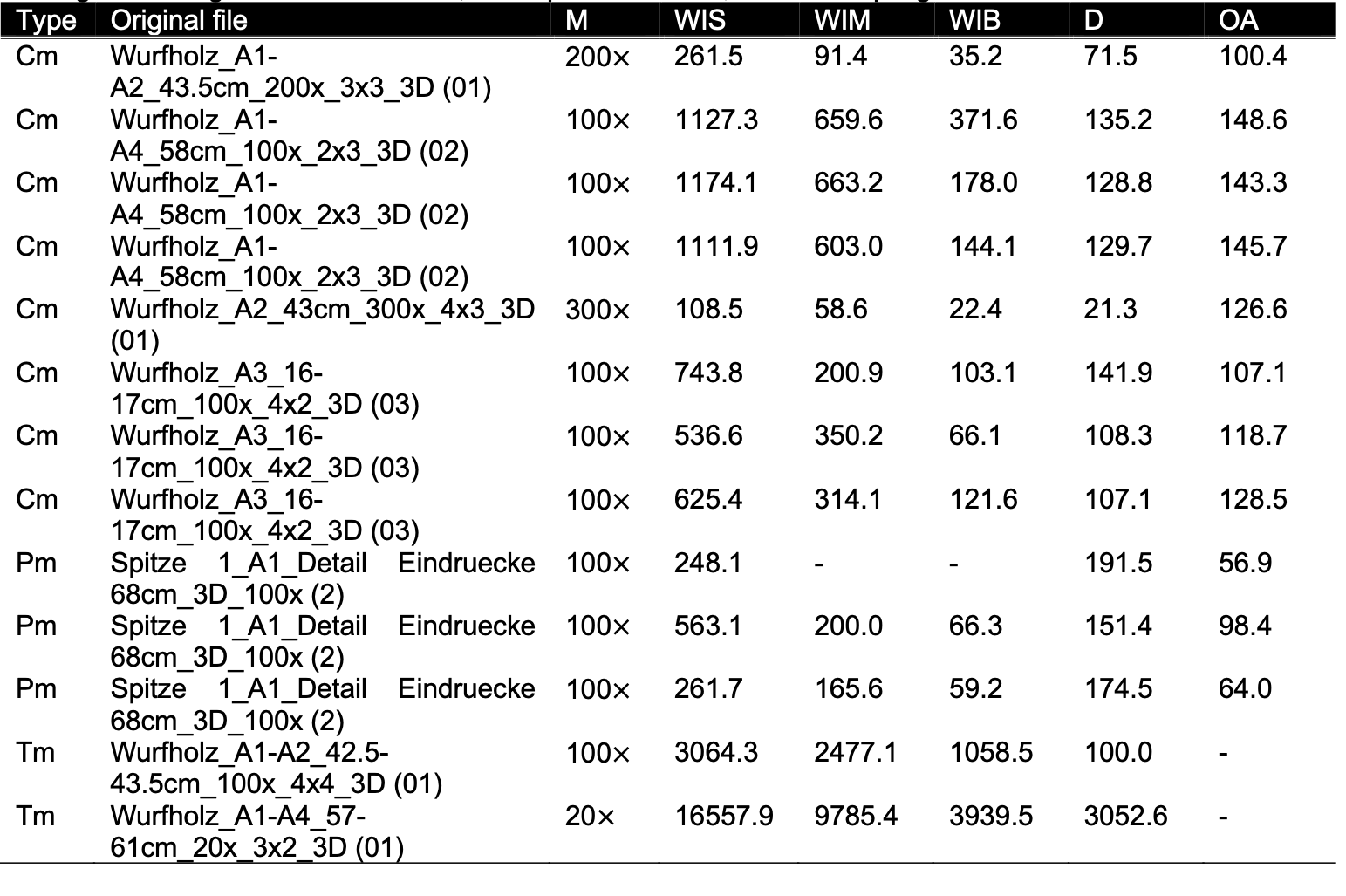


**1.9 Sources for estimates for weapon delivery performance data in Figure 18 (main manuscript)**

Estimates for the performance of prehistoric hunting weapons have been greatly bolstered in recent years through performance experiments involving experienced weapon users, reviews of ethnographic and ethnohistorical literature pertaining to similar weapon systems, and new ethnographic studies aimed at addressing such questions. We acknowledge that our data are limited, as contemporary experiences of Western hobbyists, athletes and archaeologists are unlikely to provide accurately replicate weapon use by people for whom subsistence technologies are socially embedded and learnt from early ages (21,22), and for which communities rely upon these technologies for survival (see also 23). Furthermore, ethnohistorical accounts were likely biased due to colonialist objectives and perspectives, and the extent to which ethnographic analogy is a useful tool for understanding the deep past is a matter of further concern (e.g. 24–26).

With these caveats of the limitations of experimental and ethnographic data in mind, the following sources inform our diagram. For kinetic energy the following studies provided either direct calculations or paired velocity and mass data (13,14,27,28). The following studies provided experimental velocity data (13,14,27–29). Distance estimates are informed by experimental studies (13–15,30) alongside ethnohistorical and ethnographic accounts (31–41) and reviews (14 SI,19,42–44). Energies for wounding prey by body are after Tomka (44).

**References**

1. Bello SM, Galway-Witham J. Bone taphonomy inside and out_ Application of 3-dimensional microscopy, scanning electron microscopy and micro-computed tomography to the study of humanly modified faunal assemblages. Quaternary International. 2019;17. https://doi.org/10.1016/j.quaint.2019.02.035.

2. Bello SM, Parfitt SA, Stringer CB. Quantitative micromorphological analyses of cut marks produced by ancient and modern handaxes. Journal of Archaeological Science. 2009 Sep;36(9):1869–80. https://doi.org/10.1016/j.jas.2009.04.014.

3. Bello SM, De Groote I, Delbarre G. Application of 3-dimensional microscopy and micro-CT scanning to the analysis of Magdalenian portable art on bone and antler. Journal of Archaeological Science. 2013 May;40(5):2464–76. https://doi.org/10.1016/j.jas.2012.12.016.

4. Weiss S, Urdl K, Mayer HA, Zikulnig-Rusch EM, Kandelbauer A. IR spectroscopy: Suitable method for determination of curing degree and crosslinking type in melamine–formaldehyde resins. Journal of applied polymer science. 2019;136(25):47691.

5. Factor A. Char formation in aromatic engineering polymers. In ACS Publications; 1990.

6. Salman A, Tsror L, Pomerantz A, Moreh R, Mordechai S, Huleihel M. FTIR spectroscopy for detection and identification of fungal phytopathogenes. Spectroscopy. 2010;24(3–4):261–7.

7. Sharma RK, Wooten JB, Baliga VL, Lin X, Chan WG, Hajaligol MR. Characterization of chars from pyrolysis of lignin. Fuel. 2004;83(11–12):1469–82.

8. Kwon SM, Jang JH, Lee SH, Park SB, Kim NH. Change of heating value, pH and FT-IR spectra of charcoal at different carbonization temperatures. Journal of the Korean Wood Science and Technology. 2013;41(5):440–6.

9. Smith B. Infrared spectral interpretation: a systematic approach. CRC press; 1999.

10. Smidt E, Tintner J, Klemm S, Scholz U. FT-IR spectral and thermal characterization of ancient charcoals-A tool to support archeological and historical data interpretation. Quaternary International. 2017;457:43–9.

11. Fengel D. Influence of water on the OH valency range in deconvoluted FTIR spectra of cellulose. Holzforschung. 1993;47:103–8. https://doi.org/10.1515/hfsg.1993.47.2.103.

12. Puccioni N. Anthropology and Ethnography of the Peoples of Somalia. In: Zanichelli N, editor. Etnografia E Paletnologia [Internet]. 1936. Available from: https://ehrafworldcultures.yale.edu/document?id=mo04-017.

13. Roach NT, Richmond BG. Clavicle length, throwing performance and the reconstruction of the Homo erectus shoulder. Journal of Human Evolution. 2015 Mar;80:107–13. https://doi.org/10.1016/j.jhevol.2014.09.004.

14. Milks A, Parker D, Pope M. External ballistics of Pleistocene hand-thrown spears: experimental performance data and implications for human evolution. Scientific Reports. 2019 Jan;9(1):820. https://doi.org/10.1038/s41598-018-37904-w.

15. Rieder H. Erprobung der Holzspeere von Schoeningen (400000 Jahre) und Folgerungen daraus. In: Wagner GA, Mania D, editors. Fruhe Menschen in Mittel Europa: Chronologie, Kultur, Umwelt. Aachen: Shaker; 2001. p. 91–8.

16. Kluckhohn C, Hill WW, Kluckhohn LW. Navaho Material Culture [Internet]. Cambridge, MA: Belknap Press of Harvard University Press; 1971. Available from: https://ehrafworldcultures.yale.edu/document?id=nt13-192.

17. Beaglehole E, Beaglehole P. Hopi of the second mesa. In: Memoirs of the American Anthropological Association [Internet]. Menasha, Wisconsin: American Anthropological Association; 1935. p. 65. Available from: https://ehrafworldcultures.yale.edu/document?id=nt09-056

18. Whiting AF. Ethnobotany of the Hopi. In: Bulletin [Internet]. Flagstaff: Northern Arizona Society of Science and Art; 1939. p. viii, 120. Available from: https://ehrafworldcultures.yale.edu/document?id=nt09-035

19. Bordes L. Les bâtons de jet préhistoriques et leurs représentations: Développement d’outils et de méthodes pour la mesure de leurs caractéristiques et l’évaluation de leurs fonctions [Internet] [Master’s Thesis]. 2014.

20. Milks A, Lehmann J, Leder D, Böhner U, Koddenberg T, Sietz M, et al. Wood technology: a Glossary and Code for the analysis of archaeological wood from stone tool cultures [Internet]. OSF Preprints x8m4j, ver. 7 peer-reviewed and recommended by Peer community in Archaeology.; 2022. Available from: https://osf.io/x8m4j/ https://doi.org/1 0.31219/osf.io/x8m4j.

21. Lew-Levy S, Bombjaková D, Milks A, Kiabiya Ntamboudila F, Kline MA, Broesch T. Costly teaching contributes to the acquisition of spear hunting skill among BaYaka forager adolescents. Proceedings of the Royal Society B: Biological Sciences. 2022 May 11;289(1974):20220164. https://doi.org/10.1098/rspb.2022.0164.

22. Lew-Levy S, Reckin R, Lavi N, Cristóbal-Azkarate J, Ellis-Davies K. How Do Hunter-Gatherer Children Learn Subsistence Skills? Human Nature. 2017 Oct;28:367–94. https://doi.org/10.1007/s12110-017-9302-2.

23. Milks A. Skills Shortage A Critical Evaluation of the Use of Human Participants in Early Spear Experiments. EXARC Journal. 2019;2019(2):1–11. https://exarc.net/ark:/88735/10426.

24. French JC. The use of ethnographic data in Neanderthal archaeological research. Hunter Gatherer Research. 2019 Nov;4(1):25–49. https://doi.org/10.3828/hgr.2018.3.

25. Warren G. Is There Such a Thing as Hunter-Gatherer Archaeology? Heritage. 2021 May 14;4(2):794–810. https://doi.org/10.3390/heritage4020044.

26. Gosselain OP. To hell with ethnoarchaeology! Archaeological dialogues. 2016;23(2):215–28. https://doi.org/10.1017/S1380203816000234.

27. Coppe J, Lepers C, Clarenne V, Delaunois E, Pirlot M, Rots V. Ballistic Study Tackles Kinetic Energy Values of Palaeolithic Weaponry. Archaeometry. 2019 Feb;2(4):107–24. https://doi.org/10.1111/arcm.12452.

28. Whittaker JC, Pettigrew DB, Grohsmeyer RJ. Atlatl Dart Velocity: Accurate Measurements and Implications for Paleoindian and Archaic Archaeology. PaleoAmerica. 2017 Apr;3(2):161–81. hps://doi.org/10.1080/20555563.2017.1301133.

29. Milks A, Champion S, Cowper E, Pope M, Carr D. Early spears as thrusting weapons: Isolating force and impact velocities in human performance trials. Journal of Archaeological Science: Reports. 2016 Dec;10:191–203. https://doi.org/10.1016/j.jasrep.2016.09.005.

30. Whittaker JC, Kamp KA. Primitive Weapons and Modern Sport: Atlatl Capabilities, Learning, Gender, and Age. Plains Anthropologist. 2006;51(198):213–21. https://doi.org/10.1179/pan.2006.016.

31. Milks A. A Review of Ethnographic Use of Wooden Spears and Implications for Pleistocene Hominin Hunting. Open Quaternary. 2020 Sep;6(1):79–20.

32. Noetling F. Notes on the hunting sticks (lughrana), spears (perenna), and baskets (tughbrana) of the Tasmanian Aborigines. Papers and Proceedings of the Royal Society of Tasmania. 1911;64–98.

33. Sahle Y, Ahmed S, Dira SJ. Javelin use among Ethiopia’s last indigenous hunters: Variability and further constraintson tip cross-sectional geometry. Journal of Anthropological Archaeology. 2023 Jun 1;70:101505. https://doi.org/10.1016/j.jaa.2023.101505.

34. Spencer WB. Native tribes of the Northern territory of Australia. London: Macmillan and Co.; 1914.

35. Morris J. Relationship between the British and the Tiwi in the vicinity of Port Dundas, Melville Island. Historical Society of the Northern Territory. 1964;

36. Lloyd GT. Thirty-three Years in Tasmania and Victoria: Being the Actual Experience of the Author, Interspersed with Historic Jottings, Narratives, and Counsel to Emigrants . London: Houlsten and Wright; 1862.

37. Roth HL. The aborigines of Tasmania. London: Kegan Paul, Trench, Trübner & Co; 1890.

38. Robinson GA. Friendly mission : the Tasmanian journals and papers of George Augustus Robinson 1829-1834. Plomley NJB, editor. Hobart: Tasmanian Historical Society Research Association; 1966.

39. Giles E. Australia Twice Traversed. Adelaide: Libraries Board of South Australia; 1889.

40. Christison R, Edge-Partington J. 19. Notes on the Weapons of the Dalleburra Tribe, Queensland, Lately Presented to the British Museum by Mr. Robert Christison. Man. 1903;3:37. https://doi.org/10.2307/2839962.

41. Baker SW. Ismailia. Vol. 1. London: Macmillan; 1874.

42. Churchill SE. Weapon Technology, Prey Size Selection, and Hunting Methods in Modern Hunter-Gatherers: Implications for Hunting in the Palaeolithic and Mesolithic. Archeological Papers of the American Anthropological Association. 1993;4(1):11–24. https://doi.org/10.1525/ap3a.1993.4.1.11.

43. Hutchings WK, Brüchert LW. Spearthrower performance: ethnographic and experimental research. Antiquity. 1997;71(274):890–7.

44. Tomka SA. The adoption of the bow and arrow: a model based on experimental performance characteristics. American Antiquity. 2013;78(03):553–69. https://doi.org/10.7183/0002-7316.78.3.553.
