# Supplementary figures and images for: A double-pointed wooden throwing stick from Schöningen, Germany: Results and new insights from a multianalytical study
Source: PLoS One. 2023 Jul 19;18(7):e0287719. doi: 10.1371/journal.pone.0287719 (PMC10355447; doi:10.1371/journal.pone.0287719)

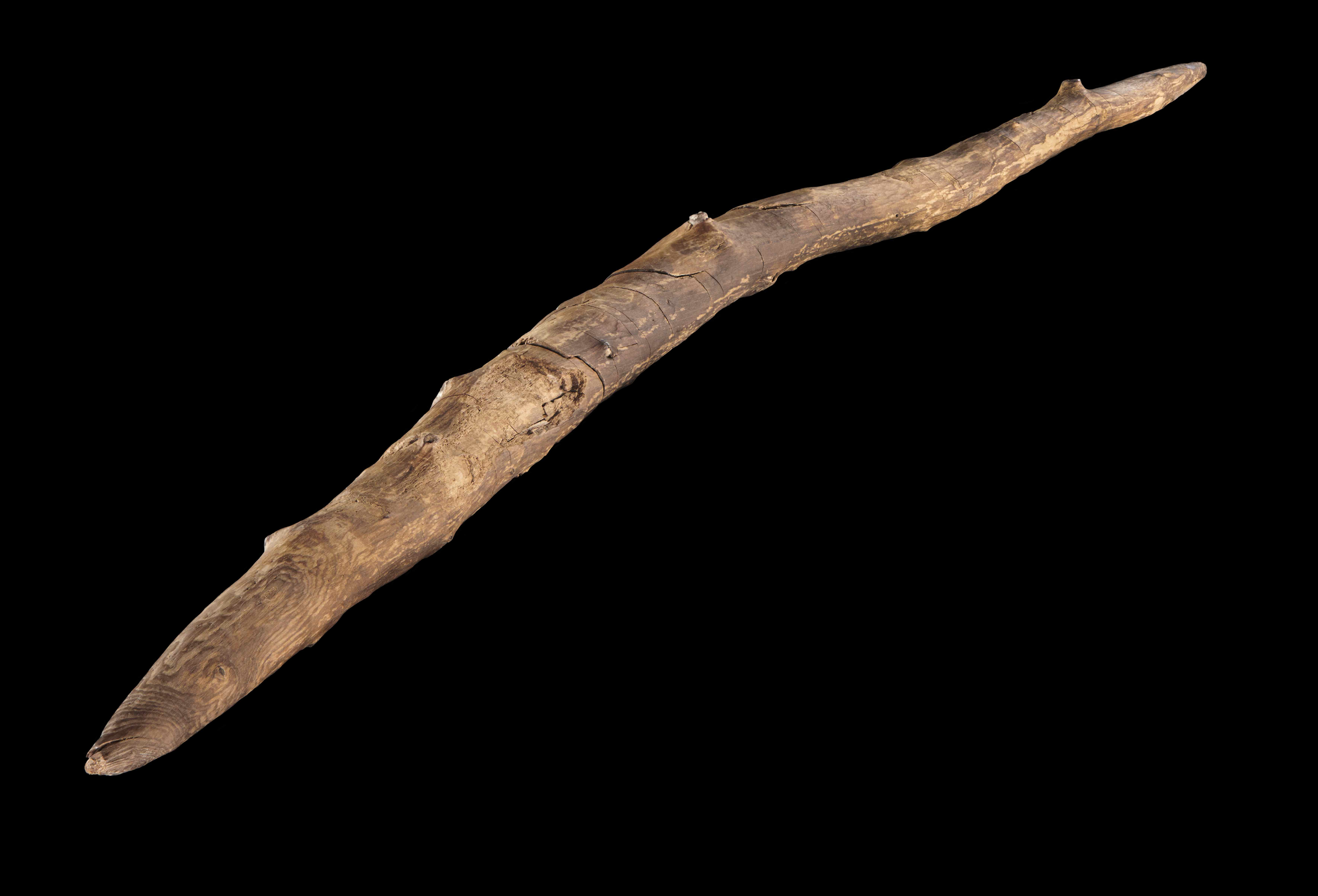

Supplement: S1 Fig — Photo: Volker Minkus. (TIF) [file pone.0287719.s002.tif]
