# Supplementary material for: A double-pointed wooden throwing stick from Schöningen, Germany: Results and new insights from a multianalytical study
Source: PLoS One. 2023 Jul 19;18(7):e0287719. doi: 10.1371/journal.pone.0287719 (PMC10355447; doi:10.1371/journal.pone.0287719)
Supplement: Attachment — Submitted filename: PONE-D-22-35734_reviewer.pdf [file pone.0287719.s007.pdf]

PLOS ONE

Perfectly shaped — A multianalytical study of a double-pointed wooden stick from Schöningen, Germany

--Manuscript Draft--

**Manuscript Number:**	PONE-D-22-35734
**Article Type:**	Research Article
**Full Title:**	Perfectly shaped — A multianalytical study of a double-pointed wooden stick from Schöningen, Germany
**Short Title:**	Perfectly shaped — A multianalytical study of a double-pointed wooden stick from Schöningen, Germany
**Corresponding Author:**	Annemieke Milks, Ph.D.<br>University of Reading Department of Archaeology<br>London, UNITED KINGDOM
**Keywords:**	Palaeolithic, Wood Technologies, Woodworking, Throwing Sticks, Early Weaponry
**Abstract:**	The site of Schöningen (Germany), dated to ca. 300,000 years ago, yielded the earliest large-scale record of humanly-made wooden tools. These include wooden spears and shorter double-pointed sticks, discovered in association with herbivores that were hunted and butchered along a lakeshore. Wooden tools have not been systematically analysed to the same standard as other Palaeolithic technologies, such as lithic or bone tools. Our multianalytical study includes micro-CT scanning, 3-dimensional microscopy, and Fourier transform infrared spectroscopy, supporting a systematic, comprehensive, technological and taphonomic analysis. In illustrating the biography of one of Schöningen's double-pointed sticks, we demonstrate new human behaviours for this time period, including sophisticated woodworking techniques. The hominins selected a spruce branch to craft the weapon. After cutting the branch they seasoned, debarked and shaped the wood into an aerodynamic and ergonomic tool. The weapon was repeatedly used, most likely as a 'hunting stick' thrown rotationally at prey. The stick's completeness suggests it was probably lost during use, and then rapidly buried in mud. Through our detailed analysis we show that Middle Pleistocene humans had a rich awareness of raw material properties, and possessed sophisticated woodworking skills. Signs of repeated use show that this carefully designed and crafted tool had a long use life. An ethnographic review enables us to functionally assess this tool, whilst providing social context pointing to communal hunting, potentially involving children. The Schöningen throwing sticks may have been used to strategically disadvantage larger ungulates but also demonstrate that hominins were equipped to capture fast small prey and avian fauna, a behaviour evidenced at contemporaneous Middle Pleistocene archaeological sites. Throwing sticks are long-distance projectiles, and these hominins could potentially have hunted from as far as 50 metres, similar to the range of bow-and-arrows from sites associated with our own species.
**Order of Authors:**	Annemieke Milks, Ph.D.
	Jens Lehmann
	Dirk Leder, PhD
	Michael Sietz
	Tim Koddenberg, PhD
	Utz Böhner, PhD
	Volker Wachtendorf, PhD
	Thomas Terbgerger, PhD
**Additional Information:**	
**Question**	**Response**
**Financial Disclosure**	T.T. and this project is funded by the Deutsche Forschungsgemeinschaft (DFG,

Powered by Editorial Manager® and ProduXion Manager® from Aries Systems Corporation

Enter a financial disclosure statement that describes the sources of funding for the work included in this submission. Review the submission guidelines for detailed requirements. View published research articles from *PLOS ONE* for specific examples.<br><br>This statement is required for submission and **will appear in the published article** if the submission is accepted. Please make sure it is accurate.<br><br>**Unfunded studies**<br>Enter: *The author(s) received no specific funding for this work.*<br><br>**Funded studies**<br>Enter a statement with the following details:<br>• Initials of the authors who received each award<br>• Grant numbers awarded to each author<br>• The full name of each funder<br>• URL of each funder website<br>• Did the sponsors or funders play any role in the study design, data collection and analysis, decision to publish, or preparation of the manuscript?<br>• **NO** - Include this sentence at the end of your statement: *The funders had no role in study design, data collection and analysis, decision to publish, or preparation of the manuscript.*<br>• **YES** - Specify the role(s) played.<br><br>* typeset	German Research Foundation) – project number 447423357.<br>https://www.dfg.de/<br><br>A.M. Is funded by the British Academy Postdoctoral Fellowship PF21/210027.<br><br>https://www.thebritishacademy.ac.uk/<br><br>The funders had no role in the study design, data collection and analysis, decision to publish, or preparation of the manuscript.
**Competing Interests**<br><br>Use the instructions below to enter a competing interest statement for this submission. On behalf of all authors, disclose any competing interests that could be perceived to bias this work—acknowledging all financial support and any other relevant financial or non-financial competing interests.<br><br>This statement is required for submission and **will appear in the published article** if	The authors have declared that no competing interests exist.

Powered by Editorial Manager® and ProduXion Manager® from Aries Systems Corporation

the submission is accepted. Please make sure it is accurate and that any funding sources listed in your Funding Information later in the submission form are also declared in your Financial Disclosure statement.<br><br>View published research articles from *PLOS ONE* for specific examples.<br><br>**NO authors have competing interests**<br><br>Enter: *The authors have declared that no competing interests exist.*<br><br>**Authors with competing interests**<br><br>Enter competing interest details beginning with this statement:<br><br>*I have read the journal's policy and the authors of this manuscript have the following competing interests: [insert competing interests here]*<br><br>* typeset	
**Ethics Statement**<br><br>Enter an ethics statement for this submission. This statement is required if the study involved:<br><br>• Human participants<br>• Human specimens or tissue<br>• Vertebrate animals or cephalopods<br>• Vertebrate embryos or tissues<br>• Field research<br><br>Write "N/A" if the submission does not require an ethics statement.<br><br>General guidance is provided below. Consult the submission guidelines for detailed instructions. **Make sure that all information entered here is included in the Methods section of the manuscript.**	N/A

**Format for specific study types**<br><br>**Human Subject Research (involving human participants and/or tissue)**<br>• Give the name of the institutional review board or ethics committee that approved the study<br>• Include the approval number and/or a statement indicating approval of this research<br>• Indicate the form of consent obtained (written/oral) or the reason that consent was not obtained (e.g. the data were analyzed anonymously)<br><br>**Animal Research (involving vertebrate animals, embryos or tissues)**<br>• Provide the name of the Institutional Animal Care and Use Committee (IACUC) or other relevant ethics board that reviewed the study protocol, and indicate whether they approved this research or granted a formal waiver of ethical approval<br>• Include an approval number if one was obtained<br>• If the study involved *non-human primates*, add *additional details* about animal welfare and steps taken to ameliorate suffering<br>• If anesthesia, euthanasia, or any kind of animal sacrifice is part of the study, include briefly which substances and/or methods were applied<br><br>**Field Research**<br><br>Include the following details if this study involves the collection of plant, animal, or other materials from a natural setting:<br>• Field permit number<br>• Name of the institution or relevant body that granted permission	
**Data Availability**<br><br>Authors are required to make all data underlying the findings described fully available, without restriction, and from the time of publication. PLOS allows rare exceptions to address legal and ethical concerns. See the PLOS Data Policy and FAQ for detailed information.	Yes - all data are fully available without restriction

Powered by Editorial Manager® and ProduXion Manager® from Aries Systems Corporation

A Data Availability Statement describing where the data can be found is required at submission. Your answers to this question constitute the Data Availability Statement and **will be published in the article**, if accepted.<br><br>**Important:** Stating 'data available on request from the author' is not sufficient. If your data are only available upon request, select 'No' for the first question and explain your exceptional situation in the text box.<br><br>Do the authors confirm that all data underlying the findings described in their manuscript are fully available without restriction?	
**Describe where the data may be found in full sentences. If you are copying our sample text, replace any instances of XXX with the appropriate details.**<br><br>• If the data are **held or will be held in a public repository**, include URLs, accession numbers or DOIs. If this information will only be available after acceptance, indicate this by ticking the box below. For example: *All XXX files are available from the XXX database (accession number(s) XXX, XXX.).*<br>• If the data are all contained **within the manuscript and/or Supporting Information files**, enter the following: *All relevant data are within the manuscript and its Supporting Information files.*<br>• If neither of these applies but you are able to provide **details of access elsewhere**, with or without limitations, please do so. For example:<br><br>*Data cannot be shared publicly because of [XXX]. Data are available from the XXX Institutional Data Access / Ethics Committee (contact via XXX) for researchers who meet the criteria for access to confidential data.*<br><br>*The data underlying the results presented in the study are available from (include the name of the third party*	All datasets are submitted along with the manuscript, and can either be downloadable with the publication, or alternatively we would be happy to make them available as a collection on figshare. The 3D model is submitted via URL dropbox links and upon publication will be publicly available on the NLD department's Sketchfab account.

and contact information or URL).<br>• This text is appropriate if the data are owned by a third party and authors do not have permission to share the data.<br><br>* typeset	
Additional data availability information:	

Manuscript

Click here to access/download;Manuscript;DPS To Submit.docx

Title Perfectly shaped — A multianalytical study of a double-pointed wooden stick from Schöningen, Germany

Authors Annemieke Milks[1], Jens Lehmann[2], Dirk Leder[2,] Michael Sietz[3], Tim Koddenberg[4], Utz Böhner[5]**,** Volker Wachtendorf[6], Thomas Terberger[2,7]

[1] Department of Archaeology, University of Reading.[2] Department of Hunter-Gatherer Archaeology, Niedersächsisches Landesamt für Denkmalpflege (Lower Saxony State Office for Cultural Heritage). [3] Archaeological Conservation Department, Niedersächsisches Landesamt für Denkmalpflege (Lower Saxony State Office for Cultural Heritage). [4] Wood Biology and Wood Products, Faculty of Forest Sciences and Forest Ecology, University of Göttingen. [5] Inventory and Heritage Atlas, Niedersächsisches Landesamt für Denkmalpflege (Lower Saxony State Office for Cultural Heritage). [6] Bundesanstalt für Materialforschung und -prüfung (BAM), Unter den Eichen 87, 12205 Berlin, German [7] Department of Prehistoric Archaeology, University of Göttingen.

Keywords: Palaeolithic, Wood Technologies, Woodworking, Throwing Sticks, Weapons, hunting

Abstract The site of Schöningen (Germany), dated to ca. 300,000 years ago, yielded the earliest large-scale record of humanly-made wooden tools. These include wooden spears and shorter double-pointed sticks, discovered in association with herbivores that were hunted and butchered along a lakeshore. Wooden tools have not been systematically analysed to the same standard as other Palaeolithic technologies, such as lithic or bone tools. Our multianalytical study includes micro-CT scanning, 3-dimensional microscopy, and Fourier transform infrared spectroscopy, supporting a systematic, comprehensive, technological and taphonomic analysis. In illustrating the biography of one of Schöningen's double-pointed sticks, we demonstrate new human behaviours for this time period, including sophisticated woodworking techniques. The hominins selected a spruce branch to craft the weapon. After cutting the branch they seasoned, debarked and shaped the wood into an aerodynamic and ergonomic tool. The weapon was repeatedly used, most likely as a 'hunting stick' thrown rotationally at prey. The stick's completeness suggests it was probably lost during use, and then rapidly buried in mud. Through our detailed analysis we show that Middle Pleistocene humans had a rich awareness of raw material properties, and possessed sophisticated woodworking skills. Signs of repeated use show that this carefully designed and crafted tool had a long use life. An ethnographic review enables us to functionally assess this tool, whilst providing social context pointing to communal hunting, potentially involving children. The Schöningen throwing sticks may have been used to strategically disadvantage larger ungulates but also demonstrate that hominins were equipped to capture fast small prey and avian fauna, a behaviour evidenced at contemporaneous Middle Pleistocene archaeological sites. Throwing sticks are long-distance projectiles, and these hominins could potentially have hunted from as far as 50 metres, similar to the range of bow-and-arrows from sites associated with our own species.

1. Introduction

Of all the raw materials that Pleistocene humans made use of for material culture, wood is one the most under-represented in the archaeological record. However, discoveries of wooden tools have revolutionised our perspectives on Palaeolithic technologies and hominin behaviours. Some significant discoveries include wooden spears and digging sticks that together altered perspectives on Middle Pleistocene hominin diets and hunting (1–3), Late Glacial wooden arrows proving bow-and-arrow hunting during the Palaeolithic (4,5) and the Late Glacial Shigir idol, which is the earliest monumental sculpture (6). In spite of this significance and perhaps due to their rarity, methods to analyse wooden finds that do survive are underdeveloped, and particularly so in relation to processes of manufacture and use. In this paper, we bring to the forefront new opportunities to analyse and interpret Pleistocene wooden tools through a case study of the first of the wooden tools discovered at Schöningen 13 II-4, a Middle Pleistocene site in Germany dated to ca. 300,000 years ago.

The subject of our study is a relatively short double-pointed wooden stick which, like most of the other wooden tools from the context at Schöningen known as the ‘Spear Horizon’, was shaped from spruce (*Picea* sp.). In contrast to the famous Schöningen spears this complete stick is short, measuring under a metre. Although it was the first significant find, it remained an anomaly until a second stick of similar size and shape was recently discovered in new excavations of the ‘Spear Horizon South’ (7). Researchers interpreted both of the shorter double-pointed sticks as ‘throwing sticks’ (or ‘hunting sticks’) designed to be thrown rotationally at prey. This functional assessment was based on morphological comparison with ethnographic throwing sticks and supported by the suggestion that surface damage on the newly discovered stick was consistent with impact (2,7). Although selective features including growth of the wood, selected working traces, measurements, and morphologies for the more important Schöningen 13 II-4 wooden artefacts are published (2,7,8) a detailed macro- and micro-analysis of anatomical features of the raw material, surface traces, fractures, and morphometrics has not been undertaken until now.

Nearly three decades have passed since the initial discoveries of the exceptionally preserved wood assemblage at Schöningen, yet we remain at something of an impasse in understanding the role of wood technologies in Lower and Middle Palaeolithic hominin techno-complexes. Some relevant key sites with wood finds have in the interim period been discovered, and their analytical approaches have advanced our understanding of later Middle Pleistocene Neanderthal wood technologies. Few researchers have addressed taphonomic effects on

Pleistocene wood artefacts (but see 9). In the last five years there has been methodological progress in the application of experimentation, macro- and micro-traceology, and chemical analyses to understand manufacturing and use traces and growth features of prehistoric wood artefacts (e.g. 1,10–12). Building on this recent work and that of wood specialists working to elaborate on the *chaîne opératoire* of later prehistoric wood (e.g. 13,14), we present a case study that explores in detail the cultural biography of one of the two double-pointed sticks from Schöningen, inclusive of taphonomic and post-excavation alterations. We do so using multiple analytical techniques that enable detailed surface trace and chemical analyses. These techniques include the application of macro- and microscopic approaches such as 3-dimensional surface microscopy, micro-computed tomography (micro-CT) scans, 3D models, Attenuated total reflection Fourier-transform infrared spectroscopy (ATR-FTIR), and macro-photography. We present the results within a framework that follows the biography of the tool from raw material selection through to excavation and conservation, elaborating on detailed traces attributed to each of its phases over the last ca. 300,000 years.

1.1 Background on the site and find

In the autumn of 1994, Hartmut Thieme discovered the Palaeolithic site of Schöningen 13 II in a lignite mine south of Helmstedt (Germany). Subsequent excavations of sediment sequence 4 (Schöningen 13 II-4) yielded faunal remains including butchered animals and flint artefacts (2). The first discovery of a wooden implement was the double-pointed stick which is the subject of this paper (**Error! Reference source not found.**). During the following years Thieme and the excavation team uncovered further worked wooden objects including the famous Schöningen spears, and interpreted the site as a single mass hunting event (2,15).

Figure 1. Overview photo of the double-pointed stick. Photos: V. Minkus

The wooden artefacts from 13 II-4 are located within layers that transition between organic peat and marl sediments (4b-4b/c) (8,16). The waterlogged contexts led to the exceptional organic preservation including macro- and micro-fauna, and macro- and micro-botanical remains that together demonstrate that Schöningen 13 II-4 was a lakeshore setting (17,18). Thermoluminescence dating suggests the site formed between ca. 337-300 ka BP corresponding to the end of interglacial Marine Isotope Stage (MIS) 9, in a context of deteriorating climatic conditions (19). Butchered animal remains are primarily from horse (*Equus mosbachensis*), but also include species such as red deer (*Cervus elaphus*), and bovids (20). The Schöningen hominins also used expedient bone tools, including exploitation of the humerus of a saber-toothed cat (*Homothereum latidens*) as a lithic retoucher (21,22).

Lithic tools at the site are primarily assigned to a Lower Palaeolithic technology and include retouched and unretouched flakes from frost-shattered flint blanks, some of which show use-wear consistent with woodworking taking place at the site (23,24). Subsequent sedimentary pressure and faulting resulted in some deformation of nearly all the wooden finds, which were likely susceptible to alteration due to their waterlogged condition (8). Evidence that Eurasian Middle Pleistocene hominins controlled fire comes from hearths at sites dated to MIS 13 and MIS 11 (25–28). While it was initially thought that Schöningen had evidence of both hearths and burnt wooden implements, subsequent analyses provided support for neither (29). On the other hand, in that study only a single piece of wood from an earlier site (Schöningen 12 B) was subjected to analysis, and the question of use of fire to shape and/or alter the wood at Schöningen remains an open one.

Today the wooden artefacts from Schöningen 13 II-4 consist of complete and fragmented tools including at least ten spears, manufactured from spruce (n=9; *Picea* sp.) and pine (n=1; *Pinus sylvestris*) (8). The complete spears are double-pointed, measuring between ca. 184 cm and ca. 253 cm in length and between ca. 2.9 and ca. 4.7 cm in maximum diameter. Spear II has been experimentally tested, with results demonstrating its functionality as both a thrusting and hand-thrown spear (30–33). Spear VI is shaped differently to the other spears, as whilst it is also double pointed it has a significant natural kink, making it unlikely to have been a projectile (8). In addition to the larger spears there are two shorter double-pointed sticks shaped from spruce, and hundreds of smaller wet wood fragments that are currently undergoing analysis.

The double-pointed stick analysed in this paper is now in two fragments but was originally fitted together *in situ* (Figure 2). It was the only wooden artefact in Sector 2 (square meter x 684/ y 31), located several metres away from the nearest spears (

Figure 3) and ca. 120 m from the second stick (square meter x 772/ y -49) (16). After discovery the piece was conserved using Kauramin 800™, a mixture of oligomeric and monomeric forms of melamine and formaldehyde (34), and is currently on display at the Forschungsmuseum Schöningen. Both of the short double-pointed tools, measuring under 1 metre, have various functional interpretations, including as ‘throwing sticks’, shorter thrusting or stabbing weapons, digging sticks, bark peelers, or children’s spears (2,7,8).

Figure 2. Excavation photograph of the double-pointed stick. Photo by Peter Pfarr.

Figure 3. Location of the find in the wider site context. Image by U. Böhner.

2. Materials and Methods

2.1. Structural, morphometric and trace analysis

The non-destructive analyses of manufacturing traces comprised a combination of methods developed on the basis of existing approaches to wood and bone tools, including macro- and micro-analyses. The length measurement was taken as a straight line between two points in View A1. Sedimentary pressure altered the cross-sectional shape along most of the length of the tool. To account for this, diameter measurements were taken using callipers in two directions (A1-A3 and A2-A4) and are reported as averages. Morphological descriptions follow Bordes (35). The artefact was investigated on a macro-scale using variable light sources with the naked and a loupe. Macro photographs (labelled in the manuscript as taken by Volker Minkus) were taken using a Nikon D850 with 50mm Sigma-ART and Nikon 105mm lenses (resolution of 45,7 megapixels). Markers located beside the artefact helped to find the correct positioning. Subsequent development of the images was undertaken using Helicon Focus, Capture One and Adobe Photoshop software.

A high-resolution 3D model was created by GOM Metrology to examine and present surface features and overall tool morphology. Micro-traces were investigated and documented with the use of a stereo microscope (LEICA M80). Micro-computed tomography (micro-CT) scanning was performed by Waygate Technologies using a phoenix V|tome|xm enabling the investigation of internal growth and taphonomic features, as well as elucidating the manufacturing process. Topographic surface imaging of selected surface features was accomplished using the 3D reflected light microscopy Keyence VHX-5000. Based on such topographic 3D images, morphometric analyses (e.g., profile lines) of selected surface features were also performed enabling further evaluation of the causes of features such as striations. Detailed information about the methods used for the 3D surface analyses, micro-CT scanning and 3D model is found in SI Sections 1.1, 1.2 and 1.4 respectively. All traces from phases 1 through 4 were mapped onto a digital drawing of the double-pointed stick.

2.2 Residue analysis

Apart from structural imaging, chemical analysis of a selected dark patch were performed using non-destructive Attenuated total reflection Fourier-transform infrared spectroscopy

(ATR-FTIR) to understand whether these areas are residues could have been formed by mould or by deliberate charring. FTIR was selected based on previous application of this wood and wood components (36–40) and charring (41–43). We selected View A2 on Point 1, concentrating on seven darker areas near the point, and three visually lighter coloured areas near the centre of the artefact. Detailed information about the materials and methods for the residue analysis is in SI Section 1.3.

2.3 Wood species identification

The identification of wood species follows established botanical methods. Tree ring sequences and analysis were reconstructed with the use of micro-cuts and micro-CT scans. The micro-cuts were documented with a digital microscope (LEICA M125c reflected light microscope, planapochromatic objectives 0.63x and 1.6x), MC170 HD CAMERA, CLS 150 LED ring light and LEICA-LAS software v4. 12.0. These approaches enable an estimation of the minimum age of the branch and thus tree, the identification of growth characteristics, and other features such as compression wood. In addition, natural material and standard reference texts (e.g. (44) were consulted for interpreting natural features.

2.4 Establishment of views

The object was analysed on four planes, labelled Views A1 through A4. View 1 is known as A1 (for German designation 'Ansicht 1') and is defined for the larger Schöningen wood objects by the surface that was uppermost in excavation. Because it is a 3D object in the round and the views as originally defined do not correspond to the way the wood grew, we often refer to the surface between two views (e.g. View A1-A2). The two points are designated as Point 1 and Point 2 (**Error! Reference source not found.**). In order to remain consistent with previous research and reports, this study follows the original measurement system, with 0 cm starting from Point 2.

2.5 *Chaîne opératoire* approach and nomenclature

This paper uses a new standardised reference glossary with defined terms organised within a *chaîne opératoire* framework, which we have recently published (45). The reader is referred to this glossary for definitions of terms used throughout the paper (e.g. branch whorl, tangential annual rings, tool facet, mineralisation). The cultural biography of the artefact is explored through five phases from raw material sourcing through to excavation (Table S3). Methods to evaluate potential use traces (Phase 2) on Pleistocene wood tools, and to reliably distinguish them from manufacturing and taphonomic damage are underdeveloped (but see 46). To

facilitate analysis of use traces and fractures we used an experimental reference collection of spruce spear replicas used for thrusting and throwing (30,31,33). To assess taphonomic factors we used existing publications on natural wet wood (9,47) alongside new observations.

2.6 Ethnographic analogy

To better understand characteristics and contexts of 'hunting sticks' (also called 'throwing sticks') we conducted a systematic review of ethnographic literature using keywords to search in the eHRAF database. Results were coded for location, use, prey type and morphometrics (see SI Section 1.6 for detailed methods). The full results and sources are available as an accompanying dataset.

3. Results

3.1 Phase 0: Raw Material

The double-pointed stick was manufactured using spruce (*Picea* sp.) (2). The use of spruce is in keeping with the wider sample of worked wood from the find horizon, although ongoing analysis of the material shows that hominins also exploited pine (*Pinus sylvestris*) and larch (*Larix*). Natural background wood includes willow (*Salix*) and poplar (*Populus*). According to palynological analyses, spruce did not belong to the natural background vegetation at the site and the raw material was introduced by hominins (17,18).

The blank (i.e. the piece of wood prior to preparation and shaping) (e.g. 48) selected for the tool was a branch. We determined this from the position, number, and formation of branches on the artefact (Figure 4). Branch whorls are an arrangement of three or more branches at the same height around the trunk of conifer trees (Figure S4), and they appear star-shaped in cross-section (45). In contrast, branch shoots emerging from parent branches have a different growth pattern, and tend to be stronger and more numerous along the parent branch's top and sides due to exposure to light. Morphologically, spruce branches typically have a natural upward curvature (Figure S5). The stick shows a number of characteristics consistent with the formation of a branch rather than a trunk. First, the absence of seams and of clear, organised and symmetrical branch whorls argues against it being a trunk. Second, the wood displays a curvature which is visible best on View A3-A4 (**Error! Reference source not found.**). We estimated 47 knots encompassing both previously living and dead limbs along the length of the tool, and these are asymmetrically placed. The vast majority of knots are located on View A2-A3, which can also be explained by the deeper working of the wood and the associated cutting of older, already overgrown knots, followed by a smaller number of knots on views A1-

A2 and A3-A4, alongside a dearth of branches on view A1-A4 (Table 1). The asymmetrical placement of knots along the view A2-A3 corresponding to the curvature suggests that this was the top of a naturally curving branch.

Figure 4. Schematic reconstruction of the double-pointed stick in relation to the raw material selection. Image by D. Leder.

Table 1. Location of knots on the four views.

View	Knots
A1-A2	10
A2-A3	18
A3-A4	10
A4-A1	9

Additional growth characteristics are evidenced through the tree ring analyses. Tree rings are wider on one side of the blank in relation to the central pith, and the growth may also be eccentric, with the pith outside of the central axis of the cross-section (Figure 5). In the wood of stems and branches, eccentric growth can be caused by the formation of reaction wood. For gymnosperms eccentricity can occur as a result of the development of compression wood on the underside of branches. The effects of gravity are counteracted by the branch by developing denser and harder wood on the underside, often resulting in an area with wider latewood in that area (44,45,49,50). Thereby, the wide and dense latewood regions in the compression wood appear in distinct bright grayscale intensities in micro-CT images (Figure 5), with more structural uniformity compared with the normal/opposite wood (51). On the double-pointed stick, the eccentricity of the pith is indicated in the cross-sections along the entire length of the piece. This eccentricity is illustrated in a micro-CT slice from the midsection of the tool, where it was only minimally debarked and otherwise undamaged, and thus the off-centre location is not a result of working away more material on selected faces. The double-pointed stick exhibits a natural curvature in the direction of View A1-A4 curving towards View A2-A3. Together the characteristics including an absence of whorls, distribution of knots, presence and location of compression wood in comparison with the curvature, pith eccentricity and curvature demonstrate that the blank selected was a branch.

Figure 5. micro-CT scan slice of an area without surface damage (45 cm). The blue circles in the left image show that the central pith, the smaller blue circle, is eccentric, i.e. not centrally placed in the cross-section. The arrow in the right image points to compression wood (the term for reaction wood in coniferous species) with wider annual rings on the underside of the branch.

A total of 63 tree rings were counted on the artefact at 47 cm, representing a very slow growth rate of ca. 0.2 mm per annum (see also Accompanying Dataset 1). In the centre of the wood the year ring width is subject to strong fluctuations. Annual rings 8, 15, and 17 for example are very narrow, indicating deteriorating growing conditions in the summer months, with little latewood formation (Figure 6b and 6c). In the region of tree ring 10 (Figure 6b, arrow 3), there is compaction of the cell structure, which can be interpreted as the premature latewood formation of the branch during that summer. After a temporary deterioration in growth conditions, possibly due to severe drought, the branch continued to grow again. After a rapid decrease in the width of the tree rings within the first 15 years of growth of the branch, they rarely exceed the 0.2 mm mark (Figure 6c). An extremely small growth in thickness in years 35, 42, 51, 52 and 56 of less than 0.1 mm indicates poor growth conditions, and the narrowest of these, annual ring 56, has a width of only 0.05 mm, consisting of only 2 or 3 cells (Figure 6b, red arrow 5).

Figure 6. a. Taphonomic transverse break surface, from where micro-cuts were taken. b. Composite image of different selections of the cross-section micro-cuts (corresponding to Area 1 on left image. Blue numbers indicate the annual ring. Red arrows highlight features mentioned in the text. c. graph showing measurements (mm) of each annual ring. Images M. Sietz.

In conifers, small tree rings result in higher density wood (44). The narrow growth rings on the artefact reflect poor growth conditions which could result from a response to climate for example, growth at higher altitudes and/or sites with shortened growing seasons or nutrient deficiencies (52). Alternative causes for the continuous decrease in the outer annual ring widths seen on the artefact could be due to it being an older branch (Figure 4) (53), climatic deterioration, infestation of the tree by fungi or insects, or a natural process of the branch dying off. Comparing the growth ring distances in different locations shows a slight narrowing towards Point 2, suggesting that Point 1 was where the branch attached to the trunk (Figure 7). The natural taper was measured by calculating the distance between the pith and annual ring number 40 at two locations (57.2 cm and 20 cm), showing a difference of 2.27 mm. The interpretation of where the branch attached to the trunk is further supported by the fact that dead knots and knot holes are located between 22 cm and 62 cm (Table S4), with 75% located in the Point 1 half of the stick. This indicates that the direction of growth was from Point 1 towards Point 2, with older branch shoots and/or those subjected to more shade most likely to

die off. Finally, the initial direction of the growth of auxiliary branches in the direction of Point 2 can be seen in a longitudinal slice from the micro-CT scan (Figure S6).

Figure 7. A: micro-CT scan of Point 2 (2 cm). B: micro-CT scan of Point 1 (75 cm).

3.2 Phase 1: Manufacture

3.2.1 Morphometric description

Today the stick measures 77.2 cm long, with a maximum diameter of 2.5 cm (for detailed measurements see Accompanying Dataset 2). The location of the maximum diameter is 47.2 cm, or 61% of the length measured from 0 cm (Point 2). Morphologically both points are shaped similarly, and are of similar dimensions. However, although the two ends of both points are of similar size and shape, the lengths of the two tapers differ. The taper towards Point 2 is longer (ca. 45 cm) than that of Point 1 (ca. 22 cm) (Figure 8). There is a very slight natural taper from Point 1 towards Point 2. This natural taper was measured by calculating the distance between the pith and annual ring number 40 at two locations (57.2 cm and 20 cm). The difference in distance between these locations is only 2.27 mm. In contrast, the difference in diameter between the maximum diameter (47 cm) and 20 cm is 7.75 mm. Furthermore, while most of the shaft of Point 1 consists of a debarked surface (Figure 10), along Point 2 (ca. 33.5 cm towards 0 cm) we see clear tangential annual rings on multiple surfaces (see also 3.2.2; Figure 9; Figure 10) reflecting deeper working into the wood in order to taper the shaft. In contrast, Point 1 only has annual ring surfaces measuring less than half the length of those Point 2 (12.7 cm long, from 64.5 cm to 77.2 cm on Views A1-A4 and A1-A2). Therefore, material was deliberately removed from Point 2 over a longer section of the shaft than from Point 1, intentionally creating an asymmetrically shaped tool.

Figure 8. Composite image of the micro-CT slices at regular intervals, illustrating the change in diameter and shaping strategy. Original photo V. Minkus.

Figure 9. A: detail of Point 2 (A2-A3), on the top side of the branch. Note that the tip is broken off just before the pith emerges at the surface. B: detail of Point 1 (A2-A3), with clearly visible annual rings visible due to working, the bevelled shape clearly evidenced, and the pith emerging on the top of the branch. Photos by V. Minkus.

To shape the ends of each point the toolmaker did not follow the natural curvature of the branch, but rather worked away more wood from the top of the branch. The result is that like the larger spears, the softer pith emerges along the side rather than directly at the tips (View A2-A3) (Figure 7; Figure 9; Figure 10). This is visible in the tangential and radial ring areas on both points (Figure 9; Figure 10), and in the tangential cross-sections (Figure 7). The result is a finished tool with a slight curve (visible in **Error! Reference source not found.**, side views

A3-A4, A1-A2). While the current cross-sectional profile of the shaft is slightly elliptical (Figure 8) this is primarily due to natural growth, although there are indications that taphonomic compression also played a minor role (see also 3.4). In contrast, both points are clearly intentionally shaped to create ends with an oval cross-section (Figure 7; Figure 9b).

3.2.2 Manufacturing traces

An absence of significant surface or internal drying cracks indicates the wood was seasoned prior to shaping. Cut wood loses its natural moisture until it is in equilibrium with the surrounding environment, and if freshly cut and debarked wood is allowed to dry too quickly it can develop significant cracks (44). One simple means of accomplishing seasoning would be to leave the bark on for a period of time after removing the branch from the trunk. The tool is fully debarked, with no outer bark, inner bark or cambium (Figure 10). A series of striations oriented obliquely to the shaft have profiles and lengths (ca. 5 mm) consistent with cut marks (Figure 11; Figure 12; Table S5). These cut marks likely facilitated the debarking process. The bark was likely pulled off in strips when relatively fresh, then further removed by scraping. There is a single long curved cut mark, measuring >50 mm in length (View A2-A3 from 14.5 cm to 19.5 cm; Figure S7). Micro-analyses shows this mark has fibre deformation and a profile consistent with an angled cut with a sharp tool edge (Figure 12). In general the 3D microscopy demonstrates morphometric variability (Table S8; Accompanying Dataset 3). Organised groups of longitudinal parallel and sub-parallel striations are likely scraping marks (

Figure 13) to remove remaining bark tissue, and to shape and smooth the tool. In contrast to the points where the wood is worked more deeply, much of the shaft closely follows the course of the debarked surface of the outer annual ring, with these areas characterised by the presence of scraping marks and abraded areas. A small number of tool facets with stop marks and signatures represent deeper carving or possibly planing (e.g.

Figure *14*; Table S5). The stop marks show the working direction; in the centre of the shaft the working direction was towards the points, while at both points the working direction was towards the centre (Figure 10). In general on this piece, particularly in comparison with similar observations on the spears, these tool facets and stop marks are faint and were either subsequently deliberately abraded or were smoothed from handling during use.

Figure 10. Schematic simplified mapping all of the traces from phase 1 through phase 4 (see Figure S19 for a more detailed mapping). Drawing D. Leder.

Figure 11. Examples of short, obliquely oriented cut marks. Photo V. Minkus.

Figure 12. 3D microscopy of an example cut mark on View A3. Magnification is 150×. Images T. Koddenberg.

Figure 13. 3D microscopy of an example of an area with long, shallow scraping marks (View A1-A4, 57-61 cm). Images T. Koddenberg.

Figure 14. 3D microscopy of an example of a faint tool facet with stop mark (View A2-A3, 11-15.5 cm). a. Clear tangential annual rings are present on the top left. Intertwined linear brighter and darker patches next to it represent annual rings that were just slightly cut into. Images T. Koddenberg.

The toolmaker created the tapers of both points by carving or planing, in the process creating very long surface facets, which measure over ca. 35 cm on Point 2 (View A2-A3) and ca. 10-12 cm on Point 1 (View A1-A4) (Figure S8, b and d). Surface facet edges are rounded, and were likely smoothed by abrasion, and are visually distinct from edges created through taphonomic compression. We detected only seven clear tool marks in the annual ring areas, attributed to rough carving and/or planing. Finer tool marks are visible on other areas and with a general absence of taphonomic smoothing (see Section 3.4), these areas are interpreted as being deliberately abraded. Additional areas on the shaft have a polished appearance. These areas could be deliberately abraded to create a smooth surface or alternatively they could be use polish from handling. A particular focus was paid to knots (Figure 10). Knots are particularly hard to work, and the toolmaker worked all but two of them down to be flush with the surface (Figure S19). Protruding knots were abraded to round and smooth the surface (Figure 15). The aim of this was likely to improve handling for ergonomic purposes, and to improve aerodynamics by reducing drag.

Figure 15. Examples of worked knots. Arrows on images d and e point to a tool mark. Images T. Koddenberg.

One of the characteristic features of the double-pointed stick is black staining around the perimeter of both points (Figure 10) which was more visually evident prior to conservation treatments (Figure S9). This might be connected to Phase 1 including the potential application of a material such as grease or wax to seal the wood, use of fire to shape the points, or heat treatment of the points over an open fire, with the latter two processes leaving behind soot and charring residues. These traces could also have been left during use (Phase 2), for example from contact with animal fat or blood. Taphonomic factors (Phase 3) can be excluded because in spite of the artefact being embedded fully within the same sedimentary context, the discoloration is restricted to the points. Furthermore, the darker areas on both points also do not faithfully follow the annual ring areas, meaning that this is not from differential staining of exposed heartwood.

The ATR-FTIR measurements of the throwing stick 1-A2 showed wide variation of the spectra obtained and therefore all measurements of light and dark spots respectively were averaged (Figures S10, S11). All spectra of the throwing stick showed being influenced by the embedding agent, Kauramin 800 (differentiating absorption at 909 cm^{-1}). The results showed that IR-spectra of light and dark measurement spots are very similar. The very few differences in absorptions between dark and light areas, (e.g. 1718 cm^{-1}, 1644 cm^{-1}, and 1213 cm^{-1}), are within the width of standard deviation of measurement points and hence are not significant. It was not possible to detect changes on the wood surfaces that resulted from exposure in fire. This is likely due to a number of confounding factors including that wood spectra are a mixture of the main components lignin and (hemi-) cellulose, and growth rings result in varying proportions of these components and in a resulting modulation of the spectra. Furthermore, the embedded resin varies in thickness which superimposed a background on all spectra of the wood. The unexposed wood component lignin is aromatic, and the embedded Kauramin resin adds aromaticity via its triazine ring (Weiss 2019). Thus the charring is not identifiable qualitatively by a new aromaticity peak from graphene (graphene aromaticity peak at for instance 1610 cm.1 [Factor 1990]) but instead only can be identified quantitatively. This also applies to oxidation-induced carbonyl functions, as both lignin (Faix 1991) and Kauramin (Weiss 2019) introduce carbonyl functions. As the changes in IR spectra for the dark zones compared to light ones are quantitative rather than qualitative, the broad local variation of IR spectra on the double-pointed stick did not allow us to differentiate chemically between visually light and dark regions.

3.3 Phase 2: Use, maintenance, and discard

The surface of the stick does not have evidence of impact damage or use fractures. An absence of irregular striations on the points makes it unlikely that the ends were used for activities such as digging, bark peeling or other extractive activities. There is a darker patch with associated polish in the midsection of the shaft of the tool (View A3-A4, ca 40-56 cm) where the stick was probably held. In addition to the potential for use residues on the points (Section 3.2.2) this is the clearest evidence of use on the artefact. If the tool was multifunctionally used as a shorter stabbing weapon, the dark patches on the points could reflect residues such as blood or fat.

3.4 Phase 3: Taphonomy

The artefact was found along the former lakeshore. An absence of insect feeding traces, and of longitudinal cracks caused by desiccation and weathering, indicates that the tool was fully immersed in wet conditions shortly after discard, remaining in a wet context thereafter (9). Bacterial decay consistent with anaerobic conditions is present. With no indication of distribution of taphonomic smoothing consistent with high-energy water conditions, the find was likely embedded within low-energy lakeshore sediments. In striking contrast to most of the surface of the tool that is very well-preserved, there are localised areas of relatively large, sharply demarcated surface damage, particularly on the excavated surface (View A1) and on the sides (Views A2 and A4), but not on the underside (View A3). This type of damage is characterised by sharp edges and flat bases, and their base often follows annual ring surfaces (Figure 16; see also Table S8 and Accompanying Dataset 3). Due to these characteristics, the fractures occurred after a breakdown of the cellulose and lignin that support the cell walls after being underwater for some time, and not as a result of impact when fresh (cf. 7). Such fractures along annual growth rings have also been observed on recent natural wet wood that was walked on by humans (Figure S12). The quantity of this type of damage along the tool also rules out impact damage. Rather, the focus of this surface spalling along the top of the artefact suggests that it can best be attributed to trampling by hoofed animals, a well-known taphonomic effect on bones, including on faunal remains from Schöningen (22).

Figure 16. 3D microscopy of an example of trampling damage. Images T. Koddenberg.

An additional taphonomic signature on the artefact is discolouration, with a brownish-beige patina over all surfaces and features. On View A3 there is also flecking in the form of bright spots (Figure 17, a through c). These form a straight line along the sides (Views A2 and A4)

and are not present on the excavation surface (View A1). We attribute this to fungal attack, specifically white rot which causes discolouration in the form of bleaching (e.g. 54). Due to the positioning and focus of this discolouration, it occurred after discard but prior to total submersion.

Figure 17. Taphonomic and post-excavation features including flecking (a-b), and mineralisation (e). Images T. Koddenberg.

The artefact bears some small (ca. 0.25 cm to 0.56 cm wide) deep, sharp-edged punctures (Figure S16; Table S8; Accompanying Dataset 3). 3D microscopy illustrates that these small punctures are often lenticular in plan and have straight edges (Figure S17). The damage is taphonomic as the wood would have to be very soft and degraded in order for such punctures to form. Such punctures are typical for debarked surfaces of conifer wood from the Spear Horizon and can also be found on some fractured surfaces. In some instances they penetrate through the entirety of a fragment. These damages are attributed to root growth, probably from monocots, once the wood was very degraded.

Taphonomic compression had a small effect on the cross-section of the artefact, resulting in slight deformation in the direction from A1 to A4. This is evidenced by minor deformation of the rays, visible on prepared areas on the fracture surface. Some areas have irregular edges and wavy areas, also a result of minor taphonomic compression (Fig S11). Early documentation of the artefact after excavation shows that small transverse cracks were already present on the surface of View A1. These transverse cracks indicate stresses from sediment pressure, as well as some vertical deformation visible on Views A2 and A4. A small number of short longitudinal cracks are always associated with the transverse cracks, and are also related to taphonomic compression rather than weathering.

3.5 Phase 4: Excavation and post-excavation

The find was discovered during a rescue excavation, and minor damage removed the tip of Point 2 (Figure 9). There are also transverse cracks along the piece (Figure S13), along with minimal damage to Point 1 including a small split that occurred after conservation (Figure S18). The artefact is currently in two fragments with a break located between 48.7 cm and 50.2 cm (Figures S13-S15; see also 3D models). This post-depositional break is characterised by straight edges, with excavation images showing it was excavated and lifted in one piece. Archival records confirm that the complete fracture must have occurred sometime after 2015. However, the initiation of this break may have been a crack from trampling damage, which is present at this location (Views A1-A4 and A1-A2; Figure 10). Blue mineralisation is present in the darker area on Point 1 (Figure 17, d and e).

4. Discussion

4.1 Raw material selection and organisation

Human use of the lakeshore landscape of Schöningen 13 II-4 was during the very end of an interglacial, transitioning into a glacial period with cooling temperatures. In terms of raw material for the wood tools, only trees adapted to less favourable conditions were growing (8,18). Like the spears, the tool was made using slow-grown spruce with extremely narrow tree rings and dense wood. Other Pleistocene wooden spears and wooden spear points were made of yew (*Taxus baccata*) (55–58), which was not available at Schöningen during that period. The spruce wood used by the hominins for the production of weapons was considerably harder than spruce available in the region today. The selection for spruce may be because it was widely available and relatively easy to work in comparison with other available species, but also potentially reflects favourable properties for weapons (59). Analyses of pollen and macro-remains suggests that the spruce wood did not grow at the lakeshore, and may have come from some distance away (17). One region where the wood could have been sourced was the forests of the neighbouring Elm hills, or the Harz mountains approximately 40 km away. In contrast to the spears, the blank selected was not a trunk, but a branch. This key difference means that it is unlikely to be a repurposed broken spear fragment. The hominins may have selected a branch for its natural bend which they then partially reduced through shaping. The use of a branch for this shorter tool could be an economic choice as it takes less time to remove a branch than to fell a tree. A smaller branch would also require less time to work down the material to arrive at the desired length and diameter.

4.2 Manufacture

After an initial rough stage of branch removal, debarking was an important next step in preparing the blank, even for the sections that were not further shaped. Debarking improves handling and flight performance, accelerates the drying process and thus hardening of the tool, and helps prevent insect attack during the use phase (60). Like most of the Schöningen wooden tools, the double-pointed stick bears no traces of inner bark and/or cambium. The systematic and similar working strategy to shape both points resulted in the softer pith emerging along the sides. The shaping of the two points on the tool also resulted in retaining harder compression wood, something also remarked on for later archaeological wood weapons (61). Working away the top of the branch, while leaving the compression wood along the underside could have various explanations. The most parsimonious one is that the underside of the branch with the compression wood is hardest to work, and therefore to ease

the shaping process, more wood was removed from the softer top of the branch. However, whilst compression wood is harder, it is also more brittle which increases potential for breakage during use (62). The retention of this harder but more brittle wood could yet have a functional advantage that is not yet understood, including improved penetration if used multifunctionally as a stabbing weapon in hunting or self-defence. The question of advantages and disadvantages of compression wood is one that future use experiments of wooden tools should address.

Regardless of the material properties of the compression wood, the shaping of the points resulted in a tool that retains a slight curvature. According to Bordes' (35) classification system, completely straight throwing sticks require a circular cross-section for stability in flight. In contrast, those with a 'forme d'arc' or arc shape, consist of a gradual curvature with a constant diameter and a circular or slightly elliptical profile, and sometimes make use of a naturally curving branch. The Schöningen throwing stick appears to be somewhere between these straight and arced forms, with a moderate curvature, a natural elliptical cross-section of the shaft, and deliberate shaping of the ends into elliptical points (Figure 7; Figure 9, S5). Pointed ends in association with the arced form create drag and slow rotation in flight, somewhat reducing the maximum effective distance (35).

Tool marks provide clear evidence that this tool was made using stone tools. In particular, tool facets with signature features leave a recognisable trace that capture the tool edge and working direction. The double-pointed stick also bears evidence of abrasion to round the knots and finish the points. Use-wear analysis on lithics from the same find horizon as the double-pointed stick indicates woodworking residues on both unretouched flint flakes and scraper tools (23). With raw materials probably coming from some distance from the lakeshore, woodworking residues on flint tools at Schöningen may reflect curation including resharpening of damaged wooden weapons. A recent wider review found that in the Middle Palaeolithic bifaces and pièces esquillées were used for chopping wood, flakes and blades were utilised for shaping, carving, and working knots, while finishing work made use of scrapers and denticulates (63). Later archaeological sites and ethnographic sources show that other materials such as shells and animal teeth can also be used as woodworking tools (64–67). For the purpose of abrading wood, humans have made use of a wide variety of materials including tree bark, limestone, sandstone, pumice, handfuls of stone chips, river weed, and sea sponges (64,68–70).

4.3 Use and function

Both points are intact, the shaft break is post-excavation, and there is an absence of diagnostic impact damage. We conclude therefore that the tool was probably not discarded due to damage, and due to its location was lost during use. There is an absence of traces on either point that would relate to use of the tool as a digging stick, characterised by use-wear focused at the ends including randomly oriented striations, blunt and split tips, pitting, splinter negatives, and use polish (71). Use as a bark peeler is similarly unlikely not just because of an absence of wear at the points, but also because this artefact is beautifully worked and finely finished. As discussed above, the double-pointed stick could have had a secondary function as a short stabbing tool. In support of this are the finely worked points, shaped to retain harder compression wood, and which are not particularly aerodynamic.

The possibility of it having been a child's weapon is intriguing. Archaeological and ethnographic evidence of *H. sapiens* hunter-gatherer societies suggests children are provisioned with miniature versions of adult weapons that can be intended as toys for pretence play, learning tools, or as finely-made scaled-down weapons enabling them to participate in hunting (see 72 for a review). While (like the spears) the maximum diameter is off-centre, unlike the spears it is naturally curved. We have clearly described how the hominins worked the blank to partially straighten it, but the selection of a curved blank for a small child-sized spear seems unlikely. The longer spears, with the exception of Spear VI which has a kink, were generally straight prior to deposition (8), a key aerodynamic feature. While little is understood about spear hunting amongst Middle Pleistocene hominins, recent research shows that for contemporary foragers learning to hunt, the development of weapon skills begins in early childhood and intensifies in adolescence (73,74). However, the use of throwing sticks by children in recent societies does support the potential for such a tool to have been used at Schöningen by younger group members in games and/or communal hunting, whilst developing key hunting and throwing skills (35).

Several lines of evidence further support the original interpretation that this artefact functioned as a throwing stick (2). Throwing sticks are non-returning projectile weapons thrown in a rotational motion around their centre of gravity (35,75). Our literature review (see SI 1.5 for methods, and Accompanying Dataset 4) provided morphometric data, with lengths ranging from 58.4 cm to 103 cm, diameters between 2.2 cm and 3.5 cm, and masses between 120 g and 265 g. Both double-pointed sticks from Schöningen fit within these ranges. Ethnographic throwing sticks are morphologically variable and include straight and curved shafts with circular, elliptical or flat profiles. Ends can be shaped to have elliptical or spherical profiles. One or both ends can be uniform to the shaft or be shaped into points. Some are expediently made with little or no modification.

Ethnographically, throwing sticks were used in various scenarios including in interpersonal violence, to kill pests, in self-defence against dangerous animals including snakes, and for hunting birds, small mammals, marsupials, and larger herbivores (see Accompanying Dataset 4 for sources). Archaeological parallels include complete and fragmented wooden and mammoth ivory artefacts interpreted as throwing sticks from sites in North America (76,77), Europe (78,79), and Australia (80) (see also 35,75 for reviews). Some archaeological throwing sticks are well-crafted tools while others were only minimally worked by debarking the ends (e.g. 77). Both of the double-pointed sticks from Schöningen compare favourably with ethnographic throwing sticks, and later archaeological examples.

The two throwing sticks from Schöningen present a fascinating addition to the Middle Pleistocene toolkit, with new potentials in terms of hunting strategies, communal involvement and prey selection. While it was previously thought that the capture of small, fast prey was a behaviour not seen until the Upper Palaeolithic (81) there is increasing faunal evidence of the exploitation of birds and lagomorphs at European sites from as early as MIS 11 (82,83), albeit with an absence of evidence of the technologies with which they may have captured such animals. And because ethnographic evidence demonstrates that wooden spears are not just used to hunt large ungulates, but also for hunting small and fast terrestrial prey and for fishing (84) we must ask to what purpose would the Schöningen hominins so carefully and expertly craft these throwing sticks? However, wooden spears are not notable for their use to capture birds, a behaviour that is hinted at via the evidence of exploitation of bird remains at contemporaneous Middle Pleistocene archaeological sites. Narratives also long suggested the Middle Pleistocene and early Late Pleistocene hominins were limited by their less effective, short-distance hunting technologies in comparison with *Homo sapiens'* innovations (85). This model has been questioned on the basis of ethnographic and experimental data (30,32,84,86,87). Daasanach pastoralists achieved throwing distances of up to 110 m, while a more conservative estimate of accuracy sticks with a similar morphology to those from Schöningen is ca. 50 m (35). This more conservative distance extends estimates for hunting distances by as much as 30 additional metres (*Figure 18*). Similarly, experimentation suggests that kinetic energies of spears and throwing sticks may also be higher than the earliest spearthrowers and bows. In sum, the evidence of throwing sticks in the Middle Pleistocene alongside thrusting and throwing spears points to the use of high-energy distance weapons hundreds of thousands of years before innovations like spearthrowers and bows (Figure 18). These tools appear to represent something that is lighter, longer-distance, suitable for hunting aerial prey, and easier to use in comparison with the spears. Such tools may point to wider community involvement in hunting. Once again, the extraordinary preservation conditions at

Schöningen illustrate the sophisticated, variable and effective technologies and behaviour of Middle Pleistocene *Homo*.

Figure 18. Estimated performance parameters of five key Pleistocene weapons, and prey for which they are suitable. Sources of estimated performance data: (30–32,35,84,85,88–92). Diagram by D. Leder and A. Milks.

4.4 Taphonomic effects on preservation of traces

Taphonomic factors affected the recognition of some manufacturing traces on the tool. However, significant surface damage is constrained to specific areas. The presence of similar damage on other Schöningen wood artefacts suggests that it was likely trampled on by hoofed animals in post-depositional phase when the water-soaked wood was very soft. Further small areas of damage support our argument that the discard of the tool took place within a living wet lakeshore environment with the presence of growing plants and fungi. The effects of sediment pressure are on this piece relatively slight. Work to better understand the effects of taphonomy on the preservation of manufacturing and use traces on wood is ongoing.

5. Conclusion

Our detailed analysis of the double-pointed stick leaves no doubt that this was a well-planned, expertly manufactured, and finely-finished tool. The sequence of manufacturing stages appears to have been the removal of the branch from the tree, followed by rough removal of the branches. After oblique cuts were introduced to facilitate bark stripping, subsequent scraping was employed to fully debark the surface. Working down the knots and branch attachments likely took place after debarking, followed by shaping of regular points, partially straightening the natural bend. The final step was to abrade the surface to improve handling and/or performance, possibly alongside controlled seasoning to avoid drying cracks and harden the wood. The fine surface, carefully shaped points and use polish suggests this was a piece of personal kit with repeated use, rather than an expediently made and discarded tool. The Schöningen hominins thus had the capacity for remarkable planning depth, knowledge of raw materials, and considerable woodworking skill, resulting in an expertly designed tool (93).

At Schöningen 13 II-4 at least 11 wooden hunting weapons were found on a ca. 120 m long preserved segment of a lake shore. In contrast to earlier ideas, the find situation can best be explained by repeated hunts rather than a single episode (94). They were potentially used to assist the hunting of larger prey, but may have also been used for hunting birds and/or small mammals. Although there is to date no definitive evidence for the exploitation of small prey at Schöningen, both throwing sticks were found far removed from the spears and each in

relatively isolated locations, hinting at the potential that they may have hunted small and fast prey. The fact that the throwing stick analysed here was in one piece on discard suggests they may have been lost amongst the lakeshore reeds.

Our results show the benefits of a systematic and methodologically sound approach to the study of these important early objects. New protocols and methods to recognise micro-traces decreases observational bias, in turn enabling recognition of anthropologically modified material in absence of tools or wood fragments with a clear morphology. These new techniques, concurrent with the development of expertise in the analysis of Pleistocene wooden finds, move forward our ability to understand the cultural biographies of Pleistocene technologies and behaviours. Specifically, the application of 3D microscopy to surface features and the use of micro-CT scanning enables a detailed analysis of raw material features and manufacturing techniques employed by the humans crafting these early, supposedly simple tools. The combination of techniques applied to this single tool demonstrates the depth of understanding that can be gained from ‘zooming in’ on single objects from archaeological sites with exceptional preservation.

References

1. Aranguren B, Revedin A, Amico N, Cavulli F, Giachi G, Grimaldi S, et al. Wooden tools and fire technology in the early Neanderthal site of Poggetti Vecchi (Italy). Proc Natl Acad Sci. 2018 Jan;88:201716068.

2. Thieme H. Lower Palaeolithic hunting spears from Germany. Nature. 1997;385:807–10.

3. Warren S. Proceedings: ‘First Published Report and exhibition of the specimen, May 10th, 1911’. Q J Geol Soc. 1911 Feb;67(1–4):XCIX.

4. Meadows J, Heron C, Hüls M, Philippsen B, Weber MJ. Dating the lost arrow shafts from Stellmoor (Schleswig-Holstein, Germany): Datierung der verlorenen Pfeilschäfte aus Stellmoor (Schleswig-Holstein, Deutschland). Quartär – Int Jahrb Zur Erforsch Eiszeitalt Steinzeit. 2018;65:105–14.

5. Rust A. Die alt- und mittelsteinzeitlichen Funde von Stellmoor. Neumünster: Karl Wachholtz; 1943.

6. Terberger T, Zhilin M, Savchenko S. The Shigir idol in the context of early art in Eurasia. Quat Int. 2021 Jan;573:14–29.

7. Conard NJ, Serangeli J, Bigga G, Rots V. A 300,000-year-old throwing stick from Schöningen, northern Germany, documents the evolution of human hunting. Nat Ecol Evol. 2020 Feb;1–6.

8. Schoch WH, Bigga G, Böhner U, Richter P, Terberger T. New insights on the wooden weapons from the Paleolithic site of Schöningen. J Hum Evol. 2015 Dec;89(C):214–25.

9. Dillehay TD. Archaeological and Taphonomical Analyses of the Wood Assemblage. In: Monte Verde: A Late Pleistocene settlement in Chile Volume 2, The archaeological context and interpretation. Washington: Smithsonian Institution Press; 1997. p. 119–72.

10. López-Bultó O, Palomo A, Clemente I. Tool mark analysis of Neolithic wooden digging sticks from La Draga (Banyoles, Spain). Quat Int. 2020 Dec 20;569–570:39–50.

11. Revedin A, Grimaldi S, Florindi S, Santaniello F, Aranguren B. Experimenting the Use of Fire in the Operational Chain of Prehistoric Wooden Tools: the Digging Sticks of Poggetti Vecchi (Italy). J Paleolit Archaeol. 2019 Dec;1–12.

12. Rios-Garaizar J, López-Bultó O, Iriarte E, Pérez-Garrido C, Piqué R, Aranburu A, et al. A Middle Palaeolithic wooden digging stick from Aranbaltza III, Spain. PLoS ONE. 2018 Mar;13(3):e0195044-15.

13. Bamforth M, Taylor M, Little A, Radini A. Woodworking Technology. In: Milner N, Conneller C, Taylor M, editors. Star Carr Volume 2: studies in technology, subsistence and environment. Heslington: White Rose University Press; 2018. p. 347–66.

14. Vidal-Matutano P, Rodríguez-Rodríguez A, González-Marrero M del C, Morales J, Henríquez-Valido P, Moreno-Benítez MA. Woodworking in the cliffs? Xylological and morpho-technological analyses of wood remains in the Prehispanic granaries of Gran Canaria (Canary Islands, Spain). Quat Int. 2021 Aug 20;593–594:407–23.

15. Thieme H. Lower Palaeloithic Throwing Spears and Other Wooden Implements From Schoeningen, Germany. In: Ullrich H, editor. Hominid evolution : lifestyles and survival strategies. Gelsenkirchen Germany : Edition Archaea; 1999. p. 383–95.

16. Böhner U, Serangeli J, Richter P. The Spear Horizon: First spatial analysis of the Schöningen site 13 II-4. J Hum Evol. 2015 Dec;89:202–13.

17. Bigga G, Schoch WH, Urban B. Paleoenvironment and possibilities of plant exploitation in the Middle Pleistocene of Schöningen (Germany). Insights from botanical macro-remains and pollen.J Hum Evol. 2015 Dec;89:92–104.

18. Urban B, Bigga G. Environmental reconstruction and biostratigraphy of late Middle Pleistocene lakeshore deposits at Schöningen. 2015 J Hum Evol. Dec;89:57–70.

19. Richter D, Krbetschek M. The age of the Lower Paleolithic occupation at Schöningen. J Hum Evol. 2015 Dec;89(C):46–56.

20. Van Kolfschoten T, Buhrs E, Verheijen I. The larger mammal fauna from the Lower Paleolithic Schöningen Spear site and its contribution to hominin subsistence. J Hum Evol. 2015 Dec;89(C):138–53.

21. Serangeli J, Van Kolfschoten T, Starkovich BM, Verheijen I. The European saber-toothed cat (Homotherium latidens) found in the ‘Spear Horizon’ at Schöningen (Germany). J Hum Evol. 2015 Dec;89:172–80.

22. Van Kolfschoten T, Parfitt SA, Serangeli J, Bello SM. Lower Paleolithic bone tools from the `Spear Horizon’ at Schöningen (Germany). J Hum Evol. 2015 Dec;89(C):226–63.

23. Rots V, Hardy BL, Serangeli J, Conard NJ. Residue and microwear analyses of the stone artifacts from Schöningen. J Hum Evol. 2015 Dec;89(C):298–308.

24. Serangeli J, Böhner U. Die Artefakte von Schöningen und ihre Zeitliche Einordnung. In: Die chronologische Einordnung der paläolithischen Fundstellen von Schöningen–Forschungen zur Urgeschichte aus dem Tagebau von Schöningen [Internet]. 2012. p. 23–37. (Bd 1).

25. Preece RC, Gowlett JAJ, Parfitt SA, Bridgland DR, Lewis SG. Humans in the Hoxnian: habitat, context and fire use at Beeches Pit, West Stow, Suffolk, UK. J Quat Sci. 2006;21(5):485–96.

26. Ravon AL, Monnier JL, Laforge M. Menez-Dregan I, layer 4: A transitional layer between the Lower and Middle Palaeolithic in Brittany. Quat Int. 2016 Jul;409(Part B):92–103.

27. Kretzoi M, Dobosi VT. Vértesszőlős: Site, man and culture. Akadémiai Kiadó; 1990.

28. Scott RV, Hosfield R. Fire in the round: A holistic approach to the Lower Palaeolithic record. J Archaeol Sci Rep. 2021 Jun 1;37:102938.

29. Stahlschmidt MC, Miller CE, Ligouis B, Hambach U, Goldberg P, Berna F, et al. On the evidence for human use and control of fire at Schöningen. J Hum Evol. 2015 Dec;89:181–201.

30. Milks A, Parker D, Pope M. External ballistics of Pleistocene hand-thrown spears: experimental performance data and implications for human evolution. Sci Rep. 2019 Jan;9(1):820.

31. Milks A, Champion S, Cowper E, Pope M, Carr D. Early spears as thrusting weapons: Isolating force and impact velocities in human performance trials. J Archaeol Sci Rep. 2016 Dec;10:191–203.

32. Rieder H. Erprobung der Holzspeere von Schoeningen (400000 Jahre) und Folgerungen daraus. In: Wagner GA, Mania D, editors. Fruhe Menschen in Mittel Europa: Chronologie, Kultur, Umwelt. Aachen: Shaker; 2001. p. 91–8.

33. Milks A. Lethal Threshold: The Evolutionary Implications of Middle Pleistocene Wooden Spears [Internet] [PhD Thesis]. 2018. Available from: https://discovery.ucl.ac.uk/id/eprint/10045809/1/MILKS%20PhD%20Final.compressed%20no%203%20party.pdf

34. Braovac S, McQueen C, Sahlstedt M, Kutzke H, \Lucejko JJ, Klokkernes T. Navigating conservation strategies: linking material research on alum-treated wood from the Oseberg collection to conservation decisions. Herit Sci. 2018;6(1):1–16.

35. Bordes L. Les bâtons de jet préhistoriques et leurs représentations: Développement d’outils et de méthodes pour la mesure de leurs caractéristiques et l’évaluation de leurs fonctions [Internet] [Master’s Thesis]. 2014.

36. Faix O. Classification of lignins from different botanical origins by FT-IR spectroscopy. Holzforschung. 1991; 45; 21-27

37. Fengel D. Influence of water on the OH valency range in deconvoluted FTIR spectra of cellulose. 1993;

38. Pandey K. A study of chemical structure of soft and hardwood and wood polymers by FTIR spectroscopy. J Appl Polym Sci. 1999;71(12):1969–75.

39. Smidt E, Tintner J, Klemm S, Scholz U. FT-IR spectral and thermal characterization of ancient charcoals-A tool to support archeological and historical data interpretation. Quat Int. 2017;457:43–9.

40. Smith B. Infrared spectral interpretation: a systematic approach. CRC press; 1999.

41. Kwon SM, Jang JH, Lee SH, Park SB, Kim NH. Change of heating value, pH and FT-IR spectra of charcoal at different carbonization temperatures. J Korean Wood Sci Technol. 2013;41(5):440–6.

42. Sharma RK, Wooten JB, Baliga VL, Lin X, Chan WG, Hajaligol MR. Characterization of chars from pyrolysis of lignin. Fuel. 2004;83(11–12):1469–82.

43. Yang Y, Fu T, Song F, Song X, Wang XL, Wang YZ. Wood-burning processes in variable oxygen atmospheres: Thermolysis, fire, and smoke release behavior. Polym Degrad Stab. 2022;205:110158.

44. Richter C. Wood Characteristics [Internet]. Cham: Springer International Publishing; 2015 [cited 2021 Jul 14]. Available from: http://link.springer.com/10.1007/978-3-319-07422-1

45. Milks A, Lehmann J, Leder D, Böhner U, Koddenberg T, Sietz M, et al. Wood technology: a Glossary and Code for the analysis of archaeological wood from stone tool cultures [Internet]. OSF Preprints x8m4j, ver. 7 peer-reviewed and recommended by Peer community in Archaeology.; 2022. Available from: https://osf.io/x8m4j/

46. Martellotta EF, Wilkins J, Brumm A, Langley MC. New data from old collections: Retouch-induced marks on Australian hardwood boomerangs. J Archaeol Sci Rep. 2021 Jun;37:102967.

47. Van de Noort R, Ellis S, Taylor M, Wier D. Preservation of archaeological sites. In: Van de Noort R, Ellis S, editors. Wetland Heritage of Holderness: An Archaeological Survey. English Heritage; 1995. p. 341–56.

48. Soressi M, Geneste JM. Reduction sequence, chaîne opératoire, and other methods: the epistemologies of different approaches to lithic analysis the history and efficacy of the chaîne opératoire approach to lithic analysis: studying techniques to reveal past societies in an evolutionary perspective. PaleoAnthropology. 2011;2011:334–50.

49. Ruelle J. Morphology, anatomy and ultrastructure of reaction wood. In: The biology of reaction wood. Springer; 2014. p. 13–35.

50. Wardrop A, Dadswell H. The nature of reaction wood. IV. Variations in cell wall organization of tension wood fibres. Aust J Bot. 1955;3(2):177–89.

51. Rosenthal M, Bäucker E. Druckholz-Reaktionsholz der Nadelhölzer: Ausgewählte Eigenschaften und wesentliche Unterschiede zum normalen Holzgewebe. Technische Universität Dresden, Fakultät Forst-, Geo-und Hydrowissenschaften; 2012.

52. Vaganov EA, Hughes MK, Shashkin AV. Growth dynamics of conifer tree rings: images of past and future environments. Vol. 183. Springer Science & Business Media; 2006.

53. Muller A. Jahrringanalytische Untersuchungen zum Informationsgehalt von Holzkohle-Ruckstanden der historischen Meilerkohlerei [PhD Thesis]. Albert-Ludwigs-Universitat Freiburg; 2007.

54. Blanchette RA. A review of microbial deterioration found in archaeological wood from different environments. Int Biodeterior. 2000;16:189-204.

55. Gaspari A, Eric M, Odar B. A Palaeolithic Wooden Point from Ljubljansko Barje, Slovenia. In: Benjamin J, Bonsall C, Pickard C, Fischer A, editors. Submerged Prehistory [Internet]. Oxbow Books; 2011. p. 186–92. Available from: http://www.academia.edu/download/30503575/2011_Mesolithic_hunter-fishers_in_a_changing_world_Jackelberg.pdf

56. Oakley KP, Andrews P, Keeley LH, Clark JD. A Reappraisal of the Clacton Spearpoint. Proc Prehist Soc. 1977 Dec;43:13–30.

57. Schoch W. Holzanatomische Nachuntersuchungen an der eemzeitlichen Holzlanze von Lehringen, Ldkr. Verden. Nachrichten Aus Niedersachs Urgesch. 2014;83:19–29.

58. Thieme H, Veil S. Neue Untersuchungen zum eemzeitlichen Elefanten-Jagdplatz Lehringen, Ldkr. Verden. Kunde. 1985;36:11–58.

59. Stanzl-Tschegg SE, Keunecke D, Tschegg EK. Fracture tolerance of reaction wood (yew and spruce wood in the TR crack propagation system). J Mech Behav Biomed Mater. 2011 Jul;4(5):688–98.

60. Pournou A. Biodeterioration of Wooden Cultural Heritage: Organisms and Decay Mechanisms in Aquatic and Terrestrial Ecosystems [Internet]. Cham: Springer International Publishing; 2020 [cited 2022 Feb 11]. Available from: http://link.springer.com/10.1007/978-3-030-46504-9

61. Friedman J. The Prehistoric Uses Of Wood at the Ozette Archaeological Site [Internet] [PhD Thesis]. Washington State University; 1975 [cited 2022 Jan 10]. Available from: https://www.proquest.com/pqdtglobal/docview/302796500/BD7A4E9EDCB740CDPQ/1?accountid=14511

62. Kozlowski TT, Pallardy SG. Growth control in woody plants. Elsevier; 1997.

63. Bencomo Viala M, Jardón Giner P, Sirvent Cañada LM, Hortelano Piqueras L. Understanding woodworking in Paleolithic times by means of use-wear analysis. J Archaeol Sci Rep. 2020 Feb;29:102119.

64. Deacon B, Wedgwood C. Malekula: A Vanishing People In The New Hebrides. 1934; Available from: http://ehrafworldcultures.yale.edu/document?id=oo12-004

65. Gould RA. Yiwara: foragers of the Australian Desert. London: Collins; 1969.

66. Lozovskaya OV, Lozovski VM. Mode de fabrication des outils en bois dans le Mésolithique d'Europe Orientale: approche expérimentale-tracéologique. Experimentación en arqueología: Estudio y difusión del pasado 2013;73–83.

67. Weston E, Szabó K, Stern N. Pleistocene shell tools from Lake Mungo lunette, Australia: Identification and interpretation drawing on experimental archaeology. Quat Int. 2017 Jan;427:229–42.

68. Best E. The Maori: Volume 2. 1924;5. Available from: http://ehrafworldcultures.yale.edu/document?id=oz04-002

69. LeBar FM. The Material Culture of Truk [Internet]. New Haven, CT: Yale University; 1964. Available from: http://ehrafworldcultures.yale.edu/document?id=or19-003

70. MacCalman HR, Grobbelaar BJ. Preliminary report of two stone-working OvaTjimba groups in the northern Kaokoveld of South West Africa. Cimbebasia SWA Res. 1965;13:1–39.

71. Nugent SJ. Applying use-wear and residue analysis to digging sticks. Mem Qld Mus Cult Herit Ser. 2006 Nov;4(1):89–105.

72. Milks A, Lew-Levy S, Lavi N, Friesem DE, Reckin R. Hunter-gatherer children in the past: An archaeological review. J Anthropol Archaeol. 2021 Dec;64:101369.

73. Lew-Levy S, Bombjaková D, Milks A, Kiabiya Ntamboudila F, Kline MA, Broesch T. Costly teaching contributes to the acquisition of spear hunting skill among BaYaka forager adolescents. Proc R Soc B Biol Sci. 2022 May 11;289(1974):20220164.

74. MacDonald K. Cross-cultural Comparison of Learning in Human Hunting. Hum Nat Hawthorne N. 2007 Oct;18(4):386–402.

75. Bordes L. Throwing Stick to Spear Thrower - Study of Ethnographic Artefacts and Experimentation. EXARC J. 2020;(2020/2).

76. Koerper HC. A Game String and Rabbit Stick Cache from Borrego Valley, San Diego County. J Calif Gt Basin Anthropol. 1998;252–70.

77. Dalley GF. Artifacts of wood. In: Aikins MC, editor. Hogup Cave. Salt Lake City: University of Utah Press; 1970. p. 153–86.

78. Valde-Nowak P, Nadachowski A, Wolsan M. Upper Palaeolithic boomerang made of a mammoth tusk in south Poland. Nature. 1987 Oct;329(6138):436–8.

79. Andersen SH. Marinarkæologiske undersøgelser af kystboplads fra Ertebølletid. Ronæs Skov Jysk Arkæol Selsk. 2009;64.

80. Luebbers RA. Ancient boomerangs discovered in South Australia. Nature. 1975;253(5486):39–39.

81. Stiner MC, Munro ND, Surovell TA. The tortoise and the hare. Curr Anthropol. 2000;41(1):39–79.

82. Blasco R, Rosell J, Peris JF, Arsuaga JL, Bermúdez de Castro JM, Carbonell E. Environmental availability, behavioural diversity and diet: a zooarchaeological approach from the TD10-1 sublevel of Gran Dolina (Sierra de Atapuerca, Burgos, Spain) and Bolomor Cave (Valencia, Spain). Quat Sci Rev. 2013 Jun;70(C):124–44.

83. Morin E, Meier J, El Guennouni K, Moigny AM, Lebreton L, Rusch L, et al. New evidence of broader diets for archaic Homo populations in the northwestern Mediterranean. Sci Adv. 2019 Mar;5(3):eaav9106.

84. Milks A. A Review of Ethnographic Use of Wooden Spears and Implications for Pleistocene Hominin Hunting. Open Quat. 2020 Sep;6(1):79–20.

85. Churchill SE. Weapon Technology, Prey Size Selection, and Hunting Methods in Modern Hunter-Gatherers: Implications for Hunting in the Palaeolithic and Mesolithic. Archeol Pap Am Anthropol Assoc. 1993;4(1):11–24.

86. Villa P, Lenoir M. Hunting weapons of the Middle Stone Age and the Middle Palaeolithic: spear points from Sibudu, Rose Cottage and Bouheben. South Afr Humanit. 2006;18(1):89–122.

87. Villa P, Soriano S. Hunting weapons of Neanderthals and early modern humans in South Africa: similarities and differences. J Anthropol Res. 2010;66:5–38.

88. Coppe J, Lepers C, Clarenne V, Delaunois E, Pirlot M, Rots V. Ballistic Study Tackles Kinetic Energy Values of Palaeolithic Weaponry. Archaeometry. 2019 Feb;2(4):107–24.

89. Roach NT, Richmond BG. Clavicle length, throwing performance and the reconstruction of the Homo erectus shoulder. J Hum Evol. 2015 Mar;80:107–13.

90. Tomka SA. The adoption of the bow and arrow: a model based on experimental performance characteristics. Am Antiq. 2013;78(03):553–69.

91. Whittaker JC, Pettigrew DB, Grohsmeyer RJ. Atlatl Dart Velocity: Accurate Measurements and Implications for Paleoindian and Archaic Archaeology. PaleoAmerica. 2017 Apr;3(2):161–81.

92. Whittaker JC, Kamp KA. Primitive Weapons and Modern Sport: Atlatl Capabilities, Learning, Gender, and Age. Plains Anthropol. 2006;51(198):213–21.

93. Haidle MN. How to think a simple spear. In: In S De Beaune, F Coolidge & T Wynn (eds) Cognitive Archaeology and Human Evolution [Internet]. Cambridge: Cambridge University Press; 2009. p. 57–174. Available from: http://books.google.com/books?hl=en&lr=&id=yBnCOc8Tr_wC&oi=fnd&pg=PA57&dq=How+to+think+a+simple+spear&ots=n1pNXdAAPX&sig=Iw61sV-Dh4M2J9HlPEmm0JMmpUM

94. Rivals F, Julien MA, Kuitems M, Van Kolfschoten T, Serangeli J, Drucker DG, et al. Investigation of equid paleodiet from Schöningen 13 II-4 through dental wear and isotopic analyses: Archaeological implications. J Hum Evol. 2015 Dec;89:129–37.

Acknowledgements

We thank Waygate Technologies for the micro-CT scanning and Bernhard Schartel and Yannik Wägner at the Bundesanstalt für Materialforschung und -prüfung for their contributions towards the FTIR analysis. Many thanks to GOM Metrology for the 3D scanning of the object and to Hartmut Thieme and Werner Schoch for previous work on the Schöningen artefacts. We are grateful to Anna-Laura Krogmeier and all the staff at the Forschungsmuseum Schöningen for facilitating access to the artefact.

Funding

The study was supported by the Deutsche Forschungsgemeinschaft (DFG, German Research Foundation) – project number 447423357. AM is funded by a British Academy Postdoctoral Fellowship (PF21/210027).

Author Contributions

J.L., A.M., T.T., T.K. and D.L. recorded traces on the artefact. M.S. conducted the wood species analysis and tree ring and growth rate analysis, oversaw the production of the micro-CT scan, and together with T.K. created the micro-CT slices for further analysis. V.W. conducted the ATR-FTIR analysis. U.B. oversaw the production of the 3D model. T.K. and J.L. conducted the 3D digital microscopy and 3D morphometrics. A.M. undertook the ethnographic

literature review. D.L., J.L., T.K., M..S., A.M., V.W. and U.B. prepared images. All authors contributed to writing the manuscript.

Figure 1 Click here to access/download;Figure;Figure 1.jpg

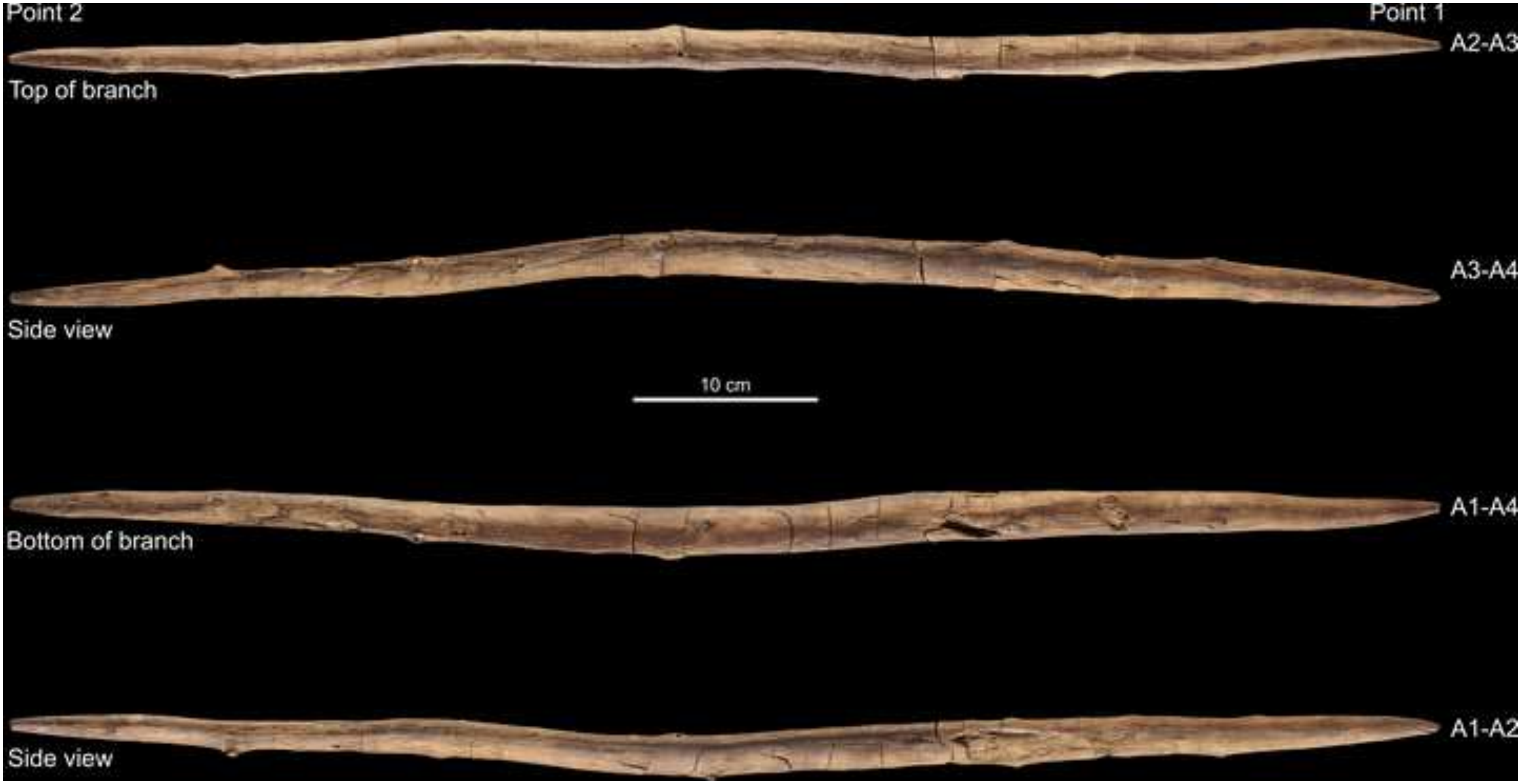

Figure 2

Click here to access/download;Figure;Figure 2.jpg

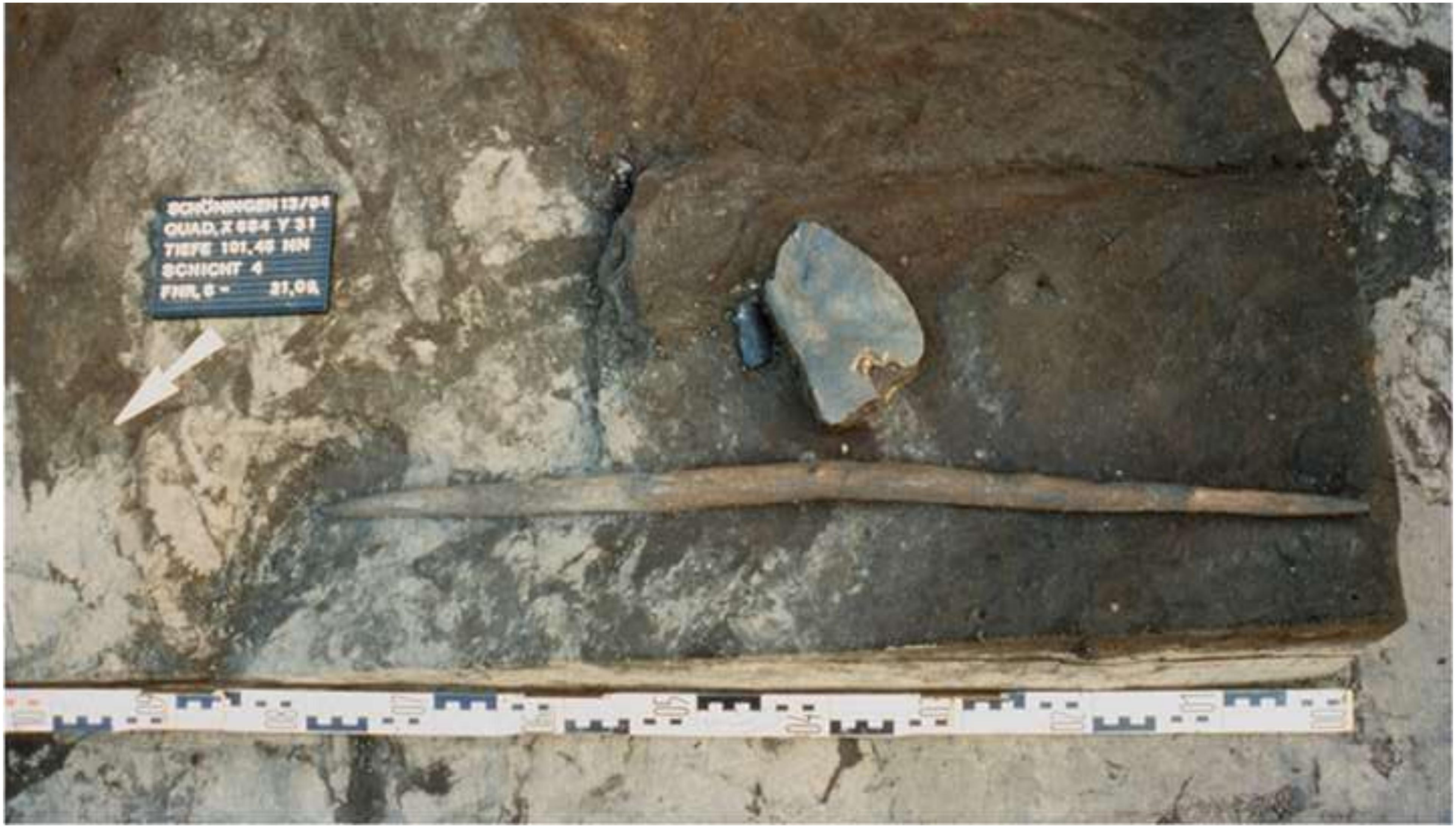

Figure 3

Click here to access/download;Figure;Figure 3.tiff

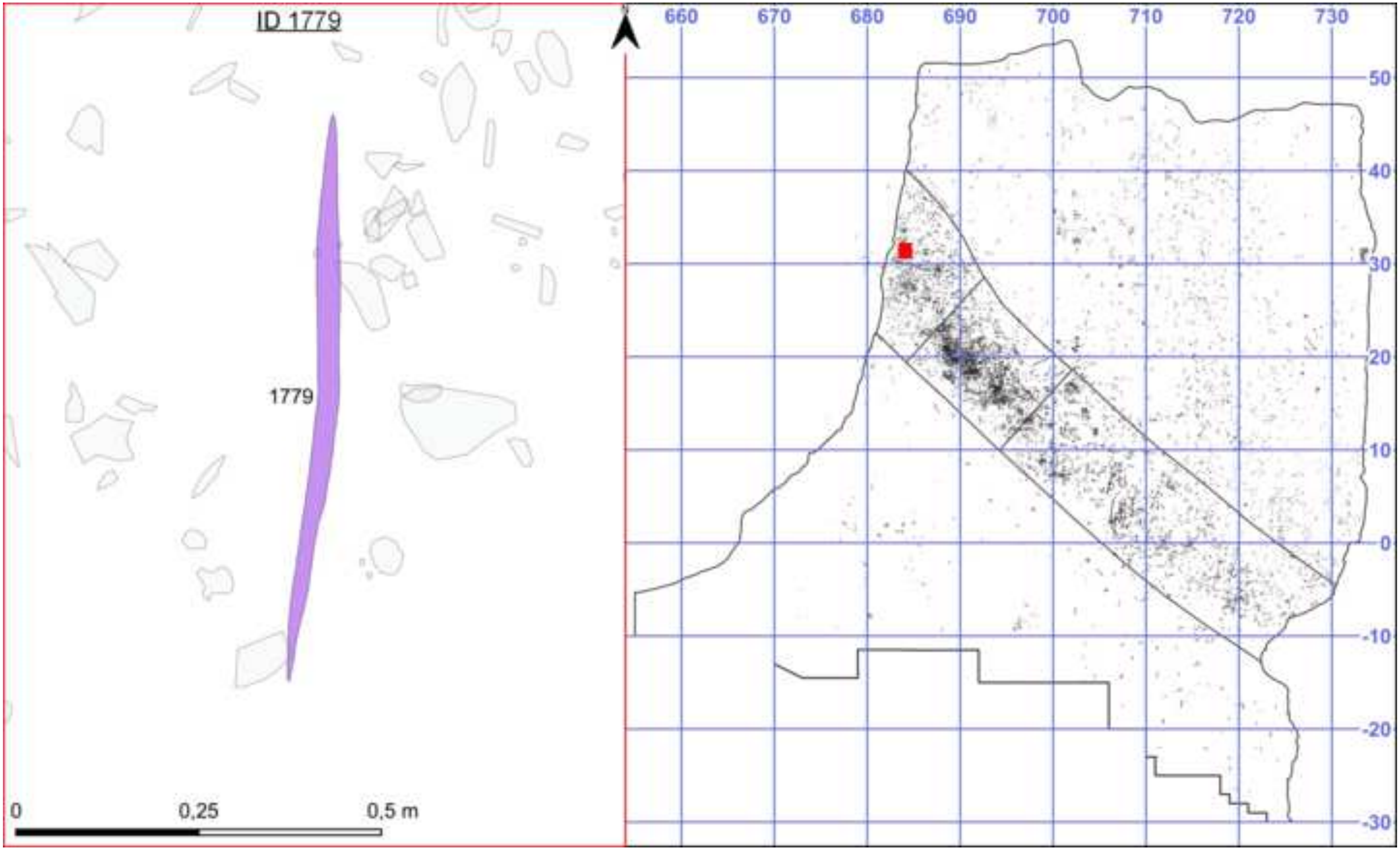

Figure 4

Click here to access/download;Figure;Figure 4.jpg

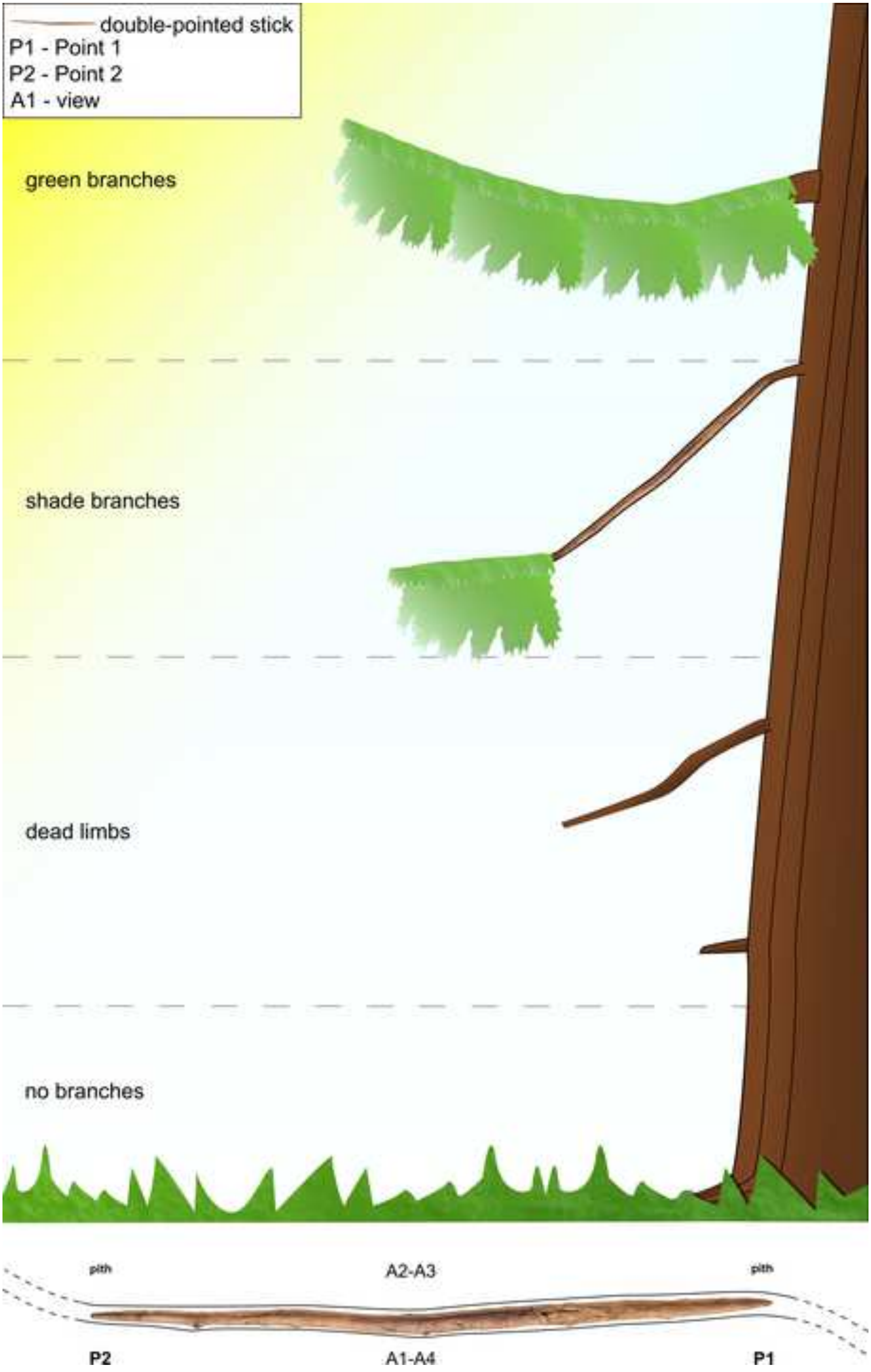

Click here to access/download;Figure;Figure 5.tiff

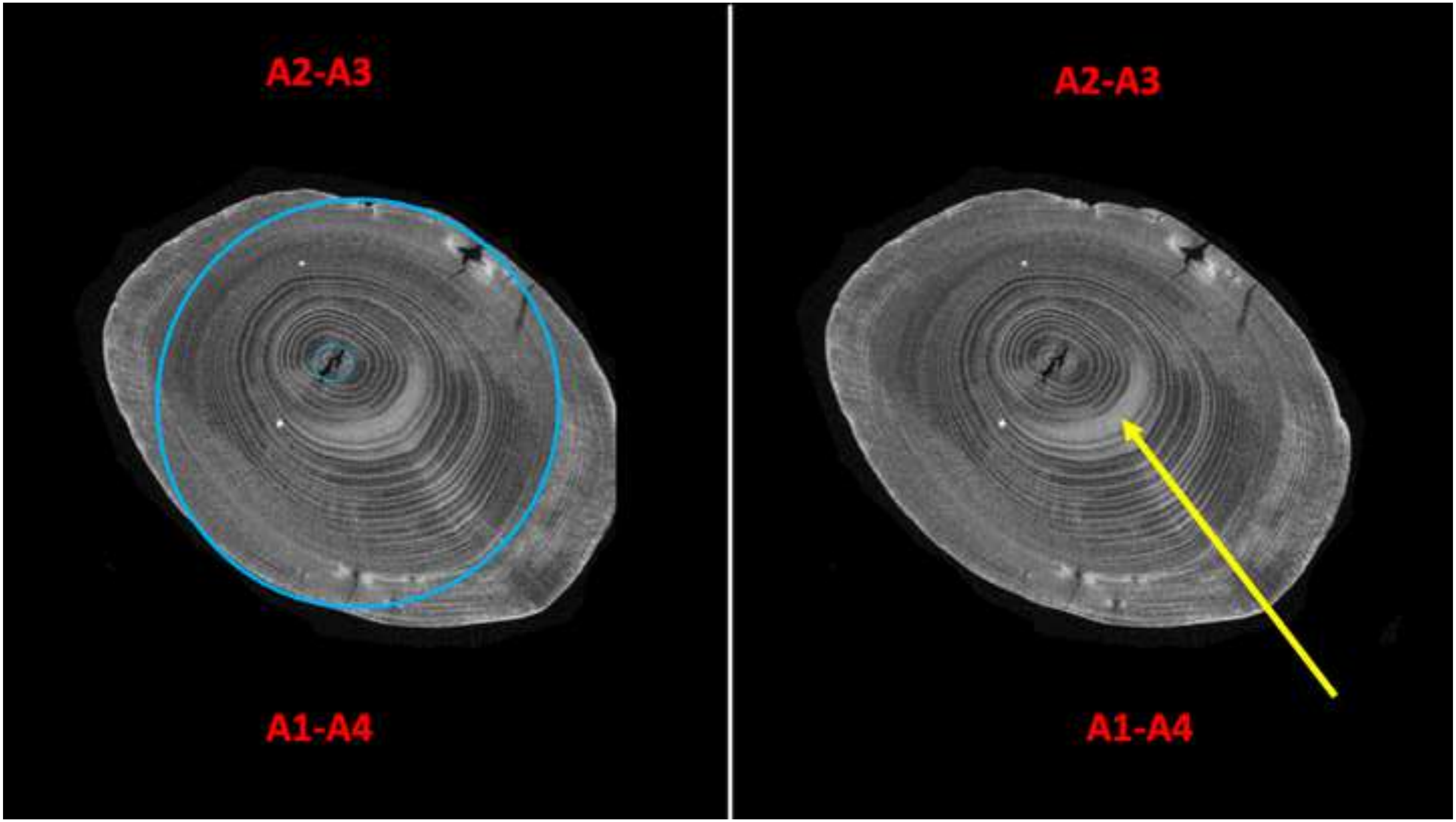

Figure 6

Click here to access/download;Figure;Figure 6.jpg

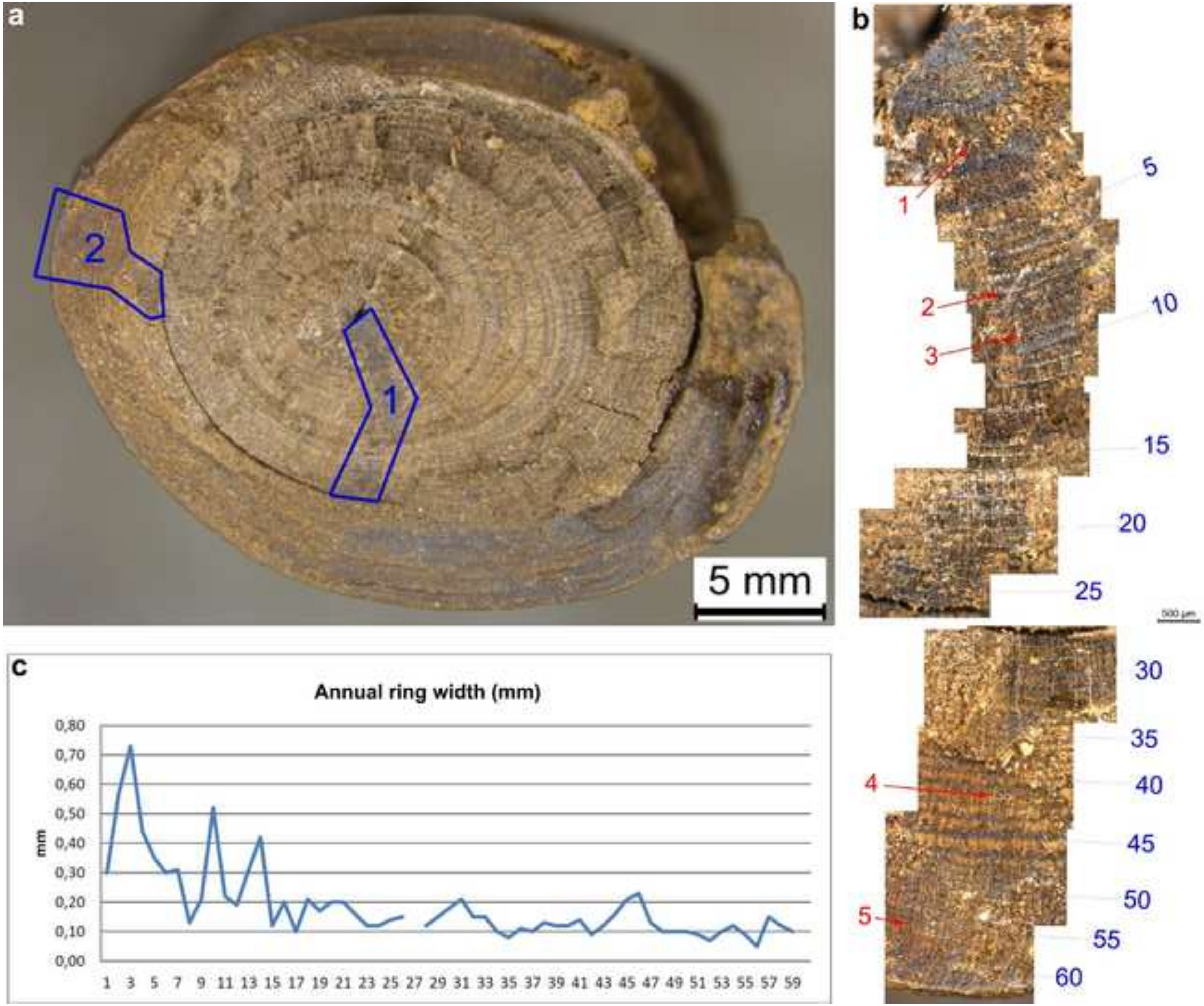

Figure 7

Click here to access/download;Figure;Figure 7.tiff

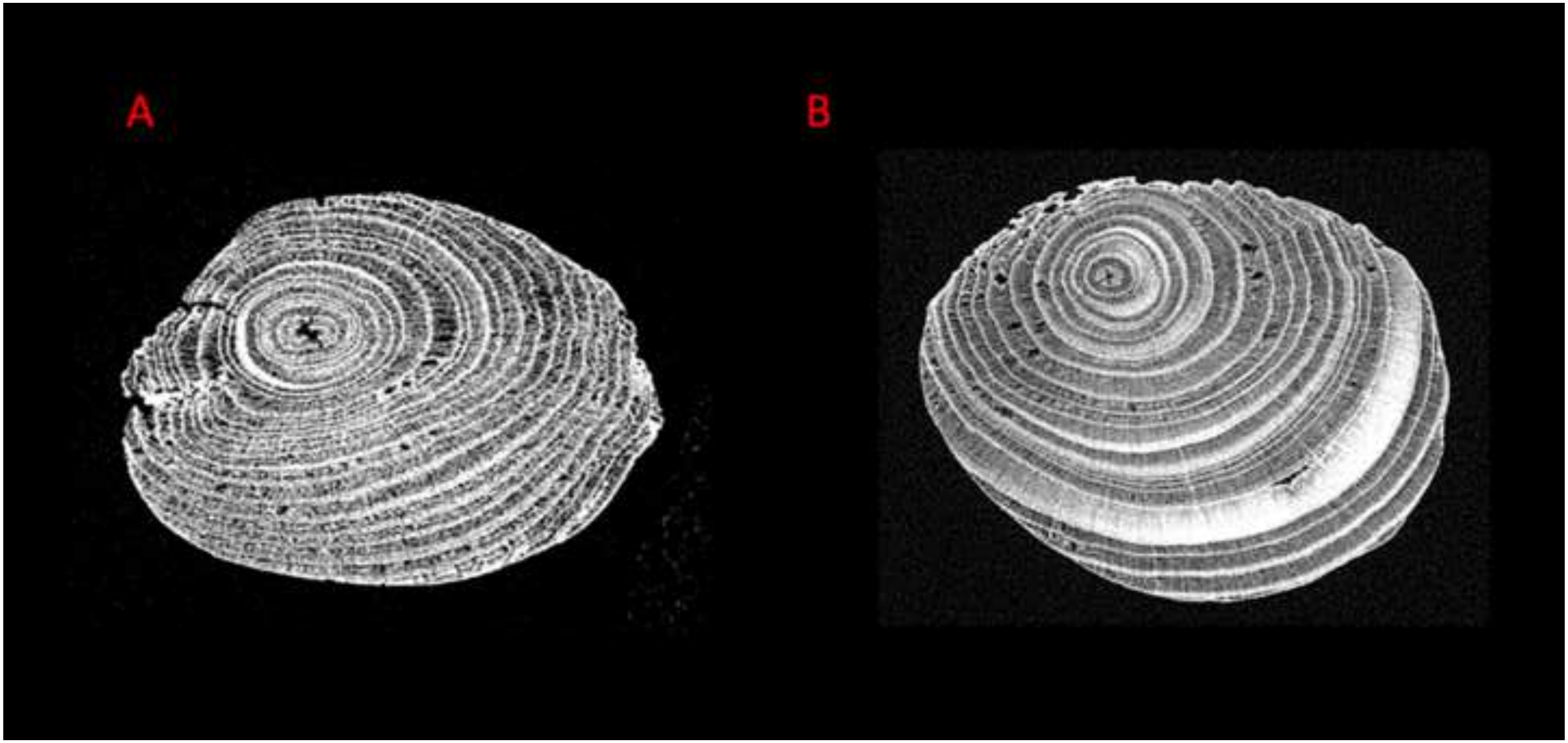

Figure 8

Click here to access/download;Figure;Figure 8.tiff

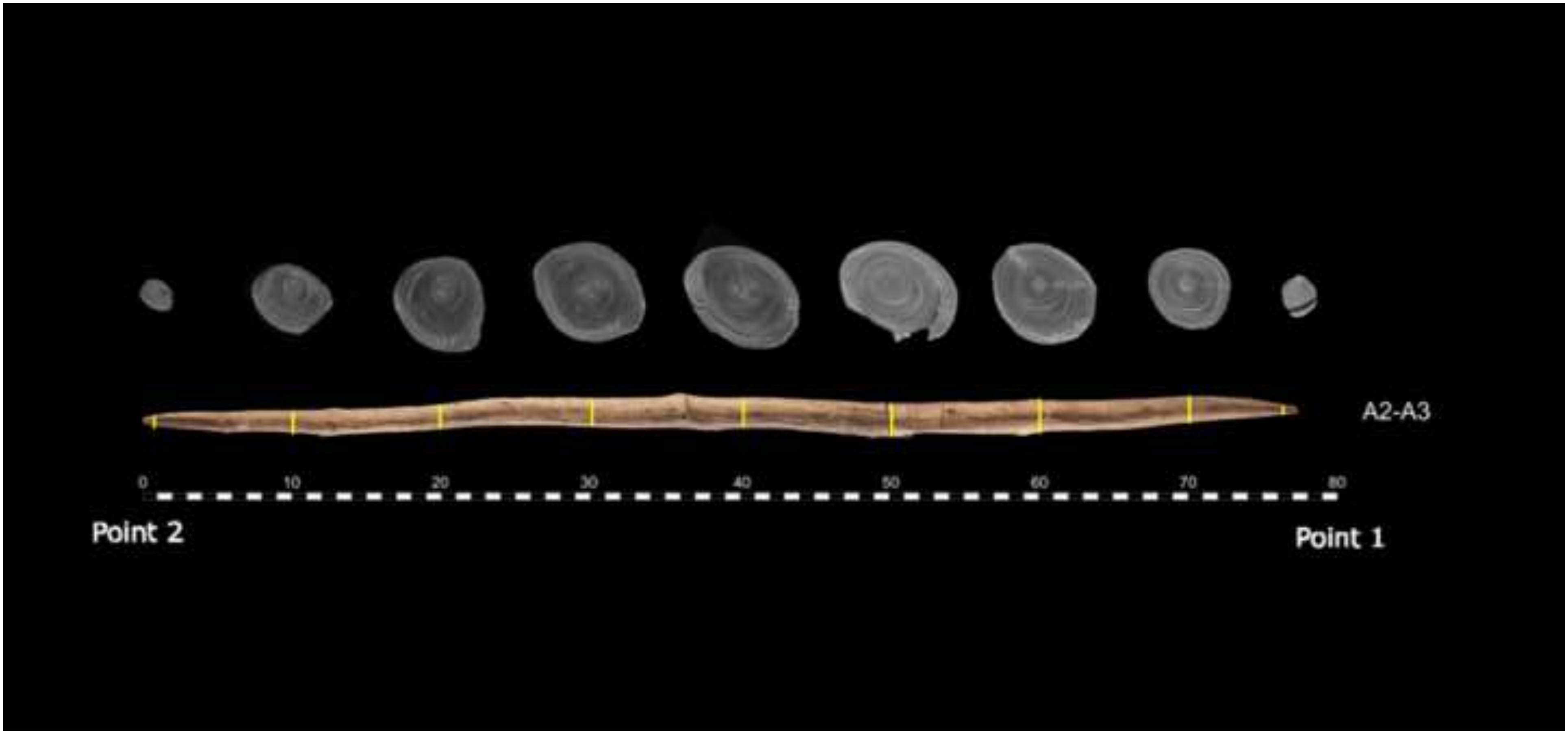

Figure 9

Click here to access/download;Figure;Figure 9.tiff

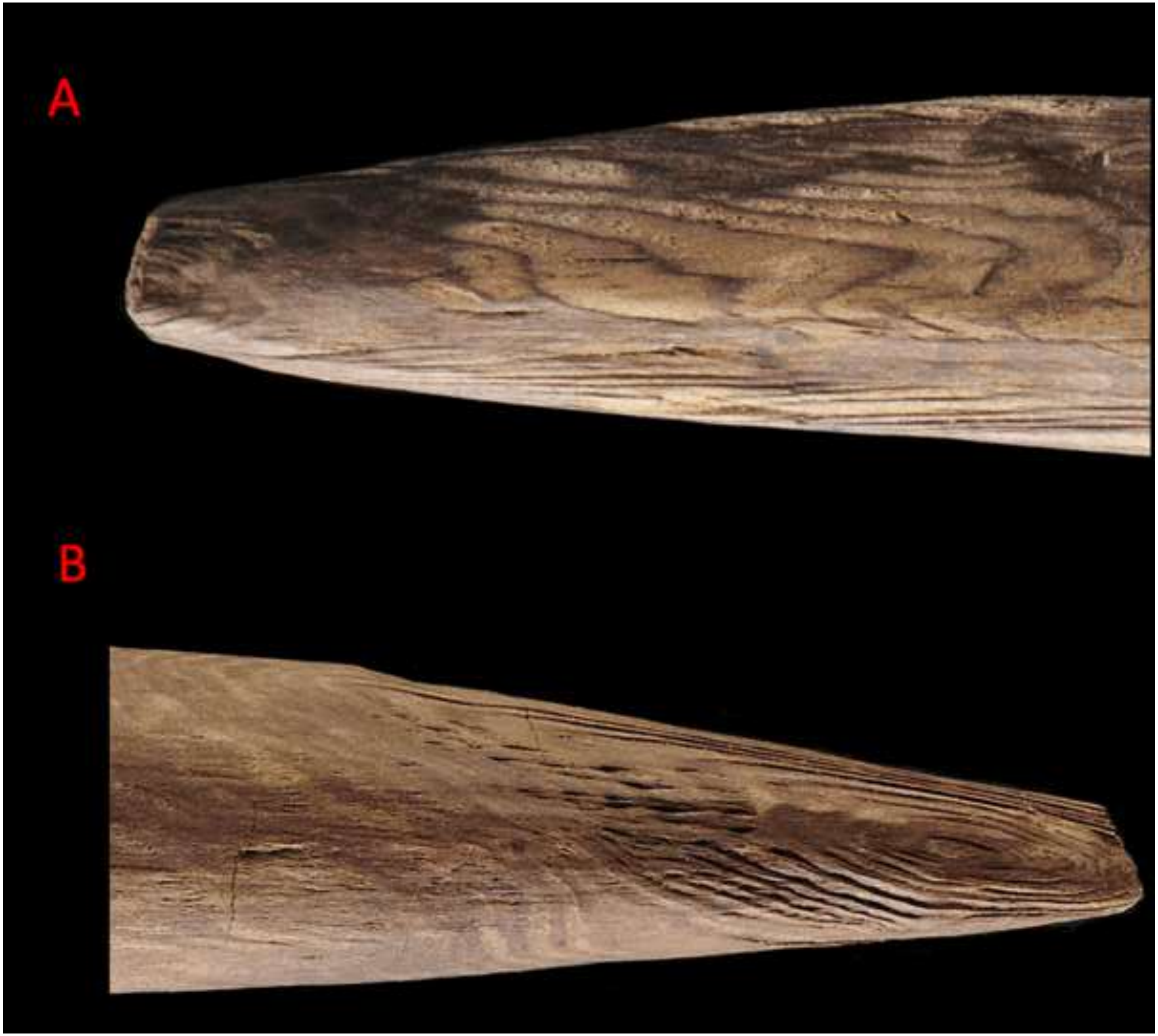

Figure 10

Click here to access/download;Figure;Figure 10.jpg

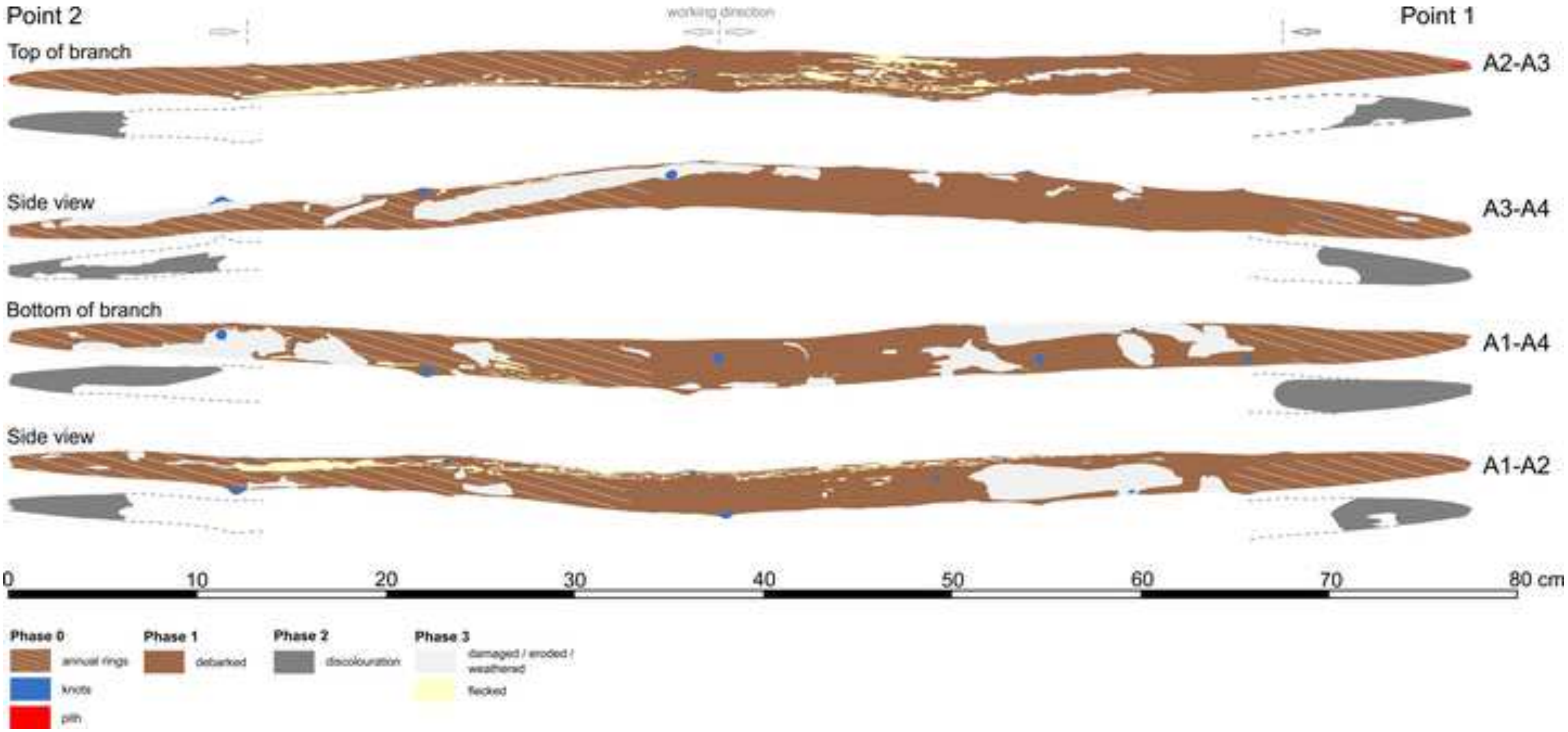

Figure 11

Click here to access/download;Figure;Figure 11.tiff

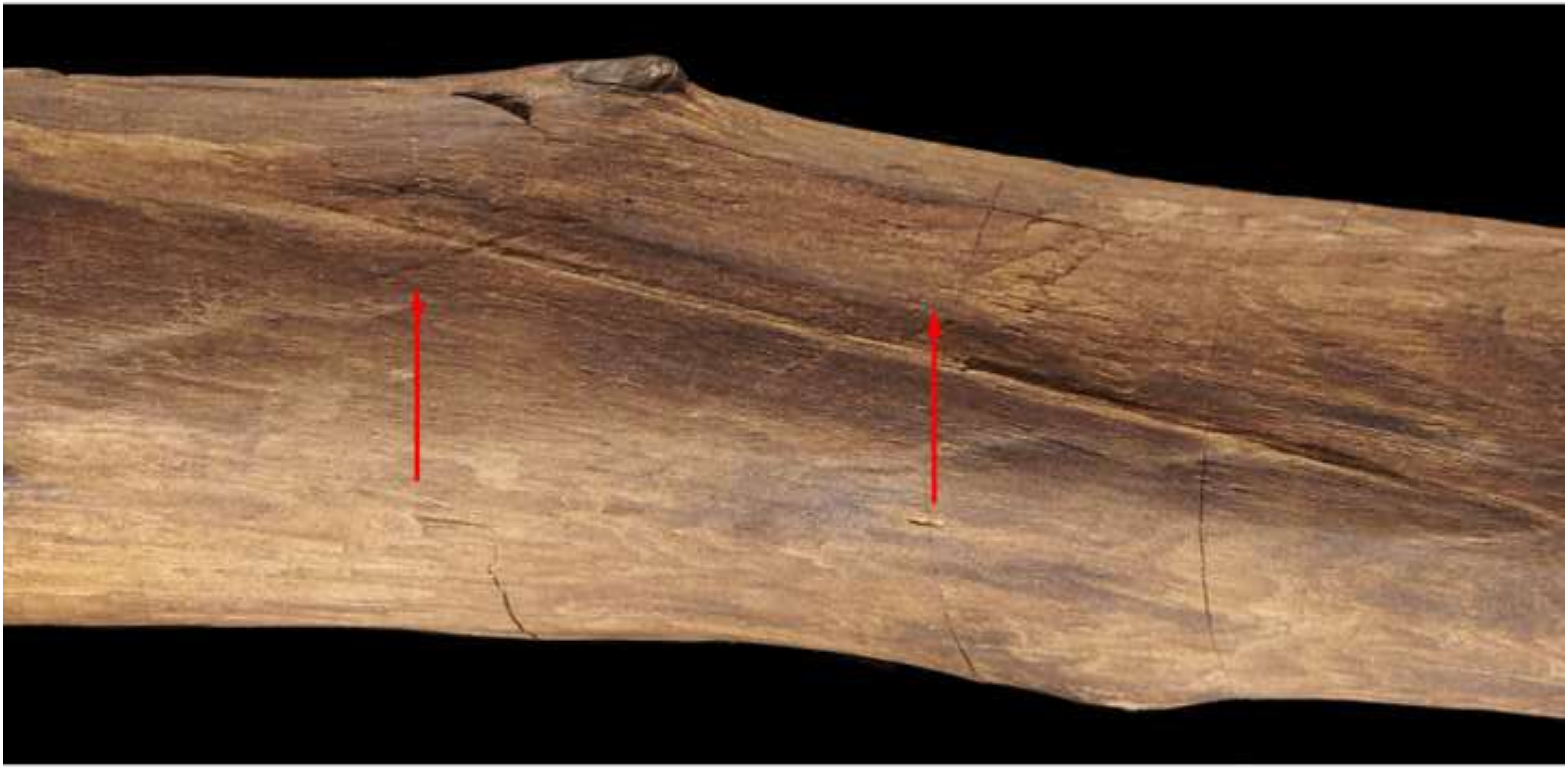

Figure 12

Click here to access/download;Figure;Figure 12.tiff

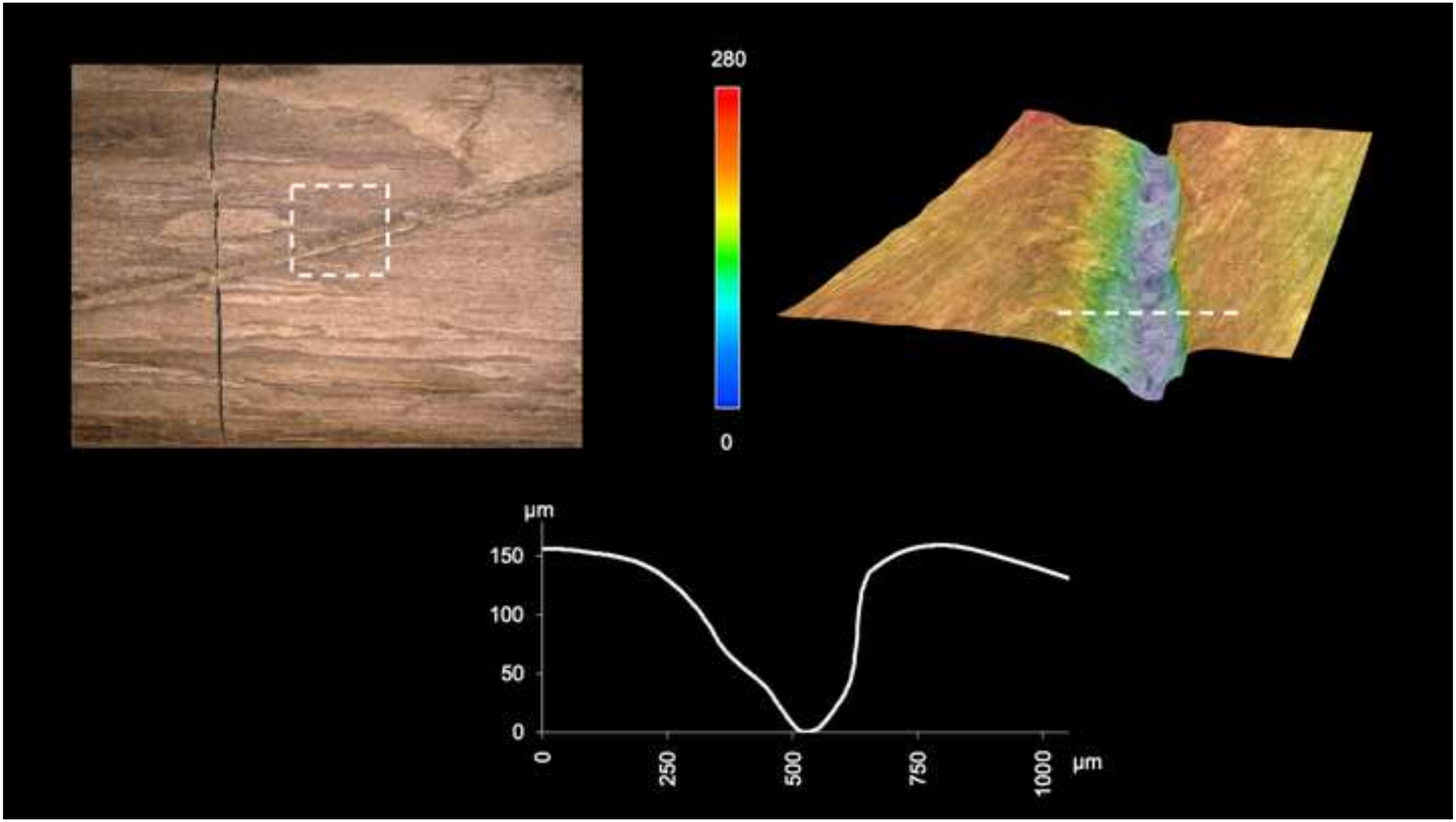

Figure 13

Click here to access/download;Figure;Figure 13.jpg

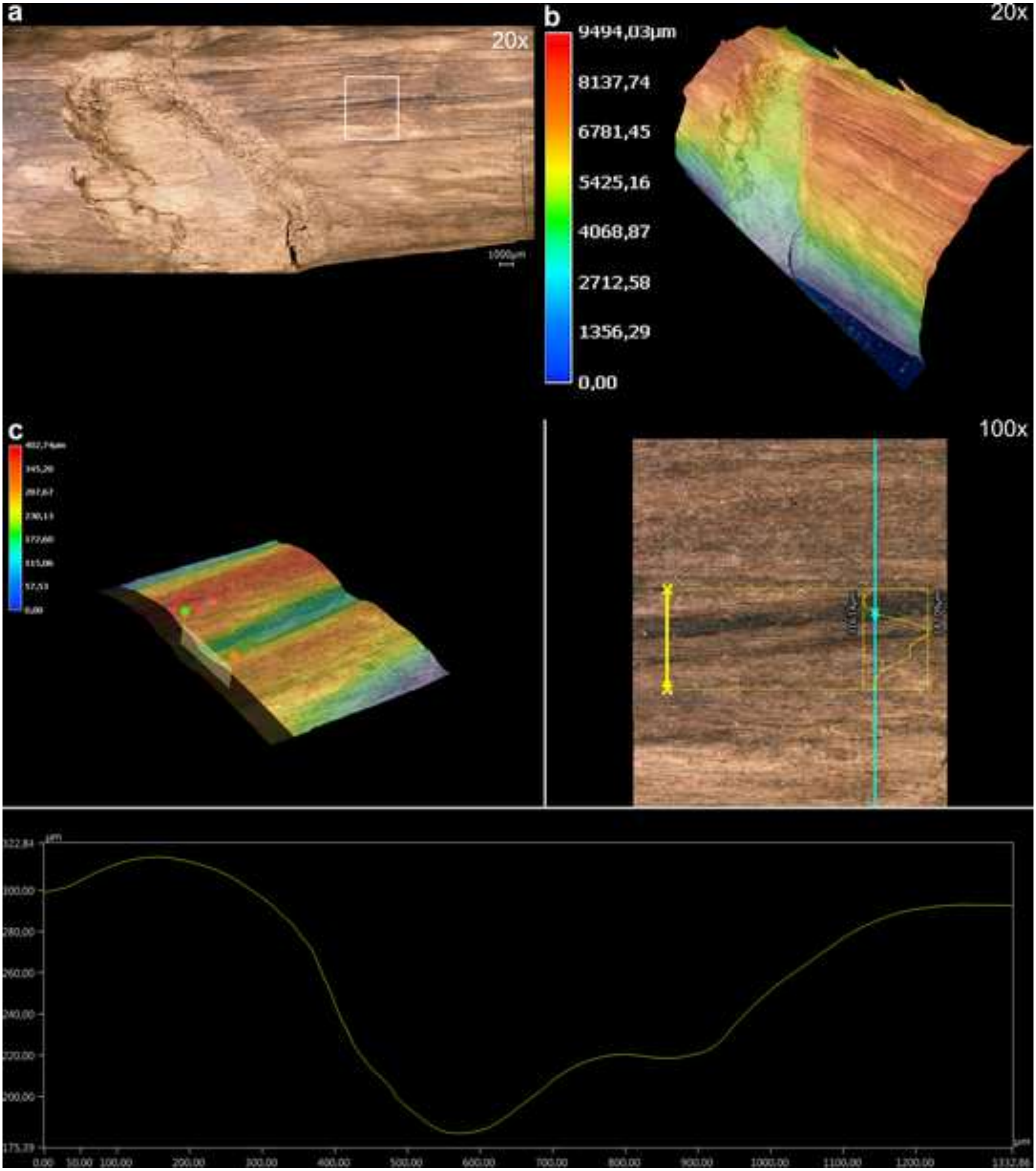

Figure 14 Click here to access/download;Figure;Figure 14.jpg

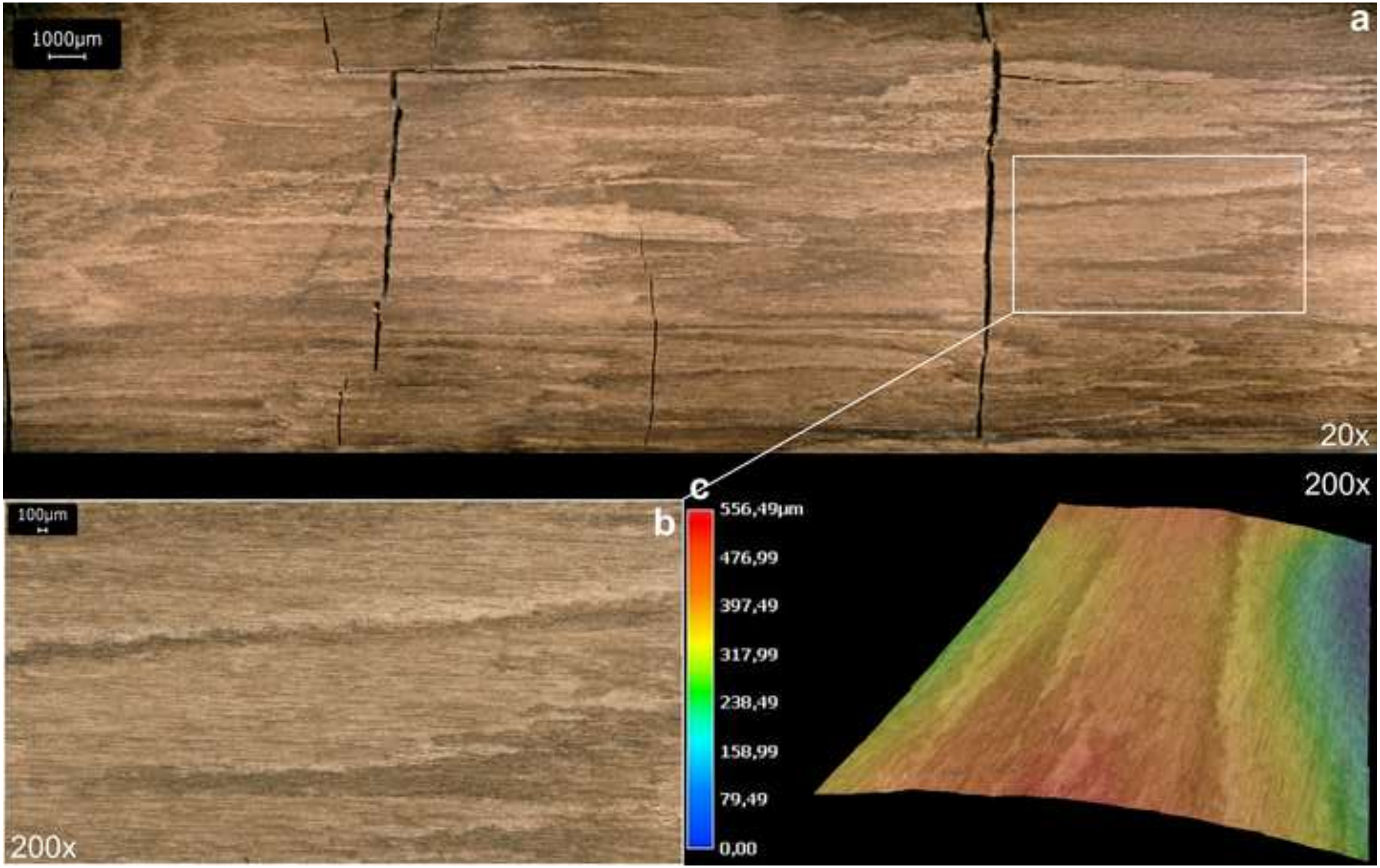

Figure 15 Click here to access/download;Figure;Figure 15.jpg

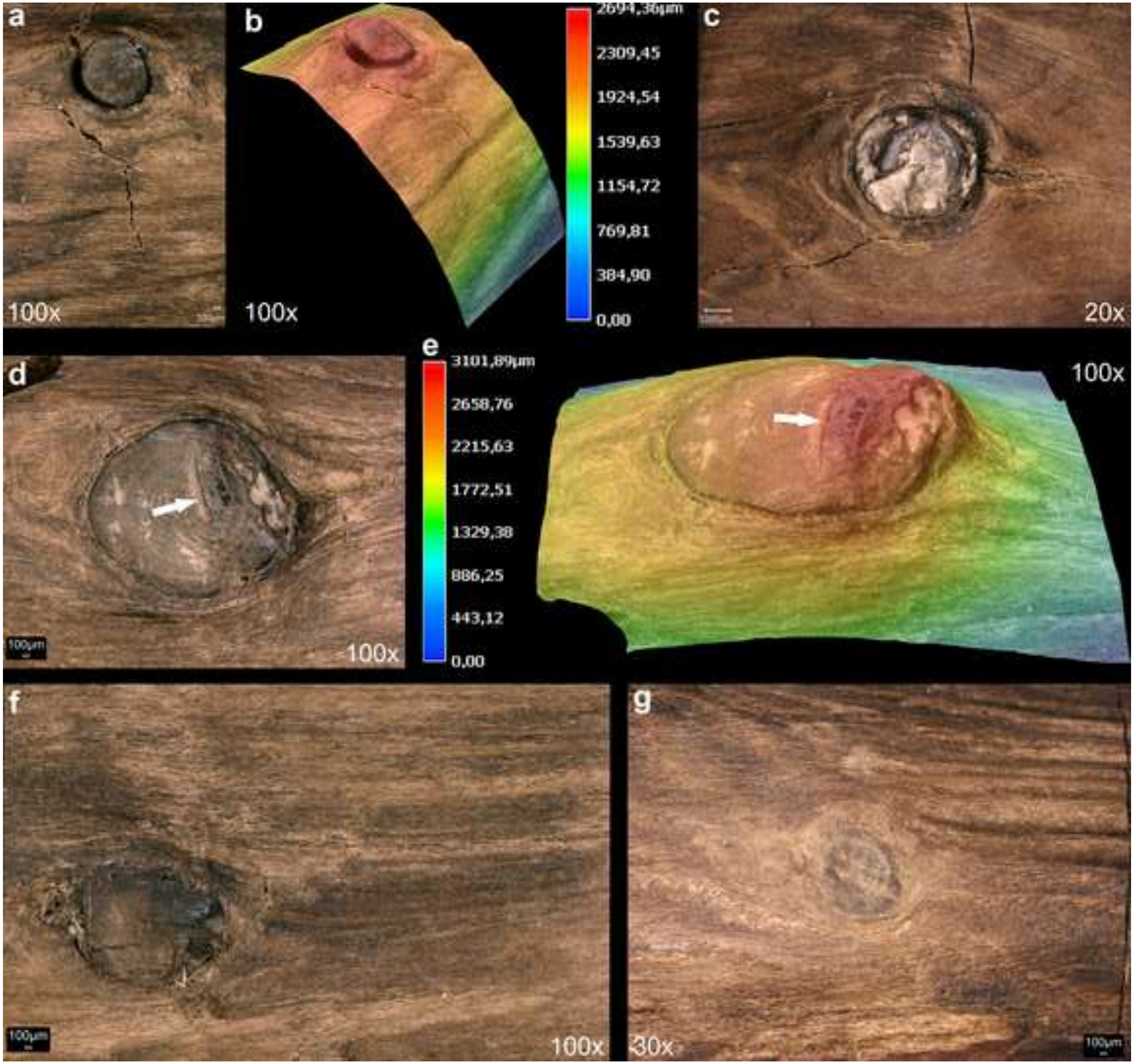

Figure 16

Click here to access/download;Figure;Figure 16.tiff

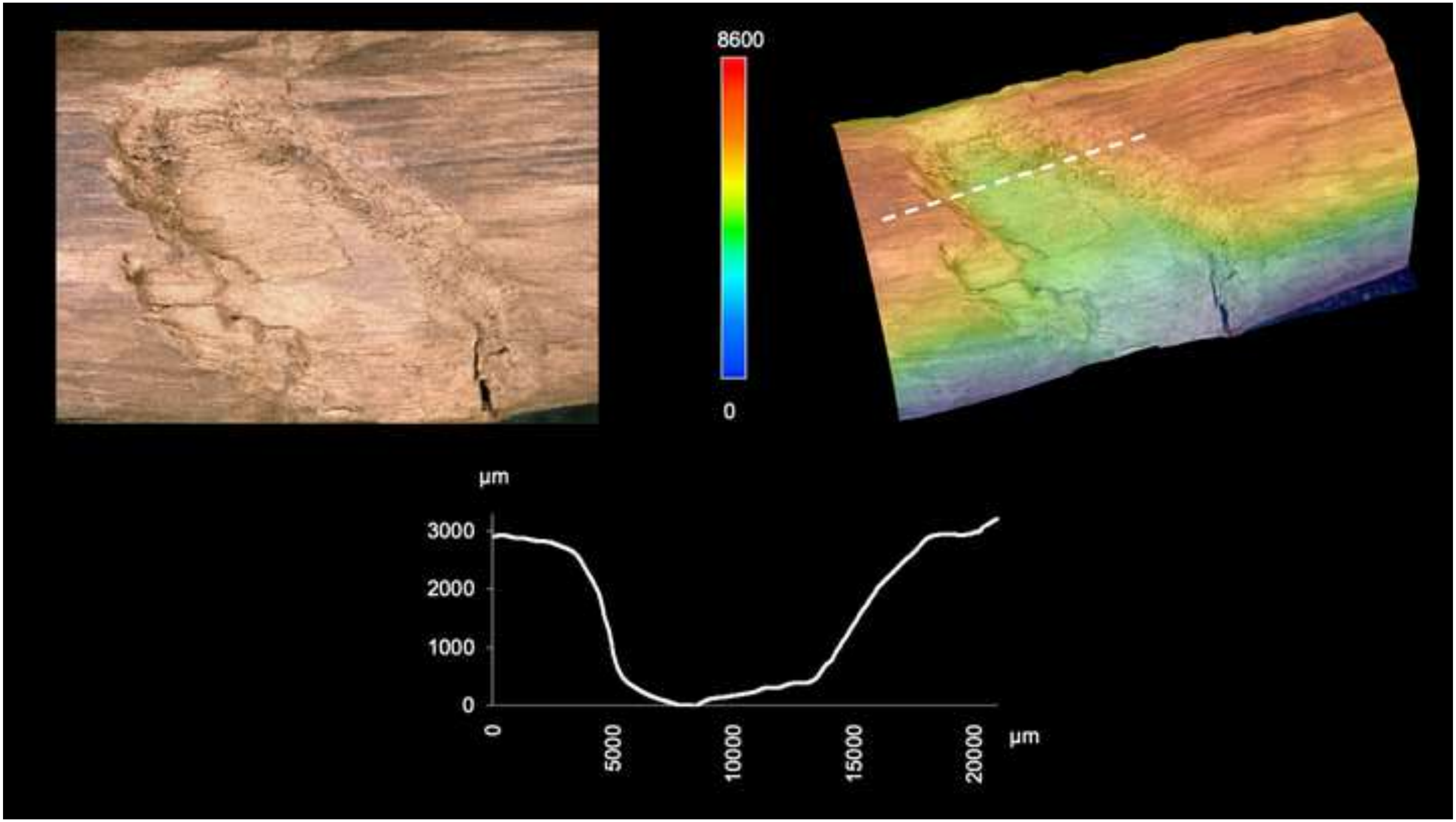

 Click here to access/download;Figure;Figure 17.jpg

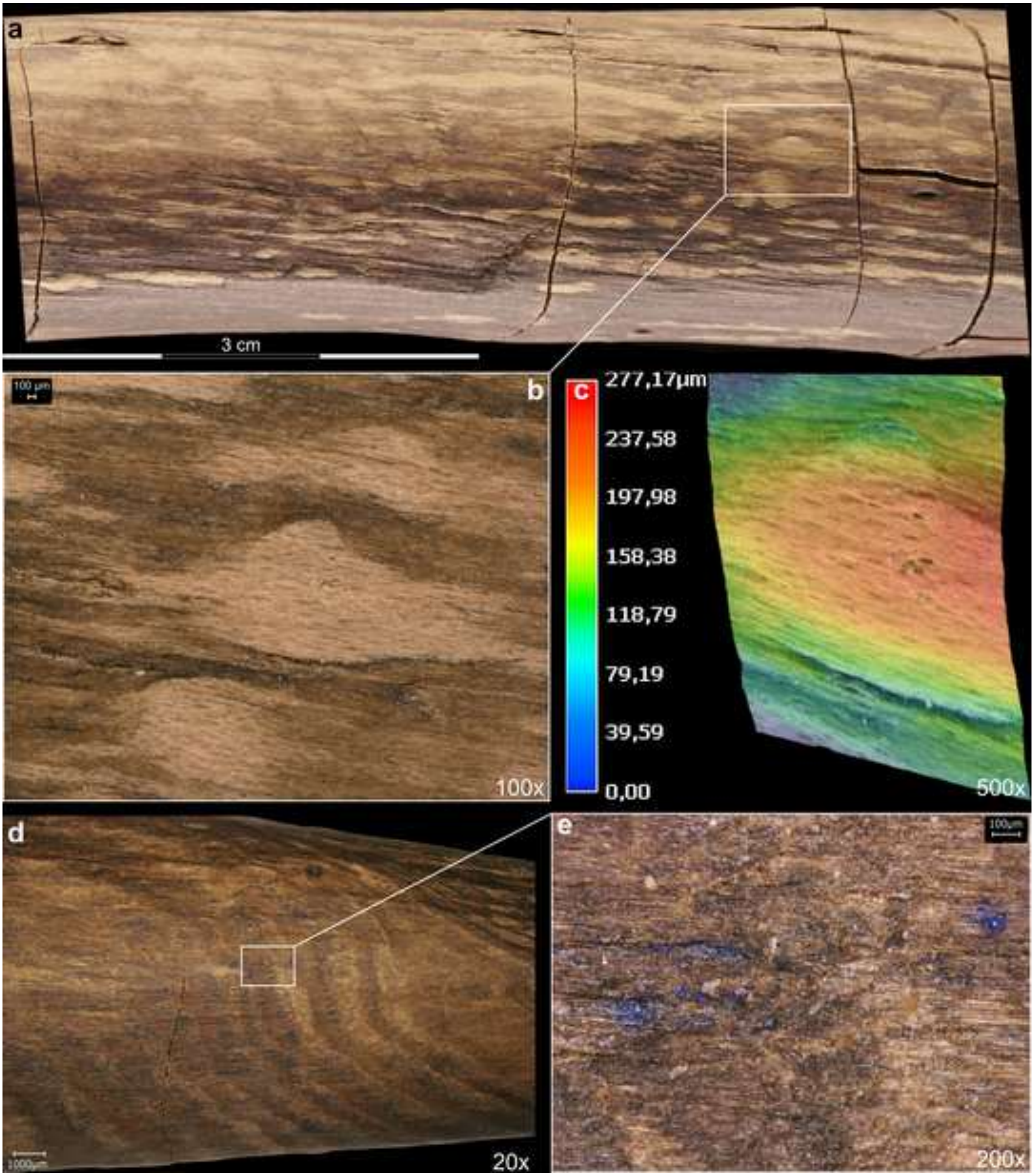

Figure 18

Click here to access/download;Figure;Figure 18.jpg

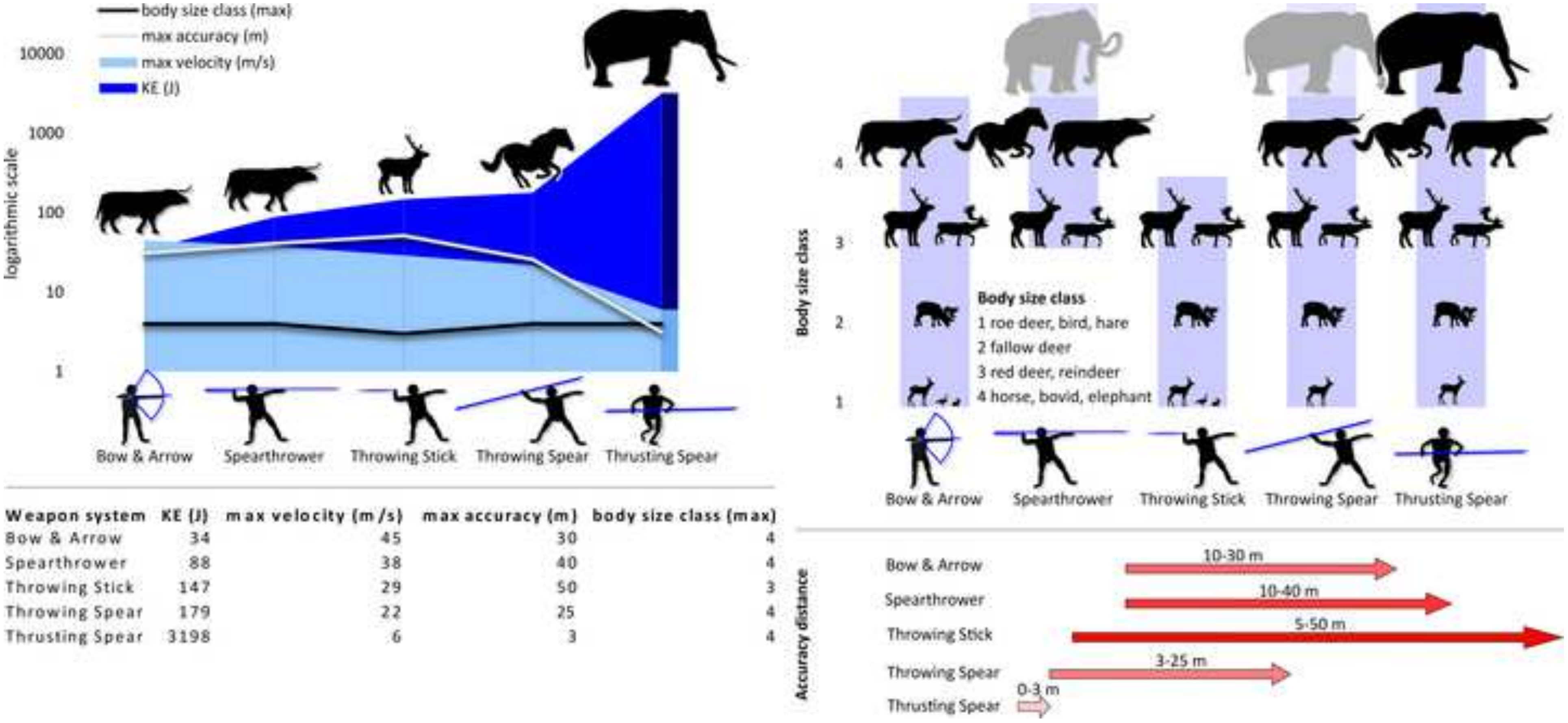

Weapon system	KE (J)	max velocity (m/s)	max accuracy (m)	body size class (max)
Bow & Arrow	34	45	30	4
Spearthrower	88	38	40	4
Throwing Stick	147	29	50	3
Throwing Spear	179	22	25	4
Thrusting Spear	3198	6	3	4

Click here to access/download
Supporting Information
DPS SI To Submit.docx

Click here to access/download
Supporting Information
Dataset 1 Year Ring Analysis.xlsx

Click here to access/download
Supporting Information
Dataset 2 measurements.csv

Dataset 3 microscopy morphometrics

Click here to access/download
Supporting Information
Dataset 3 3D microscopy Morphometrics.xlsx

Click here to access/download
Supporting Information
Dataset 4 Ethnographic Review.csv
